# Prediagnostic primary care consultations and imaging in emergency-diagnosed versus referred patients with lung cancer: a population-based linked data study

**DOI:** 10.3399/BJGP.2025.0369

**Published:** 2026-03-10

**Authors:** Marta Berglund, Becky White, Matthew E Barclay, Emma Whitfield, Cristina Renzi, Meena Rafiq, Neal Navani, Caroline A Thompson, Georgios Lyratzopoulos

**Affiliations:** 1 Epidemiology of Cancer Healthcare & Outcomes Group, Department of Behavioural Science and Health, Institute of Epidemiology and Health Care, University College London, London, UK; 2 School of Medicine, Vita-Salute San Raffaele University, Milan, Italy; 3 Lungs for Living Research Centre, UCL Respiratory, University College London, London, UK; 4 Department of Thoracic Medicine, University College London Hospital, London, UK; 5 Department of Epidemiology, Gillings School of Global Public Health, University of North Carolina at Chapel Hill, Chapel Hill, NC, US; 6 Division of Cancer Epidemiology, Lineberger Comprehensive Cancer Center, University of North Carolina at Chapel Hill, Chapel Hill, NC, US

**Keywords:** cancer diagnosis, emergency, lung cancer, primary health care, general practice, referral and consultation

## Abstract

**Background:**

Emergency diagnosis of lung cancer is common and associated with worse prognosis.

**Aim:**

To compare prediagnostic healthcare use between emergency-diagnosed patients and patients referred routinely or urgently.

**Design and setting:**

Population-based linked English primary care, hospital admission, imaging, and cancer registration data were analysed for patients with lung cancer (2007–2018).

**Method:**

Monthly prediagnosis rates of consultations (for any clinical reason and selected symptoms) and chest imaging by diagnostic route (emergency, routine referral, and urgent referral) were measured. Multivariable Poisson regression estimated route-specific event rates and inflection points.

**Results:**

The study examined 4473 patients with lung cancer with features representative of the nationwide incident cohort, of whom 33% (*n* = 1491) were emergency diagnosed. Most (emergency diagnosis, *n* = 1473/1491; routine referral, *n* = 1023/1026; urgent referral, *n* = 1252/1259; and other, *n* = 684/697) had consulted in primary care in the year prediagnosis, independent of diagnostic route. Consultation and imaging rates increased from 5- and 4-months prediagnosis, respectively, with shorter diagnostic windows in emergency-diagnosed than referred route patients. Compared with emergency-diagnosed patients, referred route patients had higher prediagnostic consultations rates for cough (adjusted incidence rate ratio [aIRR] compared with emergency-diagnosed 1.90, 95% confidence interval [CI] = 1.58 to 2.30 for routinely and 1.94, 95% CI = 1.61 to 2.33 for urgently referred) and chest X-ray use (aIRR 1.91, 95% CI = 1.53 to 2.38 for routinely and 1.77, 95% CI = 1.42 to 2.21 for urgently referred).

**Conclusion:**

Similar or shorter diagnostic windows suggest similar potential for earlier diagnosis among emergency-diagnosed and referred route patients alike. Earlier detection may be supported through improved management of non-specific symptoms, timely follow-up of imaging, and greater access to chest computed tomography. Future research should measure missed diagnostic opportunities to identify clinical actions to further reduce emergency lung cancer diagnoses.

## How this fits in

It has been postulated that emergency diagnoses of cancer (which occurs frequently and confers a poorer prognosis) may relate to suboptimal diagnostic management in primary care, but evidence to support or refute this hypothesis is sparse. This study found that emergency-diagnosed patients with lung cancer were less likely to present with relevant respiratory symptoms and had fewer chest imaging investigations before diagnosis compared with patients diagnosed via referred routes, indicating an important role of disease factors in emergency diagnosis. The study also found that the rate of prediagnostic healthcare activity increased at the same time or closer to diagnosis for emergency-diagnosed patients compared with those diagnosed through referred routes, suggesting a similar potential for earlier diagnosis for all diagnostic routes. Improved imaging follow-up, greater access and use of computed tomography, and greater use of safety netting in primary care could help diagnose lung cancer earlier across all diagnostic routes.

## Introduction

Patients with lung cancer are often diagnosed through an emergency presentation route, for example, after an emergency hospital admission or an emergency department attendance.^1^ Emergency diagnosis is strongly associated with worse survival, independent of stage at diagnosis.^2^ The extent to which emergency diagnoses are avoidable remains unclear, as tumour, patient, and health system factors may all play a role.^3^ Reductions may be possible through improved help-seeking or by enhancing the diagnostic process post-presentation. However, rapidly progressing symptoms in the context of aggressive tumours may also contribute.^3^

In the UK, the NHS provides universal, publicly funded care, with GPs central to cancer recognition and referral. The National Institute for Health and Care Excellence (NICE) guidelines outline symptoms, risk factors, and thresholds for urgent referral (the ‘2-week-wait’ pathway) to support timely diagnosis.^4^ Despite these systems, many cancers are still diagnosed late, often via emergency presentations.

The concept of a ‘diagnostic window’ has been used in cancer and other diseases,^5–9^ representing prediagnostic periods where healthcare use for a patient group increases from baseline, during which there is potential for earlier diagnosis. The time point where rates start to rise is known as the inflection point. Diagnostic windows can be defined by rising rates of events such as GP consultations, hospital visits, prescriptions, or investigations. The presence of diagnostic windows has been described in patients with lung cancer in Denmark,^10,11^ England,^8,12^ Australia,^13^ and New Zealand,^14^ without examining differences by diagnostic route. Such analyses can elucidate likely mechanisms leading to emergency diagnosis, as shown for haematological,^15^ colorectal,^8,9^ and lung cancers.^10,14^

Prior studies have shown patients with emergency-diagnosed colon cancer are less likely to present with alarm symptoms like rectal bleeding than those diagnosed via referrals.^9^ Emergency-diagnosed patients are more likely to have advanced or unknown stage and unspecified tumour type. However, this research relies on recorded and coded primary care symptoms, limiting the analysis to those captured in the data, which may not completely represent patients’ true symptom experiences. These patterns suggest emergency-diagnosed patients may have more aggressive tumours, although patient- and system-level factors may also contribute.

Additionally, patients may have shorter diagnostic intervals because they experience more severe symptoms and aggressive tumour presentation, and so may receive specialist care and treatment sooner. This concept is referred to as the ‘waiting time paradox’, whereby patients with shorter diagnostic intervals have worse outcomes than those with longer diagnostic intervals.^16^

In lung cancer, lower prediagnostic healthcare use rates and shorter diagnostic windows among emergency-diagnosed patients would point to tumour factors influencing the diagnostic route. In contrast, higher prediagnostic healthcare use rates and longer diagnostic windows among emergency-diagnosed patients would suggest opportunities for improving the diagnostic process. This study examined whether prediagnostic healthcare use differed between patients diagnosed as emergencies or through referred routes.

## Method

### Study population and data sources

Patients with lung cancer were identified from a random sample of 1 million patients registered with Clinical Practice Research Datalink (CPRD) GOLD (UK primary care data source, November 2021 build) aged 30–99 years with ≥1 year of registration during January 2007 to October 2018. Patients diagnosed with lung cancer were diagnosed during January 2007 to October 2018, defined using International Classification of Diseases (ICD)-10 codes C33 or C34 in the cancer registry records. Data were linked to the National Cancer Registration and Analysis Service (NCRAS), Hospital Episode Statistics Admitted Patient Care (HES APC), and imaging data (HES DID, available from April 2012 onwards) (see Supplementary Figure S1). Index of Multiple Deprivation (IMD) quintiles (a small-area measure of socioeconomic status) were assigned based on the patient’s residence postcode.

### Explanatory and outcome variables

The main outcome was the diagnostic route by which patients with lung cancer were diagnosed, assigned using NCRAS’s routes-to-diagnosis algorithm.^17^ Diagnosis via an emergency presentation was compared with the two main referred routes: urgent referral for suspected cancer (hereafter denoted as ‘urgent’ referral, and historically known as 2-week-wait referral) or GP routine referral.^18^

Primary explanatory variables were types of healthcare use in the 24 months before diagnosis, excluding the 30 days immediately prediagnosis. First, monthly mean event rates per patient were calculated. Second, diagnostic window length was measured, defined as the month when the inflection point occurred (that is, when healthcare use started to increase from baseline).

Monthly rates (a month is defined as 30 days) of clinical primary care consultations (limited to one per day, excluding administrative encounters);^19,20^ and consultations for six selected relevant symptoms, comprising three respiratory symptoms (cough, dyspnoea, and haemoptysis, assessed individually and grouped) included in referral guidelines for suspected lung cancer,^21^ and three non-localising symptoms (appetite loss, weight loss, and fatigue, assessed as a group) associated with lung cancer were examined.^22^ Symptoms were defined in CPRD using previously published Read (version 2 [v2]) codelists.^23^ Same-day repeat events of the same type were excluded. Chest imaging recorded in HES DID, including chest X-ray and chest computed tomography (CT), using previously published National Interim Clinical Imaging Procedure and Systematised Nomenclature of Medicine codelists were also examined.^24^

### Covariates

Several patient-level factors were included as covariates, including age at diagnosis, gender, and patient’s area-level IMD (2015). Ethnicity was identified from NCRAS, categorised as ‘White’, ‘any other ethnicity’ (‘Asian’, ‘Black’, ‘mixed’, or ‘other’), and ‘unknown’.^25^ Smoking status at time of diagnosis was identified using Read v2 codes in CPRD, categorised as ‘smoker’ (‘current’ or ‘ex-smoker’) and ‘non-smoker’.^26,27^ Chronic obstructive pulmonary disease (COPD) status was identified using Read v2 and ICD-10 codes in CPRD and HES APC, categorised as ‘no COPD’, ‘new-onset COPD’ (diagnosed within 24 months before lung cancer), and ‘pre-existing COPD’ (diagnosed >24 months before lung cancer diagnosis) (see Supplementary Figure S2).^12,28^ Elixhauser comorbidity score was identified using ICD-10 codes in HES APC, excluding codes for COPD and lung cancer, categorised into scores of 0, 1 ,2, or ≥3.^29–31^ Cancer-related factors included cancer stage at diagnosis (categorised as ‘advanced’ for stages 3–4 and ‘non-advanced’ for stages 1–2)^32^ and morphology (non-small-cell and small-cell lung cancer).^33^ A restriction window of 12 months prediagnosis was applied to the Elixhauser scores (see Supplementary Figure S3).^34^ Death within a year post-diagnosis defined using the death date recorded in CPRD was also described. Codelists used in this study are available online (see Data section).

### Statistical analysis

The study examined healthcare use across segmented prediagnosis periods (months 24–1, 24–12, 12–6, and 6–1 before diagnosis) and, in line with previous studies,^6,9^ results for the 12 months prediagnosis where most changes occurred are reported (see Supplementary Figure S4).

Crude rates were calculated as the mean number of events per patient per month. For each month, total events was summed and divided by the number of patients with follow-up. Crude rates were stratified by diagnostic route. For urgently and routinely referred groups, age-standardised rates are also reported using the emergency group’s age distribution as the standard to directly allow for between-route comparisons. The cumulative monthly proportion of patients with ≥1 relevant event in the prediagnostic year was calculated.

Mixed-effects Poisson models (including a random effect for patient)^35^ were fitted to compare prediagnostic rates (mean monthly events per patient) by diagnostic route, using a crude model and adjusted for patient factors (age, gender, ethnicity, IMD, COPD status, smoking status, and Elixhauser score). The emergency-diagnosed group was used as the reference category. Including morphology and stage did not materially affect results and therefore was excluded. The month immediately prediagnosis was excluded from the crude rate modelling.

The maximum likelihood estimation method^20,36^ was used to estimate the inflection points for each healthcare use type by diagnostic route for the entire 12-month period before diagnosis. This method fits a series of Poisson regression models (as above), each fitting a unique inflection-point variable corresponding to a different prediagnosis month. The model with the month with highest log-likelihood denotes the month corresponding to the inflection point. The 95% confidence intervals (CIs) were estimated using bootstrapping. Periods before the inflection points are referred to as background periods.

## Results

### Cohort description

**Figure 1.**
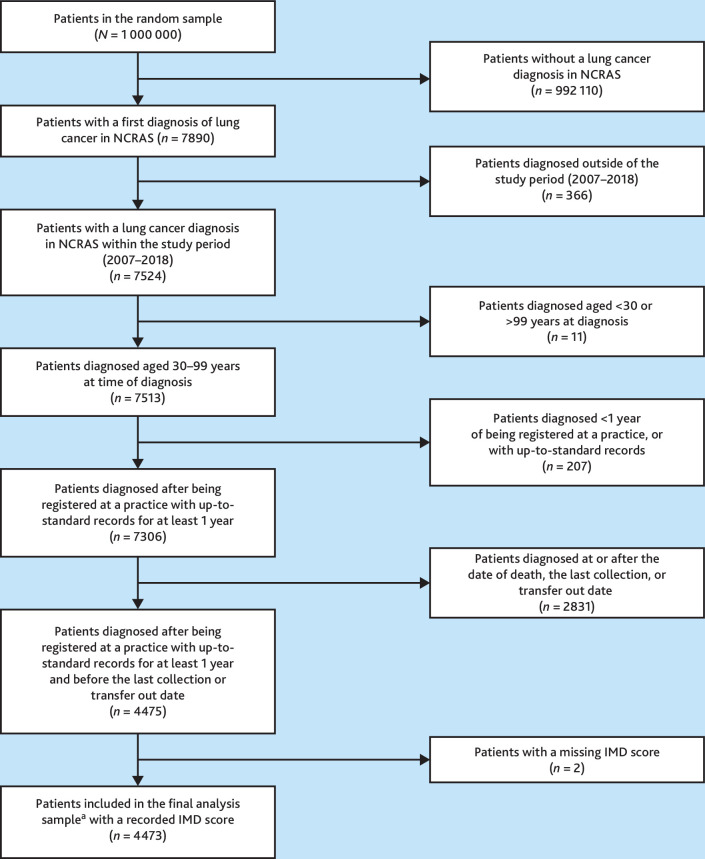
Flowchart describing the cohort selection process. ^a^2059 patients were diagnosed after April 1 2012, and were therefore included in analyses relating to chest imaging. IMD = Index of Multiple Deprivation. NCRAS = National Cancer Registration and Analysis Service.

After exclusions, 4473 patients with lung cancer were included in analysis (Figure 1): 1491 patients (33%) were diagnosed as emergencies, 1259 (28%) through urgent referral, 1026 (23%) through routine referral, and 697 (16%) through other routes (Table 1). Similar proportions of patients presented to primary care before diagnosis across all routes (Table 2). Emergency-diagnosed patients were on average older and more likely to have advanced or unknown stage at diagnosis, tumour of unspecified morphology, pre-existing or new-onset COPD, higher Elixhauser comorbidity score, and to die within a year post-diagnosis compared with referred route patients (Table 1). The regional distribution of patients is presented in Supplementary Table S1.

**Table 1.** Comparison of characteristics of the studied patients with lung cancer by diagnostic route (*N* = 4473)

**Variable**	**Emergency diagnosis** **(** ***n*** **= 1491, 33%), *n* (%)^a^**	**Routine referral** **(** ***n*** **= 1026, 23%), *n* (%)^a^**	**Urgent referral (** ***n*** **= 1259, 28%), *n* (%)^a^**	**Other** **(*n* = 697, 16%),*n* (%)^a,b^**	***P*-value** ^**c**^
**Age at diagnosis, years**					<0.001
Mean (SD)	75 (11)	72 (10)	71 (10)	72 (11)	
Median (IQR), range	76 (68–84), 32–99	73 (65–80), 39–96	71 (63–78), 36–97	72 (65–79), 38–98	
**Age at diagnosis, years**					<0.001
30–64	258 (17)	235 (23)	343 (27)	172 (25)	
65–74	391 (26)	360 (35)	441 (35)	231 (33)	
75–84	508 (34)	334 (33)	363 (29)	220 (32)	
85–99	334 (22)	97 (9)	112 (9)	74 (11)	
**Stage of cancer at diagnosis**					<0.001
Advanced	785 (53)	473 (46)	695 (55)	289 (41)	
Not advanced	106 (7)	210 (20)	214 (17)	142 (20)	
Unknown	600 (40)	343 (33)	350 (28)	266 (38)	
**Gender**					0.130
Female	722 (48)	470 (46)	558 (44)	337 (48)	
Male	769 (52)	556 (54)	701 (56)	360 (52)	
**Ethnicity**					<0.001
White	1388 (93)	962 (94)	1198 (95)	618 (89)	
Any other ethnicity	81 (5)	45 (4)	48 (4)	45 (6)	
Unknown	22 (1)	19 (2)	13 (1)	34 (5)	
**IMD**					0.500
1 (least deprived)	239 (16)	191 (19)	200 (16)	134 (19)	
2	271 (18)	205 (20)	239 (19)	137 (20)	
3	319 (21)	209 (20)	267 (21)	148 (21)	
4	325 (22)	215 (21)	284 (23)	144 (21)	
5 (most deprived)	337 (23)	206 (20)	269 (21)	134 (19)	
**Smoker status**					<0.001
Smoker	1315 (88)	920 (90)	1164 (92)	605 (87)	
Non-smoker	176 (12)	106 (10)	95 (8)	92 (13)	
**COPD status**					0.001
New-onset COPD (during months –24 to 0)	238 (16)	148 (14)	150 (12)	84 (12)	
Pre-existing COPD (at –24 months or earlier)	356 (24)	222 (22)	252 (20)	152 (22)	
No COPD	897 (60)	656 (64)	857 (68)	461 (66)	
**Elixhauser comorbidity score**					<0.001
0	144 (10)	259 (25)	439 (35)	174 (25)	
1	161 (11)	184 (18)	301 (24)	121 (17)	
2	257 (17)	195 (19)	202 (16)	116 (17)	
≥3	929 (62)	388 (38)	317 (25)	286 (41)	
**Survival at 1 year from diagnosis**					<0.001
Died	1176 (79)	528 (51)	634 (50)	351 (50)	
Alive	315 (21)	498 (49)	625 (50)	346 (50)	
**Morphology**					<0.001
SCLC	133 (9)	85 (8)	170 (14)	59 (8)	
NSCLC	684 (46)	718 (70)	915 (73)	462 (66)	
Unspecified	674 (45)	223 (22)	174 (14)	176 (25)	

^a^Unless otherwise stated. ^b^Other routes include: inpatient elective, other managed pathway, DCO (diagnosis by death certificate only), and unknown.^17^ ^c^One-way ANOVA for continuous age at diagnosis; otherwise Pearson’s χ^2^ test. COPD = chronic obstructive pulmonary disease. IQR = interquartile range. NSCLC = non-small-cell lung cancer. SCLC = small-cell lung cancer. SD = standard deviation.

**Table 2.** Summary of the proportion of patients and mean events per patient per month of the included healthcare use measures 12 to 1 month prediagnosis by diagnostic route

**Healthcare event type**	**Emergency diagnosis** **(** ***n*** **= 1491, 33%)**	**Routine referral** **(** ***n*** **= 1026, 23%)**	**Urgent referral** **(** ***n*** **= 1259, 28%)**	**Other^a^** **(** ***n*** **= 697, 16%)**	***P*-value^b^**
**Proportion of patients with ≥1 event, *n* (%)**					
Consultations					
Any	1473 (98.8)	1023 (99.7)	1252 (99.4)	684 (98.1)	<0.001
With any selected six relevant symptoms^c^	859 (57.6)	669 (65.2)	891 (70.8)	400 (57.4)	<0.001
With any selected three respiratory symptoms^d^	791 (53.1)	610 (59.5)	833 (66.2)	355 (50.9)	<0.001
With cough	472 (31.7)	433 (42.2)	606 (48.1)	223 (32.0)	<0.001
With dyspnoea	533 (35.7)	327 (31.9)	367 (29.2)	210 (30.1)	<0.001
With haemoptysis	35 (2.3)	71 (6.9)	104 (8.3)	25 (3.6)	<0.001
Imaging events^e^					
Any chest imaging	604 (88.8)	449 (91.1)	552 (92.8)	244 (83.8)	<0.001
Chest X-ray	577 (84.9)	399 (80.9)	513 (86.2)	224 (77.0)	<0.001
Chest CT	341 (50.1)	353 (71.6)	465 (78.2)	179 (61.5)	<0.001
**Events per patient per month, mean (95% CI)^f^**					
Consultations					
Any	1.10 (1.02 to 1.17)	1.21 (1.11 to 1.30)	0.93 (0.86 to 1.00)	0.99 (0.89 to 1.09)	—
With any selected six relevant symptoms^c^	0.06 (0.05 to 0.07)	0.09 (0.07 to 0.11)	0.08 (0.06 to 0.10)	0.07 (0.05 to 0.09)	—
With any selected three respiratory symptoms^d^	0.05 (0.05 to 0.07)	0.05 (0.04 to 0.07)	0.05 (0.04 to 0.07)	0.06 (0.04 to 0.08)	—
With cough	0.03 (0.02 to 0.03)	0.05 (0.03 to 0.06)	0.05 (0.04 to 0.06)	0.04 (0.02 to 0.05)	—
With dyspnoea	0.03 (0.02 to 0.04)	0.03 (0.02 to 0.04)	0.02 (0.01 to 0.03)	0.03 (0.01 to 0.04)	—
With haemoptysis	0.00 (0.00 to 0.00)	0.01 (0.00 to 0.01)	0.00 (0.00 to 0.01)	0.00 (0.00 to 0.00)	—
Imaging events^e^					
Any chest imaging	0.04 (0.02 to 0.05)	0.06 (0.04 to 0.07)	0.04 (0.03 to 0.05)	0.05 (0.03 to 0.06)	—
Chest X-ray	0.03 (0.02 to 0.04)	0.04 (0.03 to 0.05)	0.03 (0.02 to 0.04)	0.03 (0.02 to 0.05)	—
Chest CT	0.01 (0.00 to 0.01)	0.02 (0.01 to 0.03)	0.01 (0.01 to 0.01)	0.01 (0.01 to 0.02)	—

^a^Other routes include: inpatient elective, other managed pathway, DCO (diagnosis by death certificate only), and unknown.^17^ ^b^One-way ANOVA. ^c^Symptoms include appetite loss, weight loss, fatigue, cough, dyspnoea, and haemoptysis. ^d^Symptoms include cough, dyspnoea, and haemoptysis. ^e^Proportions calculated out of all patients diagnosed after 1 April 2012 (emergency presentation, *n* = 680; routine referral, *n *= 493; urgent referral, *n *= 595; and other, *n *= 291). ^f^Excluding the month immediately before diagnosis. CT = computed tomography.

### Diagnostic window length

**Figure 2.**
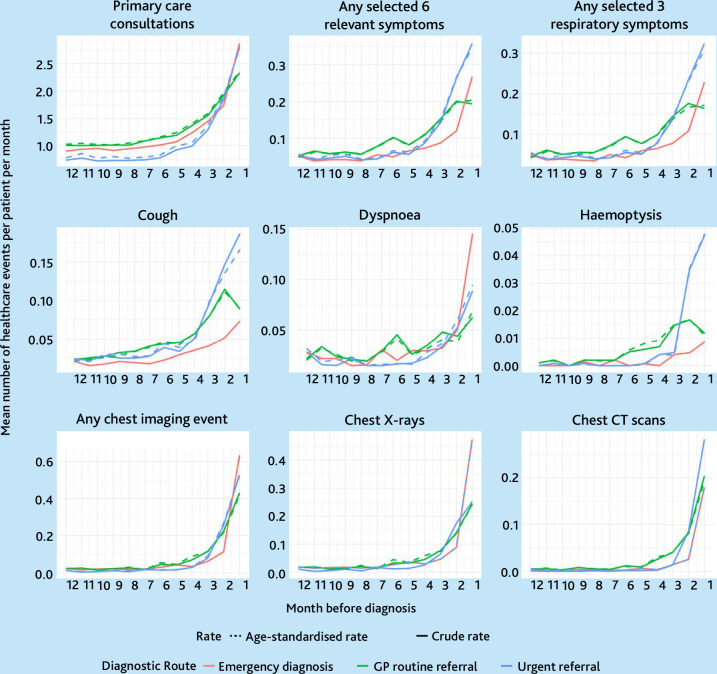
Rates of healthcare use per patient per month 12 months to 1 day before diagnosis by three main diagnostic routes, age-standardised in reference to the emergency-diagnosed group. Y-axis scale differs substantially between the panels/type of healthcare event visualised. The rates were modelled using Poisson regression with data from 12 to 1 month before diagnosis adjusted for patient factors (age at diagnosis, gender, ethnicity, Index of Multiple Deprivation, smoking status, chronic obstructive pulmonary disease status, and Elixhauser comorbidity score at time of diagnosis). Any selected six symptoms include: appetite loss, weight loss, fatigue, cough, dyspnoea, and haemoptysis. Any selected three respiratory symptoms include: cough, dyspnoea, and haemoptysis. CT = computed tomography.

Diagnostic windows were present for all patient groups defined by diagnostic route and were consistently shorter or of equal length for emergency-diagnosed compared with referred route patients (Figure 2 and Table 3). For example, consultations for any reason began to increase from 5 months prediagnosis for both emergency-diagnosed and urgently referred patients, and from 7 months for routinely referred patients. Similarly, corresponding inflection points for consultations with any of three respiratory symptoms (cough, dyspnoea, or haemoptysis) were 5, 10, and 6 months, for emergency-diagnosed, routinely referred, and urgently referred patients, respectively.

**Table 3.** Summary of the measures of healthcare use by diagnostic route including the prediagnostic rates and inflection points within 12 to 1 month before lung cancer diagnosis by diagnostic route compared with emergency-diagnosed patients

**Healthcare event type**	**Adjusted IRR (95% CI)** ^**a**^			**Month of inflection-point estimate (95% CI)**		
	**Emergency diagnosed**	**Routinely referred**	**Urgently referred**	**Emergency diagnosed**	**Routinely referred**	**Urgently referred**
Primary care consultations	1 (Ref)	1.34 (1.26 to 1.43)^b^	1.11 (1.04 to 1.18)^b^	5 (4.99 to 5.01)	7 (6.96 to 7.04)^c^	5 (4.99 to 5.01)^d^
Consultations with any selected six relevant symptoms^e^	1 (Ref)	1.71 (1.51 to 1.94)^b^	1.59 (1.40 to 1.79)^b^	5 (4.95 to 5.05)	9 (8.93 to 9.07)^c^	6 (5.97 to 6.03)^d^
Consultations with any selected three respiratory symptoms^f^	1 (Ref)	1.72 (1.51 to 1.96)^b^	1.62 (1.42 to 1.84)^b^	5 (4.95 to 5.05)	10 (9.93 to 10.07)^c^	6 (5.97 to 6.03)^c^
Consultations with cough	1 (Ref)	1.90 (1.58 to 2.30)^b^	1.94 (1.61 to 2.33)^b^	7 (6.94 to 7.06)	10 (9.93 to 10.07)^c^	7 (6.96 to 7.04)^d^
Consultations with dyspnoea	1 (Ref)	1.33 (1.11 to 1.61)^g^	1.07 (0.88 to 1.30)^g^	3 (2.96 to 3.04)	9 (8.88 to 9.12)^c^	5 (4.96 to 5.04)^c^
Consultations with haemoptysis	1 (Ref)	3.25 (1.33 to 7.96)^g^	2.92 (1.19 to 7.18)^g^	5 (4.88 to 5.12)	10 (9.93 to 10.07)^c^	7 (6.94 to 7.06)^c^
Any chest imaging events	1 (Ref)	2.45 (1.97 to 3.05)^b^	1.95 (1.56 to 2.25)^b^	4 (3.98 to 4.02)	7 (6.97 to 7.03)^c^	6 (5.98 to 6.02)^d^
Chest X-rays	1 (Ref)	1.91 (1.53 to 2.38)^b^	1.77 (1.42 to 2.21)^b^	4 (3.97 to 4.03)	7 (6.96 to 7.04)^c^	6 (5.96 to 6.04)^d^
Chest CT	1 (Ref)	3.39 (2.47 to 4.66)^b^	2.07 (1.48 to 2.90)^b^	4 (3.99 to 4.01)	6 (5.96 to 6.04)^d^	6 (5.97 to 6.03)^d^

^a^The adjusted IRR values show the Poisson modelling results using data from 12 to 1 month before diagnosis adjusted for patient factors (age at diagnosis, gender, ethnicity, Index of Multiple Deprivation, smoking status, chronic obstructive pulmonary disease status, and Elixhauser comorbidity score at time of diagnosis). ^b^Significant positive association (*P*<0.05). ^c^Inflection points occurring ≥2 months before those for emergency presentations. ^d^A difference in the inflection points <2 months, considered equal compared with emergency presentations. ^e^Symptoms include appetite loss, weight loss, fatigue, cough, dyspnoea, and haemoptysis. ^f^Symptoms include cough, dyspnoea, and haemoptysis. ^g^Results with weak evidence (*P*>0.05). IRR = incidence rate ratio. Ref = reference.

### Overall trends in the prediagnostic rates of healthcare use by route

**Figure 3.**
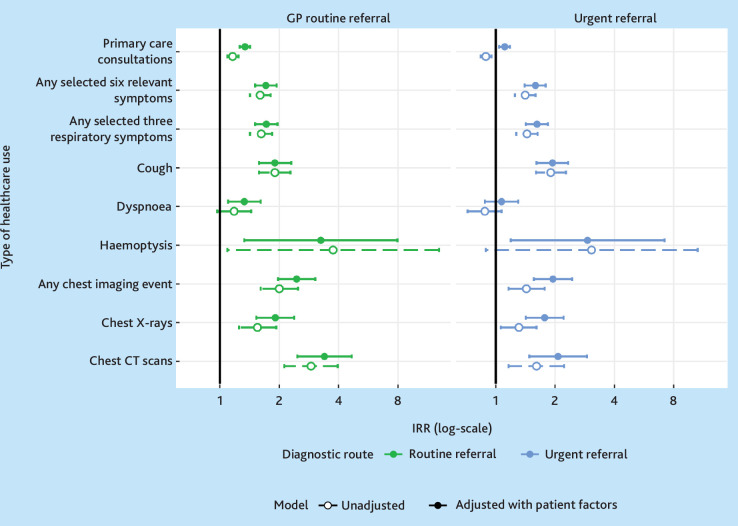
Unadjusted and adjusted Poisson model results showing the incidence rate ratios (IRRs, log-scale) for healthcare use by type 12 months to 1 month before diagnosis (with emergency diagnosed as the reference category). Log transformed data are shown for the economy of visualisation; exact values are included in Table 3. Any selected six symptoms include: appetite loss, weight loss, fatigue, cough, dyspnoea, and haemoptysis. Any selected three respiratory symptoms include: cough, dyspnoea, and haemoptysis. CT = computed tomography.

In the 12 months leading up to (excluding the month immediately before) diagnosis, emergency-diagnosed patients had consultation or imaging rates not discernible from those of patients diagnosed through referred routes (Table 2). Emergency-diagnosed patients had somewhat lower age-standardised rates of prediagnostic consultations or imaging in the year prediagnosis compared with urgently or routinely referred patients, for whom adjusted incidence rate ratios (aIRRs) for both consultations and imaging were consistently higher, often significantly, than those of emergency-diagnosed patients (Supplementary Tables S2–S10, Table 3, and Figure 3). Additional analyses for segmented prediagnostic periods (months 24–1, 24–12, 12–6, and 6–1) across all models and adjustment types are presented in Supplementary Tables S11–S16, showing broadly consistent healthcare use patterns by diagnostic route as reported in the main analysis.

### Rates of consultations for any reason and rates of chest imaging

After adjustment, prediagnostic consultation rates for any clinical reason were lower in emergency-diagnosed patients (aIRR for routinely referred versus emergency diagnosed 1.34, 95% CI = 1.26 to 1.43; aIRR for urgently referred versus emergency diagnosed 1.11, 95% CI = 1.04 to 1.18). Similar but more pronounced differences by diagnostic route were observed for chest imaging activity (aIRR for routinely referred versus emergency diagnosed 2.45, 95% CI = 1.97 to 3.05; aIRR for urgently referred versus emergency diagnosed 1.95, 95% CI = 1.56 to 2.25) (Table 3 and Figure 3).

### Rates of consultations for selected respiratory symptoms

Emergency-diagnosed patients had lower prediagnostic consultation rates for any of three respiratory symptoms (cough, dyspnoea, and haemoptysis) compared with routinely or urgently referred patients. This association remained significant after adjustment, for both routinely (aIRR versus emergency diagnosed 1.72, 95% CI = 1.51 to 1.96) and urgently referred patients (aIRR versus emergency diagnosed 1.62, 95% CI = 1.42 to 1.84). The results were similar for prediagnostic consultation rates with any of the selected six relevant symptoms. Considering consultations for each symptom individually, adjusted prediagnostic consultation rates for cough were lower among emergency-diagnosed patients (aIRR for routinely referred versus emergency diagnosed 1.90, 95% CI = 1.58 to 2.30; aIRR for urgently referred versus emergency-diagnosed 1.94, 95% CI = 1.61 to 2.33). There was inconsistent or weak statistical evidence for variation by diagnostic route in adjusted prediagnostic consultation rates for haemoptysis or dyspnoea (Table 3 and Figure 3).

## Discussion

### Summary

Diagnostic windows of appreciable length were observed in patients with lung cancer, defined by both consultations and imaging activity. In the year prediagnosis, emergency-diagnosed patients had lower adjusted rates of consultations and chest imaging than those diagnosed via urgent or routine referrals. Improving referral pathways and post-imaging follow-up may support earlier diagnosis across routes, although the overall similar rates of prediagnostic primary care contacts across routes limit opportunities for targetted interventions. Surprisingly, as denoted by diagnostic windows of similar length, this potential did not vary substantially between diagnostic routes.

### Strengths and limitations

A representative primary care cohort with linked healthcare data was analysed but the analysis was limited to variables recorded in electronic patient records. This leaves scope for residual confounding from unmeasured variables. Specifically, the findings are susceptible to ‘confounding by indication’, whereby unaccounted-for case-mix differences may influence both diagnostic route and diagnostic windows. The analysis relied on coded rather than free-text data,^37^ and imaging results were unavailable. The findings are limited to those diagnosed with lung cancer, as the study aimed to compare diagnostic windows by route within this group, it did not examine healthcare use patterns in a reference population. Although more recent datasets, such as CPRD Aurum, could be used in future research, cancer registry linkage is currently available only up to 2021. Therefore, major changes in the findings are unlikely, apart from potential effects related to the COVID-19 pandemic. Incorporating additional data such as referral source, prescriptions, and test results could improve understanding of prediagnostic healthcare use and missed diagnostic opportunities.

The maximum likelihood estimation method used to estimate the inflection points assumes a linear trend in healthcare use post-inflection point. The model assumed linear relationships between the covariates and healthcare use, and did not include possible interaction terms between variables.^38^ A case-only design without population controls was used, as the comparisons related to patients with lung cancer diagnosed through different routes.^38^

Emergency-diagnosed patients have higher 1-year mortality even after adjusting for stage and morphology.^39,40^ Although comparing treatment by diagnostic route may clarify factors leading to poorer outcomes, such analyses were beyond the scope of this study.

### Comparisons with existing literature

Consistent with prior studies (measuring primary care consultations, prescriptions, blood tests, and chest X-rays), increased healthcare use was observed in the current study beginning 4–6 months prediagnosis.^11,13,40^ However, the current study additionally profiled diagnostic windows and related healthcare activity by diagnostic route for the first time, to the best of the authors’ knowledge, in patients with lung cancer. A similar approach has been recently reported for diagnostic windows for patients with haematological cancers diagnosed through different routes.^15^

The notion that emergency-diagnosed patients consult infrequently in primary care is not supported by the current study’s findings, showing similar background consultation patterns across routes.^41^ Furthermore, the finding of lower healthcare use (any consultations, consultations with relevant symptoms, and chest imaging activity) during the diagnostic window among emergency-diagnosed patients aligns with studies on other cancer sites.^8,9,14,15,31^

Diagnostic window lengths for emergency-diagnosed patients were similar or shorter than for referred route patients. Patients with lung cancer often present with non-specific symptoms such as cough, dyspnoea, respiratory infections, and chest pain.^42–44^ Consistent with previous studies, the current study found that haemoptysis, the respiratory symptom with the highest positive predictive value for lung cancer, was relatively rare.^45,46^ These findings concord with those observed for colorectal cancer, where similar diagnostic window length and fewer alarm symptoms (such as rectal bleeding) were reported in emergency-diagnosed patients.^8,9^

Fewer chest imaging events were observed among emergency-diagnosed patients. Patients with higher primary care engagement and guideline-concordant care are more likely to receive timely imaging.^11,24,47^

Tumour stage and morphology are associated with emergency diagnosis,^14,39^ but adjusting for them did not materially alter the findings regarding event rates.

### Implications for research and practice

Emergency-diagnosed patients consulted less with the selected symptoms and underwent fewer prediagnostic imaging investigations than those diagnosed through urgent or routine referrals. This may reflect genuine differences in tumour presentation across diagnostic routes, or patient-related factors such as health literacy (for example, poorer recognition of significant symptoms) and delayed help-seeking despite symptom experience.^48–51^ Health literacy and intervals from symptom onset to help-seeking are not captured in routine health record data, yet are likely to contribute to the observed differences and should be explored in future research.

This study provides population-level evidence on diagnostic routes for lung cancer and highlights potential for earlier diagnosis. Substantial diagnostic windows across all routes suggest the need for generic improvements, such as better risk assessment of non-specific symptoms and greater imaging use. For emergency-diagnosed patients, fewer symptomatic consultations mean fewer primary care opportunities to act on signs that might have prompted non-emergency referral. Further work should examine where specific actions, particularly for chest imaging results, could have expedited diagnosis.

As around a fifth of lung cancers are not detectable via chest X-ray at the time of symptomatic presentation,^47^ the observed diagnostic windows defined by chest imaging are likely to reflect false-negative investigations, underscoring the need for proactive follow-up (‘safety netting’) and wider chest CT access (for example, as a follow-up chest imaging test, after initial negative chest X-ray when symptoms and diagnostic suspicion prevail).^50^ Expanding GP access to chest CTs, which have greater sensitivity than chest X-rays, could improve early lung cancer detection and reduce emergency diagnoses.^51^ Similar findings in urological cancers show that use of less-sensitive imaging modalities can hinder timely diagnosis.^52^

Although rare, delays in acting on abnormal chest X-ray findings may represent missed diagnostic opportunities; future research should aim to quantify whether and how often such safety incidents might be occurring.^40^ Evaluating adherence to referral/imaging guidelines and detailed event analysis may identify missed diagnostic opportunities and improve care across diagnostic routes.^24,53^ Such studies can inform interventions targeting potentially avoidable emergency diagnoses.

Lung cancer screening could reduce emergency diagnoses, as shown in colorectal cancer.^53–56^ In the current cohort, 2171/4473, 49% of patients (including *n* = 573/1491, 38% of emergency-diagnosed patients), were aged 55–74 years, within the eligible screening range depending on smoking history. However, participation in screening is lower among socioeconomically deprived groups, who are more likely to be diagnosed via an emergency route.^57^ Thus, although screening may reduce emergency presentations, equitable uptake is essential to avoid widening inequalities in lung cancer diagnosis.

In conclusion, comparing prediagnostic healthcare use between emergency-diagnosed and referred patients with lung cancer revealed no clear aetiological targets to reduce emergency diagnosis. Instead, the study’s findings suggest potential to expedite diagnosis across all groups. In particular, through improved management of non-specific symptoms, timely follow-up of imaging, and greater access to chest CTs for suspected cancer.

## Data

Codelists used in this study are available online at https://github.com/martaberglund/EPlungcancer-phenotypes.git. Data cannot be shared by the researchers, but access to Clinical Practice Research Datalink (CPRD) data is available subject to protocol approval via CPRD’s Research Data Governance process; see https://cprd.com/data-access for further details.
