# Pre-diagnostic primary care consultations and imaging in emergency-diagnosed vs referred lung cancer patients

**DOI:** 10.3399/BJGP.2025.0369

**Published:** 2026-03-26

**Authors:** Marta Berglund, Becky White, Matthew E Barclay, Emma Whitfield, Cristina Renzi, Meena Rafiq, Neal Navani, Caroline A Thompson, Georgios Lyratzopoulos

**Affiliations:** 1. Epidemiology of Cancer Healthcare & Outcomes (ECHO) Group, Dept. of Behavioural Science and Health, Institute of Epidemiology and Health Care (IEHC), University College London, London, UK; 2. School of Medicine, University Vita-Salute San Raffaele, Milan, Italy; 3. Lungs for Living Research Centre, UCL Respiratory, University College London, UK; Department of Thoracic Medicine, University College London Hospital, London, UK; 4. Department of Epidemiology, Gillings School of Global Public Health, The University of North Carolina at Chapel Hill, Chapel Hill, NC, USA; 5. Division of Cancer Epidemiology, Lineberger Comprehensive Cancer Center, University of North Carolina at Chapel Hill, Chapel Hill, NC, USA

**Keywords:** Cancer diagnosis, Primary care, Lung, Consultations, Emergency, Referral

## Abstract

**Background:**

Emergency diagnosis of lung cancer is common and associated with worse prognosis.

**Aim:**

To compare pre-diagnostic healthcare use between emergency-diagnosed and patients referred routinely or urgently.

**Design and Setting:**

We analysed population-based linked English primary care, hospital admission, imaging, and cancer registration data on patients with lung cancer (2007–2018).

**Methods:**

We measured monthly pre-diagnosis rates of consultations (for any clinical reason and selected symptoms) and chest imaging by diagnostic route (emergency, routine referral, urgent referral. Multivariable Poisson regression estimated route-specific event rates and inflection points.

**Results:**

We examined 4,473 patients with lung cancer with features representative of the nationwide incident cohort, of whom 33% (n=1,491) were emergency-diagnosed. Nearly all patients (98%) had consulted in primary care in the year pre-diagnosis, independently of diagnostic route. Consultation and imaging rates increased from 5- and 4-months pre-diagnosis, respectively, with shorter diagnostic windows in emergency-diagnosed than referred route patients. Compared with emergency-diagnosed, referred route patients had higher pre-diagnostic consultations rates for cough (adjusted Incidence Rate Ratio [aIRR] compared with emergency-diagnosed:1.90 for routinely and 1.94 for urgently referred patients) and chest X-ray use (aIRR:1.91 and 1.77, respectively).

**Conclusion:**

Similar or shorter diagnostic windows suggest similar potential for earlier diagnosis among emergency-diagnosed and referred route patients alike. Earlier detection may be supported through improved management of non-specific symptoms, timely follow-up of imaging, and greater access to chest CTs. Future research should measure missed diagnostic opportunities to identify clinical actions to further reduce emergency lung cancer diagnoses.

## Summary sentence

Emergency-diagnosed lung cancer patients have different pre-diagnostic healthcare use activity compared to those diagnosed after a GP referral.

## 1. INTRODUCTION

Patients with lung cancer are often diagnosed through an emergency presentation route, for example, after an emergency hospital admission or an emergency department attendance^(1)^. Emergency diagnosis is strongly associated with worse survival, independently of stage at diagnosis^(2)^. The extent to which emergency diagnoses are avoidable remains unclear, as tumour, patient, and health system factors may all play a role^(3)^. Reductions may be possible through improved help-seeking or by enhancing the diagnostic process post-presentation. However, rapidly progressing symptoms in the context of aggressive tumours may also contribute^(3)^.

In the UK, the National Health Service (NHS) provides universal, publicly funded care, with general practitioners (GPs) central to cancer recognition and referral. The National Institute for Health and Care Excellence (NICE) guidelines outline symptoms, risk factors, and thresholds for urgent referral (the ‘two-week-wait’ pathway) to support timely diagnosis^(4)^. Despite these systems, many cancers are still diagnosed late, often via emergency presentations.

The concept of a ‘diagnostic window’ has been used in cancer and other diseases^(5–9)^, representing pre-diagnostic periods where healthcare use for a patient group increases from baseline, during which there is potential for earlier diagnosis. The time point where rates start to rise is known as the inflection point. Diagnostic windows can be defined by rising rates of events such as GP consultations, hospital visits, prescriptions, or investigations. The presence of diagnostic windows has been described in patients with lung cancer in Denmark^(10,11)^, England^(8,12)^, Australia^(13)^, and New Zealand^(14)^, without examining differences by diagnostic route. Such analyses can elucidate likely mechanisms leading to emergency diagnosis, as shown for haematological^(15)^, colorectal^(8,9)^, and lung cancers^(8,10,14)^.

Prior studies have shown emergency-diagnosed colon cancer patients are less likely to present with alarm symptoms like rectal bleeding than those diagnosed via referrals^(9)^. Emergency-diagnosed patients are more likely to have advanced or unknown stage and unspecified tumour type. However, this research relies on recorded and coded primary care symptoms, limiting the analysis to those captured in the data, which may not completely represent patients’ true symptom experiences. These patterns suggest emergency-diagnosed patients may have more aggressive tumours, though patient and system-level factors may also contribute.

Additionally, patients may have shorter diagnostic intervals because they experience more severe symptoms and aggressive tumour presentation, and so may receive specialist care and treatment sooner. This concept is referred to as the ‘waiting time paradox’, whereby patients with shorter diagnostic intervals have worse outcomes than those with longer diagnostic intervals^(16)^.

In lung cancer, lower pre-diagnostic healthcare use rates and shorter diagnostic windows among emergency-diagnosed patients would point to tumour factors influencing the diagnostic route. In contrast, higher pre-diagnostic healthcare use rates and longer diagnostic windows among emergency-diagnosed would suggest opportunities for improving the diagnostic process. This study examines whether pre-diagnostic healthcare use differed between patients diagnosed as emergencies or through referred routes.

## 2. METHODS

### Study population and data sources

We identified lung cancer patients from a random sample of one million patients registered with Clinical Practice Research Datalink (CPRD) GOLD (UK primary care data source, November 2021 build) aged 30–99 with at least one year of registration during Jan 2007-Oct 2018. Lung cancer patients were diagnosed during Jan 2007-Oct 2018, defined using ICD-10 codes C33 or C34 in the cancer registry records. Data were linked to the National Cancer Registration and Analysis Service (NCRAS), Hospital Episodes Statistics Admitted Patient Care (HES APC), and imaging data (HES DID, available from Apr 2012 onwards) (Supplementary Figures S1). Index of Multiple Deprivation (IMD) quintiles (a small-area measure of socioeconomic status) were assigned based on the patient’s residence postcode.

### Explanatory and outcome variables

The main outcome was the diagnostic route by which lung cancer patients were diagnosed, assigned using NCRAS’s Routes-to-Diagnosis algorithm^(17)^. We compared diagnosis via an emergency presentation (EP) with the two main referred routes: urgent referral for suspected cancer (hereafter denoted as ‘urgent’ referral, and historically known as two-week-wait referral) or GP routine referral^(18)^.

Primary explanatory variables were types of healthcare use in the 24 months before diagnosis, excluding the 30 days immediately pre-diagnosis. First, we calculated monthly mean event rates per patient. Second, we measured diagnostic window length, defined as the month when the inflection point occurred (i.e., when healthcare use started to increase from baseline).

We examined monthly rates (a month is defined as 30 days) of clinical primary care consultations (limited to one per day, excluding administrative encounters)^(19,20)^; and consultations for six selected relevant symptoms, comprising three respiratory symptoms (cough, dyspnoea, haemoptysis, assessed individually and grouped) included in referral guidelines for suspected lung cancer^(21)^, and three non-localising symptoms (appetite loss, weight loss, fatigue, assessed as a group) associated with lung cancer^(22)^. Symptoms were defined in CPRD using previously published Read V2 codelists^(23)^. Same-day repeat events of the same type were excluded. We also examined chest imaging recorded in HES DID, including chest X-ray and chest CT, using previously published National Interim Clinical Imaging Procedure (NICIP) and Systematised Nomenclature of Medicine (SNOMED) codelists^(24)^.

### Covariates

Covariates included patient factors (age at diagnosis, gender, ethnicity (identified from NCRAS, categorised as White, non-White and Unknown^(25)^), patient’s area-level (Index of Multiple Deprivation 2015), smoking status at time of diagnosis (identified using Read v2 codes in CPRD, categorised as current/ex-smokers and non-smokers^(26,27)^), Chronic Obstructive Pulmonary disease (COPD) status (identified using Readv2 and ICD-10 codes in CPRD and HES APC, categorised as no-COPD, recent-onset COPD: diagnosed within 24 months prior to lung cancer, pre-existing COPD: diagnosed more than 24 months before lung cancer diagnosis^(12,28)^), Elixhauser comorbidity score^(29,30)^ (identified using ICD-10 codes in HES APC, excluding codes for COPD and lung cancer, categorised into scores of 0,1,2, or 3+^(31)^), cancer stage at diagnosis (categorised as ‘advanced’ for stages 3–4 and non-advanced for stages 1–2)^(32)^ and morphology (non-small cell and small cell lung cancer)^(33)^. A restriction window of 12 months pre-diagnosis was applied to the Elixhauser scores^(34)^ (Supplementary Figures S2–3). Death within a year post-diagnosis defined using the death date recorded in CPRD was also described. Codelists used in this study are available online at https://github.com/martaberglund/EPlungcancer-phenotypes.git.

### Statistical analysis

We examined healthcare use across segmented pre-diagnosis periods (months 24–1, 24–12, 12–6 and 6–1 prior to diagnosis) and in line with previous studies^(6,9)^ we report on results for the 12 months pre-diagnosis where most changes occurred (Supplementary Figure S3).

We calculated crude rates as the mean number of events per patient per month. For each month, we summed total events and divided by the number of patients with follow-up. Crude rates were stratified by diagnostic route. For urgently and routinely referred groups, we also report age-standardised rates using the emergency group’s age distribution as the standard to directly allow for between-route comparisons. We calculated the cumulative monthly proportion of patients with at least one relevant event in the pre-diagnostic year.

We fitted mixed-effects Poisson models (including a random effect for patient^(35)^) to compare pre-diagnostic rates (mean monthly events per patient) by diagnostic route, using a crude model and adjusted for patient factors (age, gender, ethnicity, IMD, COPD status, smoking status and Elixhauser score). The emergency-diagnosed group was used as the reference category. Including morphology and stage did not materially affect results and therefore excluded. The month immediately pre-diagnosis was excluded from the crude rate modelling.

The Maximum Likelihood Estimation (MLE) method^(20,36)^ was used to estimate the inflection points for each healthcare use type by diagnostic route for the entire 12-month period prior to diagnosis. This method fits a series of Poisson regression models (as above), each fitting a unique inflection-point variable corresponding to a different pre-diagnosis month. The model with the month with highest log-likelihood denotes the month corresponding to the inflection point. We estimated 95% confidence intervals using bootstrapping. We refer to periods before the inflection points as background periods.

## 3. RESULTS

### Cohort description

**Figure 1.**
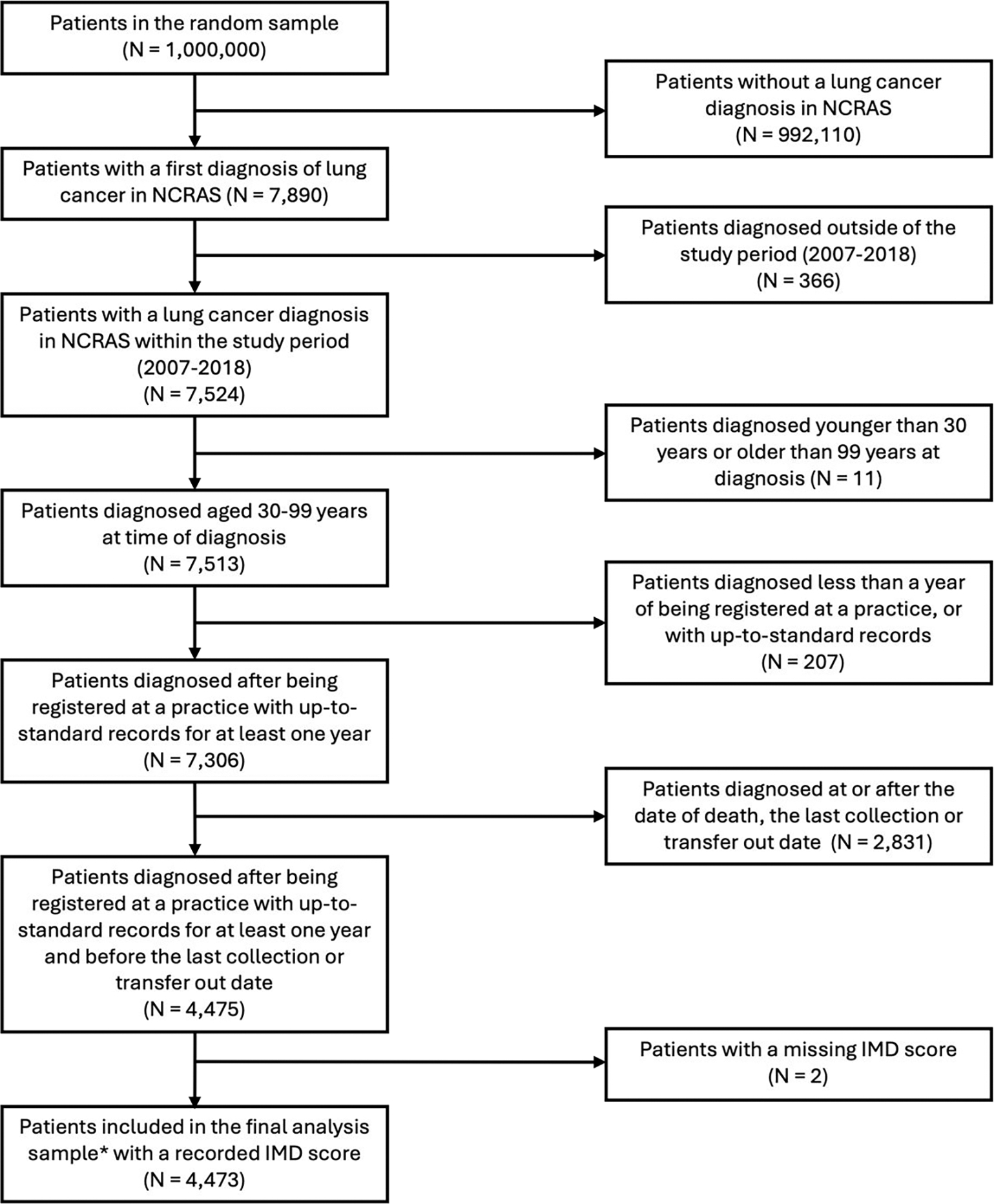
Flowchart describing the cohort selection process. *2,059 patients were diagnosed after April 1^st^ 2012, and were therefore included in analyses relating to chest imaging.

After exclusions, 4,473 patients with lung cancer were included in analysis (Figure 1): 1,491 patients (33%) were diagnosed as emergencies, 1,259 (28%) through urgent referral, 1,026 (23%) through routine referral, and 697 (16%) through other routes (Table 1). Similar proportions of patients presented to primary care before diagnosis across all routes (Table 2). Emergency-diagnosed patients were averagely older and more likely to have advanced or unknown stage at diagnosis, tumour of unspecified morphology, pre-existing or new-onset COPD, higher Elixhauser comorbidity score, and to die within a year post-diagnosis compared to referred route patients (Table 1). The regional distribution of patients is presented in Supplementary Table S1.

### Diagnostic window length

**Figure 2.**
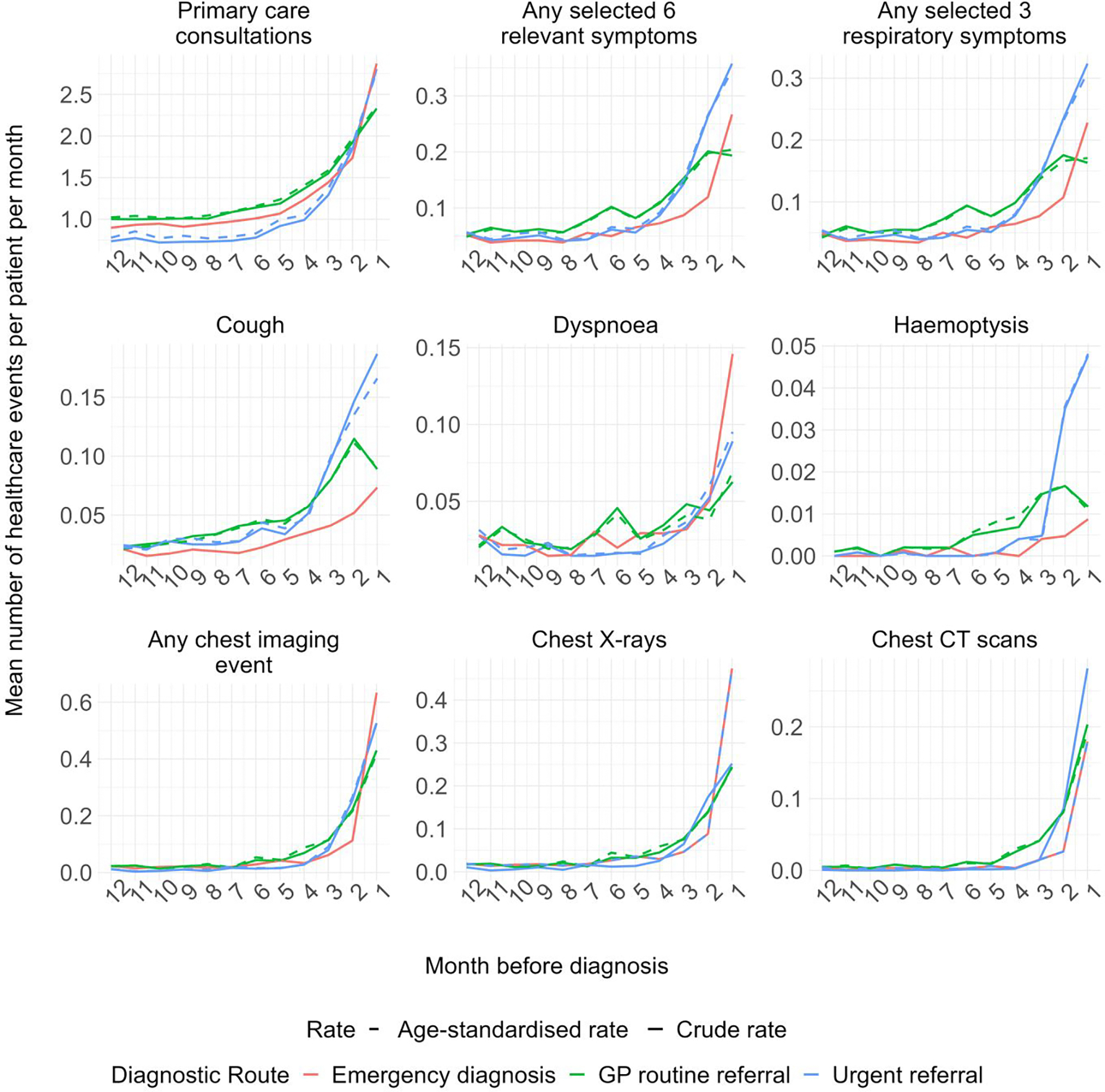
Rates of healthcare use per patient per month 12 months to 1 day before diagnosis by three main diagnostic routes, age-standardised in reference to the emergency-diagnosed group. NB: Y-axis scale differs substantially between the panels/type of healthcare event visualised. The rates were modelled using Poisson regression with data from 12 to 1 months before diagnosis adjusted for patient factors (age at diagnosis, gender, ethnicity, IMD, smoking status, COPD status and Elixhauser comorbidity score at time of diagnosis). Any selected 6 symptoms include: appetite loss, weight loss, fatigue, cough, dyspnoea and haemoptysis. Any selected 3 respiratory symptoms include: cough, dyspnoea and haemoptysis.

Diagnostic windows were present for all patient groups defined by diagnostic route and were consistently shorter or of equal length for emergency-diagnosed compared to referred route patients (Figure 2, Table 3). For example, consultations for any reason began to increase from 5 months pre-diagnosis for both emergency-diagnosed and urgently referred patients, and from 7 months for routinely referred patients. Similarly, corresponding inflection points for consultations with any of three respiratory symptoms (cough, dyspnoea or haemoptysis) were 5, 6 and 10 months, for emergency-diagnosed, routinely referred and urgently referred patients respectively.

### Overall trends in the pre-diagnostic rates of healthcare use by route

**Figure 3.**
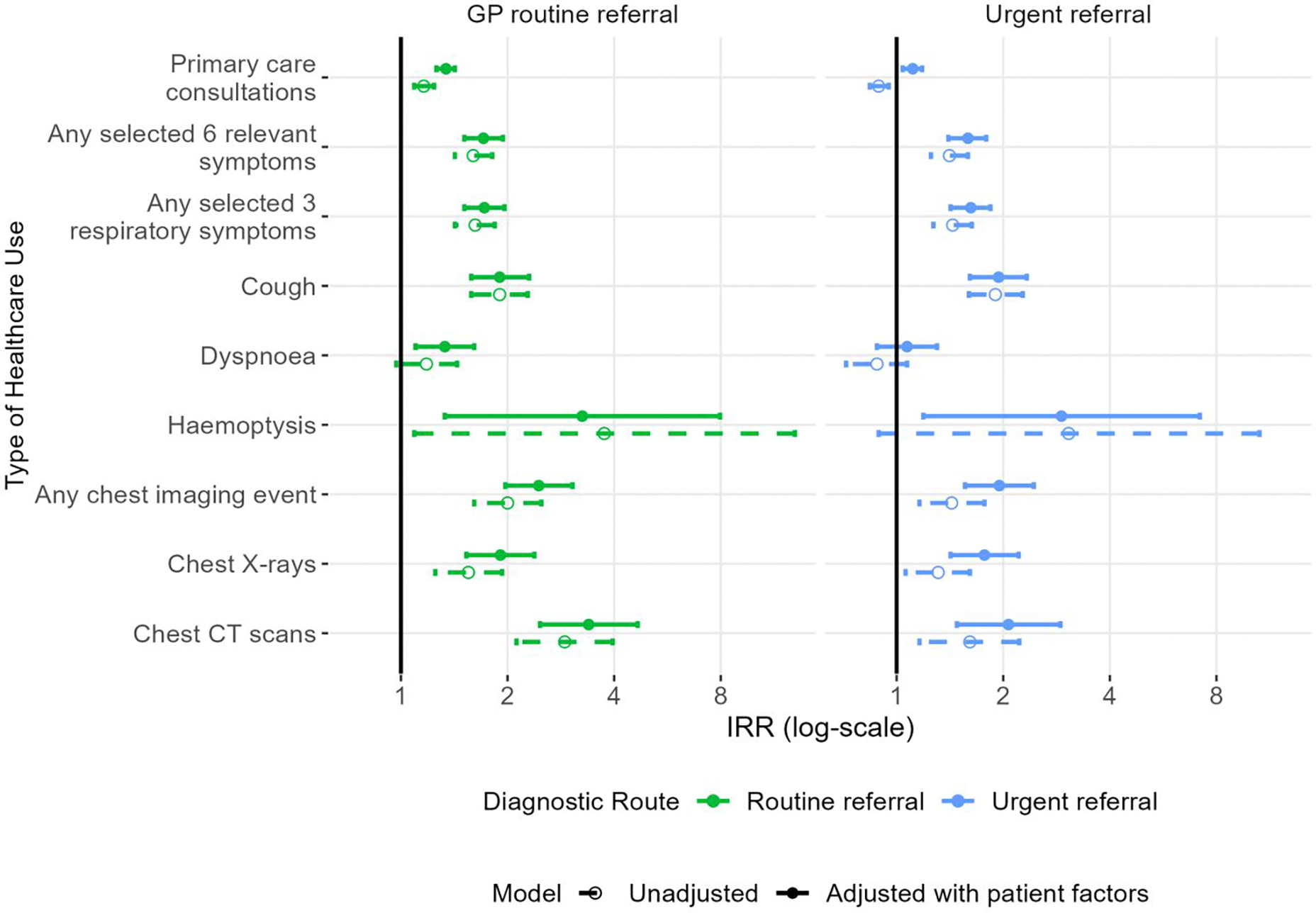
Unadjusted and adjusted Poisson model results showing the Incidence Rate Ratios (IRRs, log-scale) for healthcare use by type 12 months to 1 month before diagnosis (with emergency-diagnosed as the reference category). NB: Log transformed data are shown for the economy of visualisation; exact values are included in Table 3. Any selected 6 symptoms include: appetite loss, weight loss, fatigue, cough, dyspnoea and haemoptysis. Any selected 3 respiratory symptoms include: cough, dyspnoea and haemoptysis.

In the 12 months leading up to (excluding the month immediately before) diagnosis, emergency-diagnosed patients had consultation or imaging rates not discernible from those of patients diagnosed through referred routes (Table 2). Emergency-diagnosed patients had somewhat lower age-standardised rates of pre-diagnostic consultations or imaging in the year pre-diagnosis compared to urgently or routinely referred patients, for whom adjusted Incidence Rate Ratios (aIRRs) for both consultations and imaging were consistently higher, often significantly, than those of emergency-diagnosed patients (Supplementary Tables S2–S10; Table 3, Figure 3). Additional analyses for segmented pre-diagnostic periods (months 24–1, 24–12, 12–6, and 6–1) across all models and adjustment types are presented in Supplementary Tables S11–S16, showing broadly consistent healthcare use patterns by diagnostic route as reported in the main analysis.

### Rates of consultations for any reason and rates of chest imaging

After adjustment, pre-diagnostic consultation rates for any clinical reason were lower in emergency-diagnosed patients (aIRR for routinely referred vs emergency-diagnosed: 1.34 (95%CI: 1.26–1.42); aIRR for urgently referred vs emergency-diagnosed: 1.11 (1.04–1.18)) (Table 3, Figure 3). Similar but more pronounced differences by diagnostic route were observed for chest imaging activity (aIRR for routinely referred vs emergency-diagnosed: 2.45 (1.97–3.05); aIRR for urgently referred vs emergency-diagnosed: 1.95 (1.56–2.25)) (Table 3, Figure 3).

### Rates of consultations for selected respiratory symptoms

Emergency-diagnosed patients had lower pre-diagnostic consultation rates for any of three respiratory symptoms (cough, dyspnoea, haemoptysis) compared to routinely or urgently referred patients. This association remained significant after adjustment, for both routinely (aIRR vs emergency-diagnosed: 1.72 (1.51–1.96)); and urgently referred patients (aIRR vs emergency diagnosed: 1.62 (1.42–1.84)) (Table 3, Figure 3). The results were similar for pre-diagnostic consultation rates with any of the selected six relevant symptoms. Considering consultations for each symptom individually, adjusted pre-diagnostic consultation rates for cough were lower among emergency diagnosed patients (aIRR for routinely referred vs emergency-diagnosed: 1.90 (1.58–2.30); aIRR for urgently referred vs emergency-diagnosed 1.94 (1.61–2.33)). There was inconsistent or weak statistical evidence for variation by diagnostic route in adjusted pre-diagnostic consultation rates for haemoptysis or dyspnoea (Table 3, Figure 3).

## 4. DISCUSSION

### Summary

Diagnostic windows of appreciable length were observed in patients with lung cancer, defined by both consultations and imaging activity. In the year pre-diagnosis, emergency-diagnosed patients had lower adjusted rates of consultations and chest imaging than those diagnosed via urgent or routine referrals. Improving referral pathways and post-imaging follow-up may support earlier diagnosis across routes, though fewer pre-diagnostic contacts among emergency-diagnosed patients limit opportunities to intervene compared with referred route patients. Surprisingly, as denoted by diagnostic windows of similar length, this potential did not vary substantially between diagnostic routes.

### Strengths and limitations

We analysed a representative primary care cohort with linked healthcare data but were limited to variables recorded in electronic patient records. This leaves scope for residual confounding from unmeasured variables. Specifically, our findings are susceptible to ‘confounding by indication’, whereby unaccounted-for case-mix differences may influence both diagnostic route and diagnostic windows. The analysis relied on coded rather than free-text data^(37)^, and imaging results were unavailable. Our findings are limited to those diagnosed with lung cancer, as we aimed to compare diagnostic windows by route within this group, we did not examine healthcare use patterns in a reference population. Although more recent datasets, such as CPRD Aurum, could be used in future research, cancer registry linkage is currently available only up to 2021. Therefore, major changes in the findings are unlikely, apart from potential effects related to the COVID-19 pandemic. Incorporating additional data such as referral source, prescriptions, and test results could improve understanding of pre-diagnostic healthcare use and missed diagnostic opportunities.

The MLE method used to estimate the inflection points assumes a linear trend in healthcare use post-inflection point. Our model assumed linear relationships between the covariates and healthcare use, and did not include possible interaction terms between variables^(38)^. We used a case-only design without population controls, as our comparisons related to patients with lung cancer diagnosed through different routes^(38)^.

Emergency-diagnosed patients have higher one-year mortality even after adjusting for stage and morphology^(39,40)^. While comparing treatment by diagnostic route may clarify factors leading to poorer outcomes, such analyses were beyond our scope.

### Comparisons with existing literature

Consistent with prior studies (measuring primary care consultations, prescriptions, blood tests, and chest X-rays), we observed increased healthcare use beginning 4–6 months pre-diagnosis^(11,13,40)^. However, our study additionally profiles diagnostic windows and related healthcare activity by diagnostic route for the first time in patients with lung cancer. A similar approach has been recently reported for diagnostic windows for patients with haematological cancers diagnosed through different routes^(15)^.

The notion that emergency-diagnosed patients consult infrequently in primary care is not supported by our findings, showing similar background consultation patterns across routes^(41)^. Furthermore, the finding of lower healthcare use during the diagnostic window among emergency-diagnosed patients aligns with studies on other cancer sites^(8,9,14,15,31)^.

Diagnostic window lengths for emergency-diagnosed patients were similar or shorter than for referred route patients. Patients with lung cancer often present with non-specific symptoms such as cough, dyspnoea, respiratory infections, and chest pain^(42–44)^. Consistent with previous studies, we found that haemoptysis, the respiratory symptom with higher positive predictive value for lung cancer, was relatively rare^(45,46)^. These findings concord with those observed for colorectal cancer, where similar diagnostic window length and fewer alarm symptoms (such as rectal bleeding) were reported in emergency-diagnosed patients^(8,9)^.

Fewer chest imaging events were observed among emergency-diagnosed patients. Patients with higher primary care engagement and guideline-concordant care are more likely to receive timely imaging^(11,24,47)^.

Tumour stage and morphology are associated with emergency diagnosis^(14,39)^, but adjusting for them did not materially alter the findings regarding event rates.

### Implications for research and practice

Emergency-diagnosed patients consulted less with the selected symptoms and underwent fewer pre-diagnostic imaging investigations than those diagnosed through urgent or routine referrals. This may reflect genuine differences in tumour presentation across diagnostic routes, or patient-related factors such as health literacy (e.g., poorer recognition of significant symptoms) and delayed help-seeking despite symptom experience^(48–51)^. Health literacy and intervals from symptom onset to help-seeking are not captured in routine health record data, yet are likely to contribute to the observed differences and should be explored in future research.

This study provides population-level evidence on diagnostic routes for lung cancer and highlights potential for earlier diagnosis. Substantial diagnostic windows across all routes suggest the need for generic improvements, such as better risk assessment of non-specific symptoms and greater imaging use. For emergency-diagnosed patients, fewer symptomatic consultations mean fewer primary care opportunities to act on signs that might have prompted non-emergency referral. Further work should examine where specific actions, particularly for chest imaging results, could have expedited diagnosis.

As around a fifth of lung cancers are not detectable via chest X-ray at the time of symptomatic presentation^(47)^, the observed diagnostic windows defined by chest imaging are likely to reflect false negative investigations, underscoring the need for proactive follow-up (‘safety netting’) and wider chest CT access (for examples, as a follow-up chest imaging test, after initial negative chest X-ray when symptoms and diagnostic suspicion prevail)^(50)^. Expanding GP access to chest CTs, which have greater sensitivity than chest X-rays, could improve early lung cancer detection and reduce emergency diagnoses^(51)^. Similar findings in urological cancers show that use of less-sensitive imaging modalities can hinder timely diagnosis^(52)^.

Although rare, delays in acting on abnormal chest X-ray findings may represent missed diagnostic opportunities; future research should aim to quantify whether and how often such safety incidents might be occurring^(40)^. Evaluating adherence to referral/imaging guidelines and detailed event analysis may identify missed diagnostic opportunities and improve care across diagnostic routes^(24,53)^. Such studies can inform interventions targeting potentially avoidable emergency diagnoses.

Lung cancer screening could reduce emergency diagnoses, as shown in colorectal cancer^(53–56)^. In our cohort, 49% of patients (including 38% of emergency-diagnosed patients) were aged 55–74, within the eligible screening range depending on smoking history. However, participation in screening is lower among socioeconomically deprived groups, who are more likely to be diagnosed via an emergency route^(57)^. Thus, while screening may reduce emergency presentations, equitable uptake is essential to avoid widening inequalities in lung cancer diagnosis.

### Conclusion

Comparing pre-diagnostic healthcare use between emergency-diagnosed and referred lung cancer patients revealed no clear aetiological targets to reduce emergency diagnosis. Instead, our findings suggest potential to expedite diagnosis across all groups. In particular, through improved management of non-specific symptoms, timely follow-up of imaging, and greater access to chest CTs for suspected cancer.

## Supplementary Material

Supplementary Material

## Figures and Tables

**Table 1.** Comparison of characteristics of the studied lung cancer patients by diagnostic route (n=4,473)

Variable	Emergency diagnosis, N=1491 (33%)	Routine referral, N=1026 (23%)	Urgent referral, N=1259 (28%)	Other*, N=697 (16%)	p-value^†^

**Age at diagnosis^‡^**					<0.001
	75 (11), 76 (68, 84), 32 – 99	72 (10), 73 (65, 80), 39–96	71 (10), 71 (63, 78), 36–97	72 (11), 72 (65, 79), 38–98	
**Age at diagnosis**					<0.001
30–64	258 (17%)	235 (23%)	343 (27%)	172 (25%)	
65–74	391 (26%)	360 (35%)	441 (35%)	231 (33%)	
75–84	508 (34%)	334 (33%)	363 (29%)	220 (32%)	
85–99	334 (22%)	97 (9.5%)	112 (8.9%)	74 (11%)	
**Stage of cancer at diagnosis**					<0.001
Advanced	785 (53%)	473 (46%)	695 (55%)	289 (41%)	
Not advanced	106 (7.1%)	210 (20%)	214 (17%)	142 (20%)	
Unknown	600 (40%)	343 (33%)	350 (28%)	266 (38%)	
**Gender**					0.130
Female	722 (48%)	470 (46%)	558 (44%)	337 (48%)	
Male	769 (52%)	556 (54%)	701 (56%)	360 (52%)	
**Ethnicity**					<0.001
White	1,388 (93%)	962 (94%)	1,198 (95%)	618 (89%)	
Non-White	81 (5.4%)	45 (4.4%)	48 (3.8%)	45 (6.5%)	
Unknown	22 (1.5%)	19 (1.9%)	13 (1.0%)	34 (4.9%)	
**IMD**					0.500
1-Least deprived	239 (16%)	191 (19%)	200 (16%)	134 (19%)	
2	271 (18%)	205 (20%)	239 (19%)	137 (20%)	
3	319 (21%)	209 (20%)	267 (21%)	148 (21%)	
4	325 (22%)	215 (21%)	284 (23%)	144 (21%)	
5 - Most deprived	337 (23%)	206 (20%)	269 (21%)	134 (19%)	
**Smoker status**					<0.001
Smoker	1,315 (88%)	920 (90%)	1,164 (92%)	605 (87%)	
Non-smoker	176 (12%)	106 (10%)	95 (7.5%)	92 (13%)	
**COPD status**					0.001
New-onset COPD (during months -24–0)	238 (16%)	148 (14%)	150 (12%)	84 (12%)	
Pre-existing COPD (at - 24 months or earlier)	356 (24%)	222 (22%)	252 (20%)	152 (22%)	
No COPD	897 (60%)	656 (64%)	857 (68%)	461 (66%)	
**Elixhauser comorbidity score**					<0.001
0	144 (9.7%)	259 (25%)	439 (35%)	174 (25%)	
1	161 (11%)	184 (18%)	301 (24%)	121 (17%)	
2	257 (17%)	195 (19%)	202 (16%)	116 (17%)	
3+	929 (62%)	388 (38%)	317 (25%)	286 (41%)	
**Survival at 1 year from diagnosis**					<0.001
Died	1,176 (79%)	528 (51%)	634 (50%)	351 (50%)	
Alive	315 (21%)	498 (49%)	625 (50%)	346 (50%)	
**Morphology** ^§^					<0.001
SCLC	133 (8.9%)	85 (8.3%)	170 (14%)	59 (8.5%)	
NSCLC	684 (46%)	718 (70%)	915 (73%)	462 (66%)	
Unspecified	674 (45%)	223 (22%)	174 (14%)	176 (25%)	

* Other routes include: Inpatient Elective, Other Managed Pathway, DCO (Diagnosis by Death Certificate Only), and Unknown^(17)^

† One way ANOVA for continuous age at diagnosis; otherwise Pearson’s chi-squared test

‡ Mean (SD), Median (IQR), Range; n (%)

§ SCLC: Small Cell Lung Cancer: NSCLC: Non-Small Cell Lung Cancer

**Table 2.** Summary of the proportion of patients and mean events per patient per month of the included healthcare use measures 12 to 1 month pre-diagnosis by diagnostic route.

	Emergency diagnosis, N=1491 (33%)	Routine referral, N=1026 (23%)	Urgent referral, N=1259 (28%)	Other*, N=697 (16%)	p-value^†^

**Proportion of patients with at least one event, % (n)**					

*Consultations*					
Any	1,473 (98.8%)	1,023 (99.7%)	1,252 (99.4%)	684 (98.1%)	<0.001
With any selected 6 relevant symptoms^‡^	859 (57.6%)	669 (65.2%)	891 (70.8%)	400 (57.4%)	<0.001
With any selected 3 respiratory symptoms^§^	791 (53.1%)	610 (59.5%)	833 (66.2%)	355 (50.9%)	<0.001
With cough	472 (31.7%)	433 (42.2%)	606 (48.1%)	223 (32.0%)	<0.001
With dyspnoea	533 (35.8%)	327 (31.9%)	367 (29.2%)	210 (30.1%)	<0.001
With haemoptysis	35 (2.4%)	71 (6.9%)	104 (8.3%)	25 (3.6%)	<0.001

*Imaging events^||^*					
Any chest imaging	604 (88.8%)	449 (86.3%)	552 (92.7%)	244 (83.8%)	<0.001
Chest X-ray	577 (84.9%)	399 (76.7%)	513 (86.2%)	224 (77%)	<0.001
Chest CT	341 (50.1%)	353 (67.9%)	465 (78.2%)	179 (61.5%)	<0.001

**Mean events per patient per month (95% CI)** ^¶^					

*Consultations*					
Any	1.10 (1.02–1.17)	1.21 (1.11–1.3)	0.93 (0.86–1.00)	0.99 (0.89–1.09)	
With any selected 6 relevant symptoms^‡^	0.06 (0.05–0.07)	0.09 (0.07–0.11)	0.08 (0.06–0.10)	0.07 (0.05–0.09)	
With any selected 3 respiratory symptoms^§^	0.05 (0.04–0.07)	0.05 (0.04–0.07)	0.05 (0.04–0.07)	0.06 (0.04–0.08)	
With cough	0.03 (0.02–0.03)	0.05 (0.03–0.06)	0.05 (0.04–0.06)	0.04 (0.02–0.05)	
With dyspnoea	0.03 (0.02–0.04)	0.03 (0.02–0.04)	0.02 (0.01–0.03)	0.03 (0.01–0.04)	
With haemoptysis	0.0 (0.0–0.0)	0.01 (0–0.01)	0.0 (0.0–0.01)	0.0 (0.0–0.0)	

*Imaging events^||^*					
Any chest imaging	0.04 (0.02–0.05)	0.06 (0.04–0.07)	0.04 (0.03–0.05)	0.05 (0.03–0.06)	
Chest X-ray	0.03 (0.02–0.04)	0.04 (0.03–0.05)	0.03 (0.02–0.04)	0.03 (0.02–0.05)	
Chest CT	0.01 (0–0.01)	0.02 (0.01–0.03)	0.01 (0.01–0.01)	0.01 (0.01–0.02)	

* Other routes include: Inpatient Elective, Other Managed Pathway, DCO (Diagnosis by Death Certificate Only), and Unknown^(17)^

† One-way ANOVA

‡ Symptoms include appetite loss, weight loss, fatigue, cough, dyspnoea and haemoptysis

§ Symptoms include cough, dyspnoea and haemoptysis

|| Proportions calculated out of all patients diagnosed after April 1^st^ 2012 (Emergency presentation: N = 680; Routine referral: N = 493; Urgent referral: N = 595; Other: N = 291)

¶ Excluding the month immediately prior to diagnosis

**Table 3.** Summary table of the measures of healthcare use by diagnostic route including the pre-diagnostic rates and inflection points within 12 to 1 months before lung cancer diagnosis by diagnostic route compared to emergency diagnosed patients.

	Adjusted IRR (95% CI)			Inflection point estimate (95% CI)		
Type of healthcare use	*Emergency - diagnosed*	Routinely referred	Urgently referred	*Emergency - diagnosed*	Routinely referred	Urgently referred
Primary care consultations	*1 (Ref)*	↑ 1.34 (1.26–1.43)	↑ 1.11 (1.04–1.18)	*5 (4.99 – 5.01)*	← 7 (6.96 – 7.04)	↔ 5 (4.99 – 5.01)
Consultations with any selected 6 relevant symptoms*	*1 (Ref)*	↑ 1.71 (1.51–1.94)	↑ 1.59 (1.40–1.79)	*5 (4.95 – 5.05)*	← 9 (8.93– 9.07)	← 6 (5.97 – 6.03
Consultations with any selected 3 respiratory symptoms†	*1 (Ref)*	↑ 1.72 (1.51–1.96)	↑ 1.62 (1.42–1.84)	*5 (4.95 – 505)*	← 10 (9.93 – 10.07)	← 6 (5.97 – 6.03)
Consultations with cough	*1 (Ref)*	↑ 1.90 (1.58–2.30)	↑ 1.94 (1.61–2.33)	*7 (6.94 – 7.06*)	← 10 (9.93 –10.07)	↔ 7 (6.96 – 7.04)
Consultations with dyspnoea	*1 (Ref)*	↑ 1.33 (1.11–1.61)	↑ 1.07** (0.88–1.30)	*3 (2.96 – 3.04)*	← 9 (8.88 – 9.12)	← 5 (4.96 – 5.04)
Consultations with haemoptysis	*1 (Ref)*	↑ 3.25 (1.33–7.96)	↑ 2.92 (1.19–7.18)	*5 (4.88 – 5.12)*	← 10 (9.93 – 10.07)	← 7 (6.94 – 7.06)
Any chest imaging events	*1 (Ref)*	↑ 2.45 (1.97–3.05)	↑ 1.95 (1.56–2.25)	*4 (3.98 – 4.02)*	←7 (6.97 – 7.03)	← 6 (5.98 – 6.02)
Chest X-rays	*1 (Ref)*	↑ 1.91 (1.53–2.38)	↑ 1.77 (1.42–2.21)	*4 (3.97 – 4.03)*	← 7 (6.96 – 7.04)	← 6 (5.96 – 6.04)
Chest CT	*1 (Ref)*	↑ 3.39 (2.47–4.66)	↑ 2.07 (1.48–2.90)	*4 (3.99 – 4.01)*	← 6 (5.96 – 6.04)	← 6 (5.97 – 6.03)

* Symptoms include appetite loss, weight loss, fatigue, cough, dyspnoea and haemoptysis

† Symptoms include cough, dyspnoea and haemoptysis

The aIRR values show the Poisson modelling results using data from 12 to 1 months before diagnosis adjusted for patient factors (age at diagnosis, gender, ethnicity, IMD, smoking status, COPD status and Elixhauser comorbidity score at time of diagnosis). The upwards-facing arrows represent significant positive association (p < 0.05), results marked with an ** represent results with weak evidence (p > 0.05). A difference in the inflection points ≤2 months is considered equal (↔) compared to EPs, inflection points occurring >2 months before those for EPs are represented with a left-facing arrow (←).

## Data Availability

Data cannot be shared by the researchers, but access to CPRD data is available subject to protocol approval via CPRD’s Research Data Governance (RDG) Process, see https://cprd.com/data-access for further details.

## How this fits in

It has been postulated that emergency diagnoses of cancer (which occurs frequently and confers a poorer prognosis) may relate to suboptimal diagnostic management in primary care, but evidence to support or refute this hypothesis is sparse.

We found that emergency-diagnosed patients with lung cancer were less likely to present with relevant respiratory symptoms and had fewer chest imaging investigations before diagnosis compared to patients diagnosed via referred routes, indicating an important role of disease factors in emergency diagnosis.

We also found that the rate of pre-diagnostic healthcare activity increased at the same time or closer to diagnosis for emergency-diagnosed patients compared to those diagnosed through referred routes, suggesting a similar potential for earlier diagnosis between all diagnostic routes.

Improved imaging follow-up, greater access and use of CT imaging, and greater use of safety netting in primary care could help diagnose lung cancer earlier across all diagnostic routes.
