# Supplementary material for: Pre-diagnostic primary care consultations and imaging in emergency-diagnosed vs referred lung cancer patients
Source: Br J Gen Pract. Author manuscript; Available in PMC 2026 May 4. (PMC13060713; doi:10.3399/BJGP.2025.0369)
Supplement: Supplementary Material [file NIHMS2148516-supplement-Supplementary_Material.docx]

**SUPPLEMENTARY DATA**

**Table of Contents**

**Supplementary Figure S1.** Sensitivity analysis of the mean number of chest imaging events per patient per month leading up to diagnosis, for the entire period of available HES DID data for the cohort (2012-2018).

**Supplementary Figure S2.** Sensitivity analysis of the proportion of patients with a new COPD diagnosis per month leading up to diagnosis, in the 24 months before diagnosis for the full cohort.

**Supplementary Figure S3.** Sensitivity analysis of the mean number of Elixhauser scores per patient month leading up to diagnosis (top panel) and count of comorbidities per patient (bottom panel) in the 24 months before diagnosis for the full cohort.

**Supplementary Figure S4.** Sensitivity analysis of the mean number of primary care consultations per patient per month 10 years before diagnosis (2008-2018).

**Supplementary Table S1.** Regional distribution of patients by diagnostic route.

**Supplementary Table S2.** Summary statistics for primary care consultations the 12 months before diagnosis by diagnostic route.

**Supplementary Table S3.** Summary statistics for consultations with 6 relevant symptoms in the 12 months before diagnosis by diagnostic route.

**Supplementary Table S4.** Summary statistics for consultations with 3 main respiratory symptoms in the 12 months before diagnosis by diagnostic route.

**Supplementary Table S5.** Summary statistics for consultations with cough in the 12 months before diagnosis by diagnostic route.

**Supplementary Table S6.** Summary statistics for consultations with dyspnoea in the 12 months before diagnosis by diagnostic route.

**Supplementary Table S7**. Summary statistics for consultations with haemoptysis in the 12 months before diagnosis by diagnostic route.

**Supplementary Table S8.** Summary statistics for any chest imaging events in the 12 months before diagnosis by diagnostic route.

**Supplementary Table S9.** Summary statistics for chest X-rays in the 12 months before diagnosis by diagnostic route.

**Supplementary Table S10**. Summary statistics for chest CT scans in the 12 months before diagnosis by diagnostic route.

**Supplementary Table S11**. Modelled incidence rate ratios (IRRs) for pre-diagnostic healthcare use*, by diagnostic route, with and without adjustment for socio-demographic factors ( -12 to -2 months).

**Supplementary Table S12**. Modelled incidence rate ratios (IRRs) for pre-diagnostic healthcare use, by diagnostic route, with and without adjustment for socio-demographic and tumour factors (-24 to -2 months).

**Supplementary Table S13**. Modelled incidence rate ratios (IRRs) for pre-diagnostic healthcare use, by diagnostic route, with and without adjustment for socio-demographic and tumour factors (-24 to -6 months).

**Supplementary Table S14**. Modelled incidence rate ratios (IRRs) for pre-diagnostic healthcare use, by diagnostic route, with and without adjustment for socio-demographic and tumour factors (-12 to -6 months). (NOTE: data on patients with haemoptysis was insufficient for analysis in this segmentation.)

**Supplementary Table S15**. Modelled incidence rate ratios (IRRs) for pre-diagnostic healthcare use, by diagnostic route, with and without adjustment for socio-demographic and tumour factors (-6 to -2 months).

**Supplementary Table S16**. Modelled incidence rate ratios (IRRs) for pre-diagnostic symptomatic consultations excluding patients with haemoptysis, by diagnostic route, with and without adjustment for socio-demographic and tumour factors (-12 to -2 months).

**Supplementary Figure S1.** Sensitivity analysis of the mean number of chest imaging events per patient per month leading up to diagnosis, for the entire period of available HES DID data for the cohort (2012-2018).


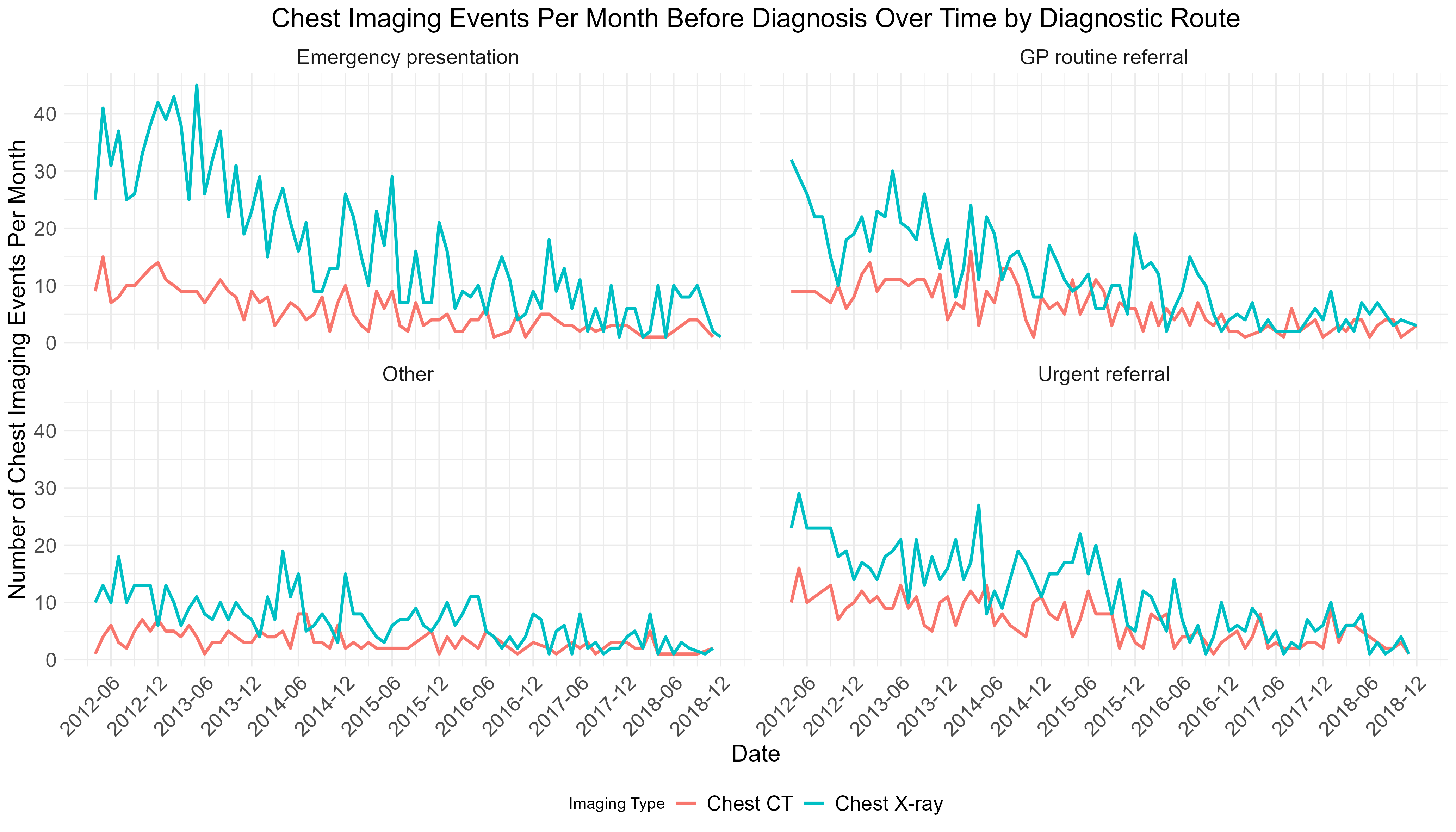


**Supplementary Figure S2.** Sensitivity analysis of the proportion of patients with a new COPD diagnosis per month leading up to diagnosis, in the 24 months before diagnosis for the full cohort.
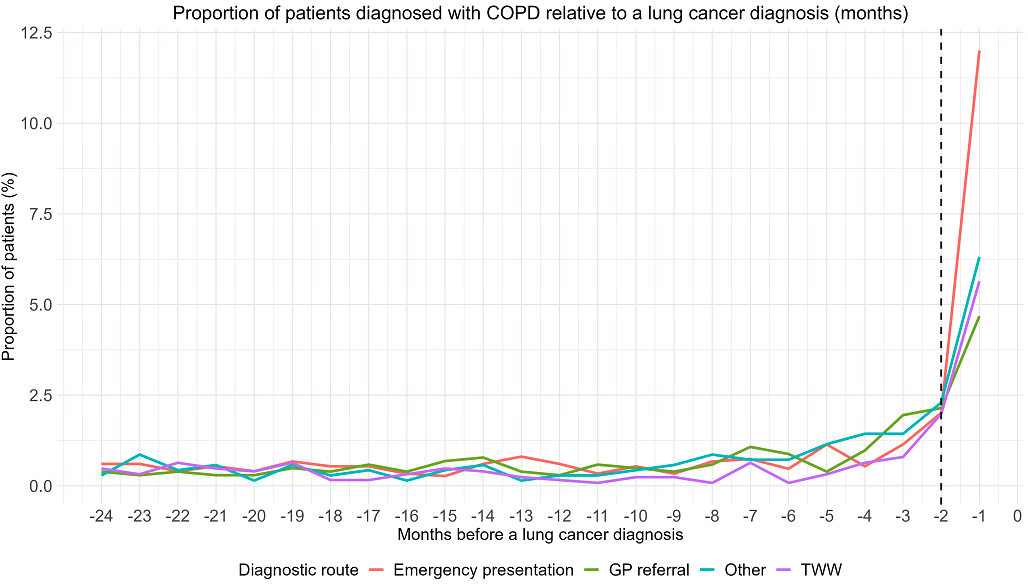


**Supplementary Figure S3.** Sensitivity analysis of the mean number of Elixhauser scores per patient month leading up to diagnosis (top panel) and count of comorbidities per patient (bottom panel) in the 24 months before diagnosis for the full cohort.


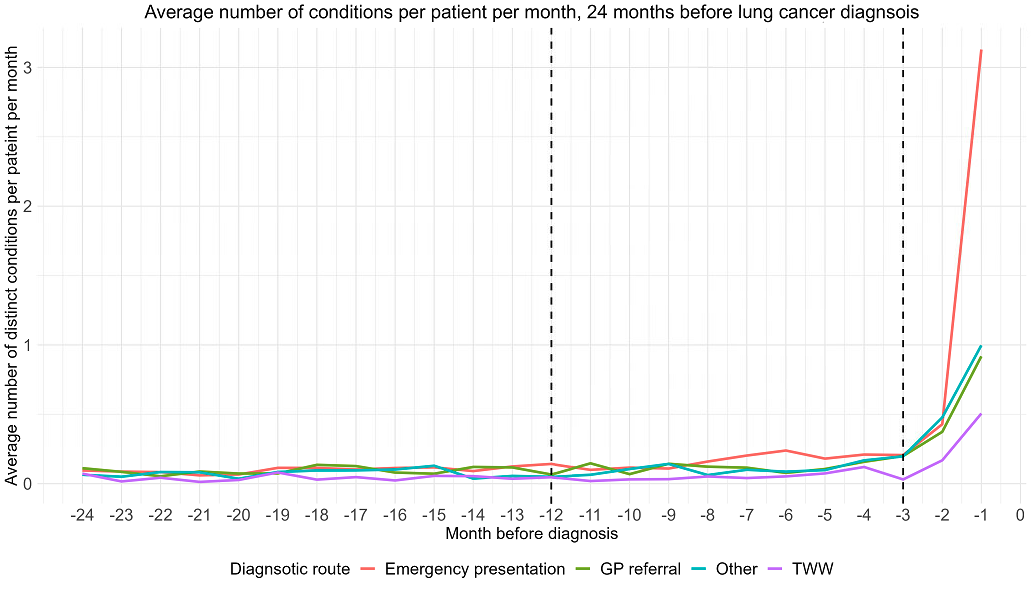


**
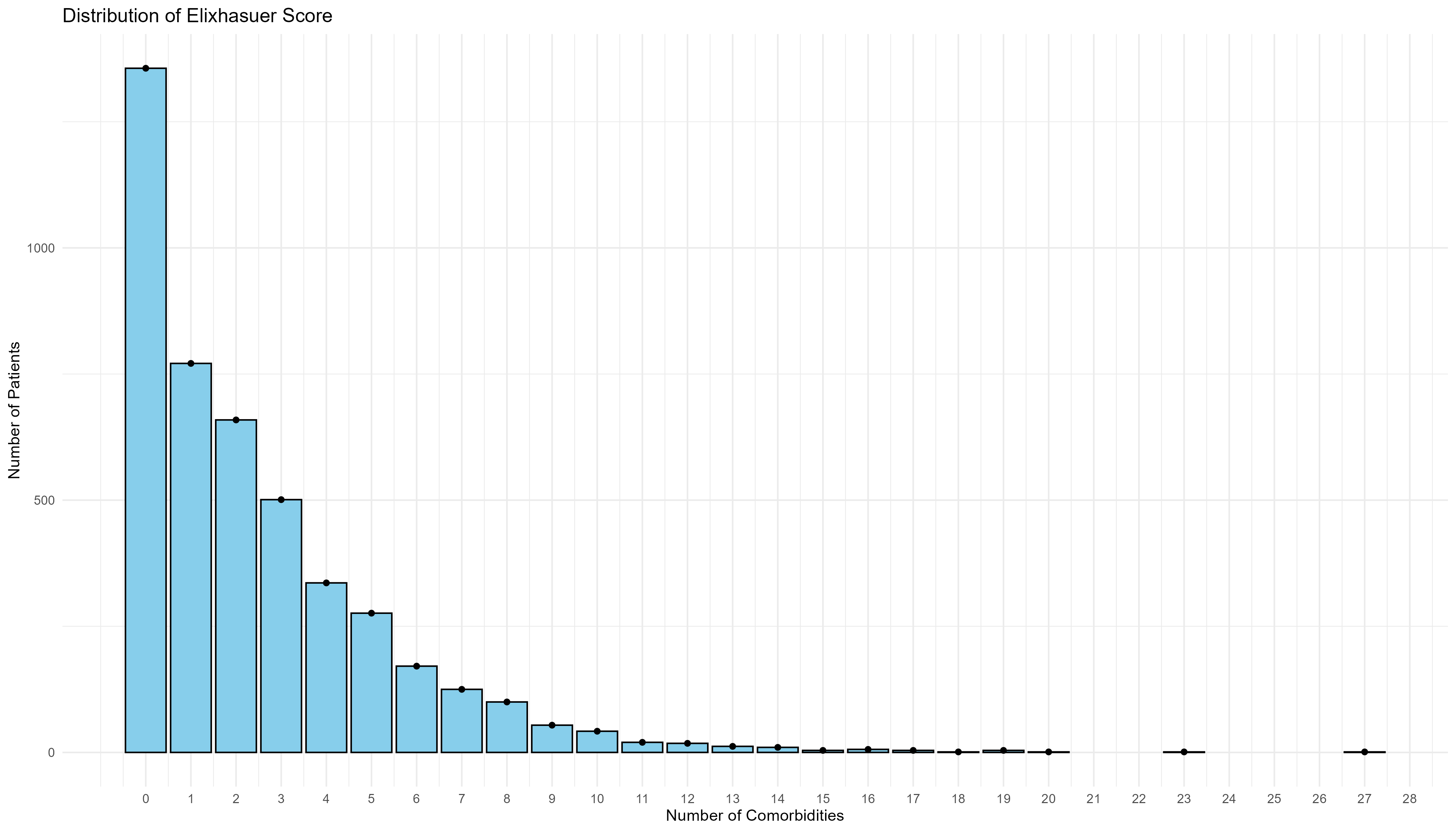
**

**Supplementary Figure S4.** Sensitivity analysis of the mean number of primary care consultations per patient per month 10 years before diagnosis (2008-2018).


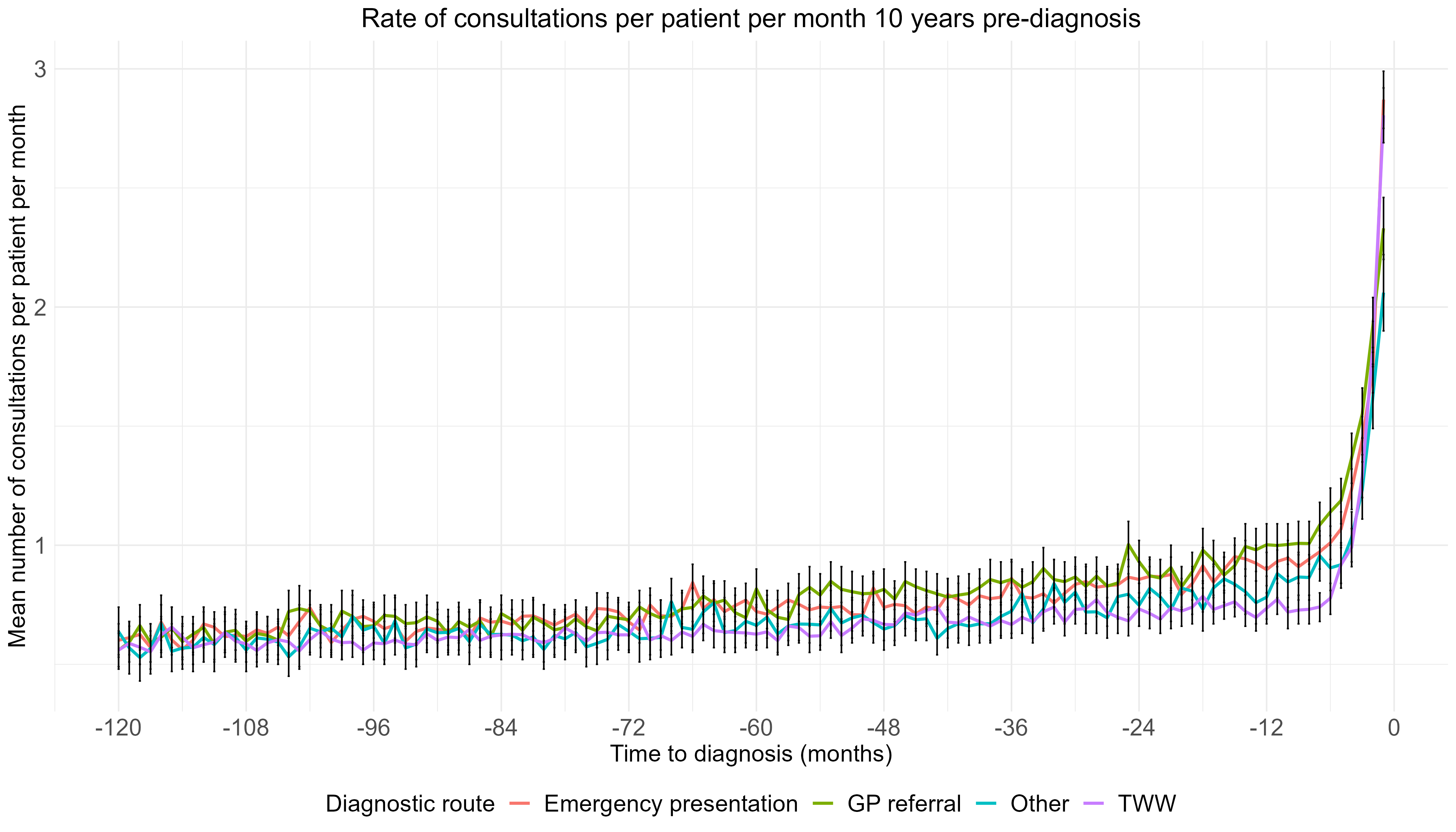


**Supplementary Table S1.** Regional distribution of patients by diagnostic route.

| **Region** | **Emergency diagnosis (N=1,491)** | **Routine referral (N=1,026)** | **Urgent referral (N=1,259)** | **Other**  **(N=697)** | **Total**  **(N=4,473)** |
| --- | --- | --- | --- | --- | --- |
|  | n (%) | n (%) | n (%) | n (%) | n (%) |
| East Midlands | 25 (1.68) | 14 (1.36) | 26 (2.07) | 20 (2.87) | 85 (1.9) |
| East of England | 116 (7.78) | 77 (7.50) | 84 (6.67) | 70 (10.04) | 347 (7.8) |
| London | 153 (10.26) | 109 (10.62) | 88 (6.99) | 83 (11.91) | 433 (9.7) |
| North East | 50 (3.35) | 22 (2.14) | 50 (3.97) | 20 (2.87) | 142 (3.2) |
| North West | 333 (22.33) | 192 (18.71) | 300 (23.83) | 129 (18.51) | 954 (21.3) |
| South Central | 188 (12.61) | 156 (15.20) | 134 (10.64) | 82 (11.76) | 560 (12.5) |
| South East Coast | 213 (14.29) | 166 (16.18) | 196 (15.57) | 100 (14.35) | 675 (15.1) |
| South West | 186 (12.47) | 113 (11.01) | 183 (14.54) | 73 (10.47) | 555 (12.4) |
| West Midlands | 175 (11.74) | 134 (13.06) | 152 (12.07) | 101 (14.49) | 562 (12.6) |
| Yorkshire & The Humber | 52 (3.49) | 43 (4.19) | 46 (3.65) | 19 (2.73) | 160 (3.6) |

**Supplementary Table S2.**Summary statistics for primary care consultations the 12 months before diagnosis by diagnostic route.

Pre-diagnostic Rates of Primary Care Consultations by Diagnostic Route

| **Month before diagnosis** | **Emergency presentation (N=1,491)** | | | **GP routine referral (N=1,026)** | | | **Urgent referral (N=1,259)** | | | **Other (N=697)** | | |
| --- | --- | --- | --- | --- | --- | --- | --- | --- | --- | --- | --- | --- |
| **Time** | **Mean consultations (95%CI)*** | **Age-standardised mean (95%CI)**** | **Cumulative proportion of patients***** | **Mean consultations (95%CI)*** | **Age-standardised mean (95%CI)**** | **Cumulative proportion of patients***** | **Mean consultations (95%CI)*** | **Age-standardised mean (95%CI)**** | **Cumulative proportion of patients***** | **Mean consultations (95%CI)*** | **Age-standardised mean (95%CI)**** | **Cumulative proportion of patients***** |
| -12 | 0.9 (0.83-0.97) | 0.9 (0.85-0.95) | 93.19% (1341) | 1 (0.91-1.09) | 1.02 (0.95-1.1) | 95.05% (941) | 0.74 (0.67-0.8) | 0.78 (0.72-0.84) | 92.84% (1141) | 0.78 (0.69-0.87) | 0.78 (0.71-0.86) | 92.86% (624) |
| -11 | 0.93 (0.86-1) | 0.93 (0.88-0.98) | 93.56% (1350) | 1 (0.91-1.09) | 1.04 (0.97-1.11) | 95.86% (950) | 0.77 (0.71-0.84) | 0.85 (0.79-0.92) | 93.43% (1152) | 0.88 (0.78-0.98) | 0.9 (0.82-0.98) | 93.91% (632) |
| -10 | 0.95 (0.87-1.02) | 0.95 (0.9-1) | 94.07% (1364) | 1 (0.92-1.09) | 1.02 (0.95-1.09) | 95.89% (957) | 0.72 (0.65-0.78) | 0.77 (0.71-0.83) | 93.6% (1155) | 0.85 (0.74-0.95) | 0.86 (0.79-0.94) | 94.37% (637) |
| -9 | 0.91 (0.84-0.97) | 0.91 (0.86-0.96) | 94.43% (1374) | 1.01 (0.92-1.1) | 1.01 (0.94-1.08) | 96.02% (966) | 0.73 (0.67-0.79) | 0.8 (0.74-0.86) | 93.86% (1162) | 0.87 (0.77-0.96) | 0.89 (0.81-0.97) | 94.54% (641) |
| -8 | 0.94 (0.87-1.01) | 0.94 (0.89-0.99) | 94.59% (1382) | 1.01 (0.92-1.1) | 1.04 (0.97-1.12) | 96.32% (969) | 0.73 (0.67-0.79) | 0.77 (0.71-0.83) | 94.35% (1169) | 0.86 (0.77-0.96) | 0.87 (0.79-0.95) | 94.99% (645) |
| -7 | 0.97 (0.9-1.04) | 0.97 (0.92-1.02) | 94.88% (1391) | 1.09 (0.99-1.18) | 1.09 (1.02-1.16) | 96.73% (976) | 0.74 (0.68-0.8) | 0.79 (0.73-0.85) | 94.69% (1176) | 0.96 (0.85-1.06) | 0.98 (0.9-1.06) | 94.73% (647) |
| -6 | 1.01 (0.94-1.08) | 1.01 (0.96-1.06) | 95.1% (1397) | 1.14 (1.04-1.24) | 1.16 (1.08-1.23) | 97.04% (983) | 0.78 (0.71-0.84) | 0.83 (0.77-0.89) | 94.94% (1183) | 0.9 (0.81-1) | 0.93 (0.85-1.01) | 95.33% (653) |
| -5 | 1.07 (1-1.14) | 1.07 (1.02-1.12) | 95.05% (1401) | 1.19 (1.09-1.28) | 1.24 (1.16-1.32) | 97.24% (988) | 0.92 (0.84-0.99) | 1 (0.93-1.06) | 95.44% (1192) | 0.92 (0.82-1.01) | 0.94 (0.86-1.03) | 95.37% (659) |
| -4 | 1.24 (1.15-1.32) | 1.23 (1.18-1.29) | 95.47% (1413) | 1.37 (1.26-1.47) | 1.41 (1.32-1.49) | 97.25% (991) | 0.99 (0.91-1.07) | 1.05 (0.98-1.12) | 95.85% (1200) | 1.03 (0.93-1.14) | 1.08 (0.99-1.17) | 96.24% (666) |
| -3 | 1.44 (1.35-1.54) | 1.44 (1.38-1.5) | 96.16% (1426) | 1.55 (1.45-1.66) | 1.59 (1.5-1.68) | 98.33% (1003) | 1.29 (1.2-1.38) | 1.37 (1.29-1.45) | 96.57% (1212) | 1.23 (1.11-1.35) | 1.25 (1.16-1.35) | 96.69% (671) |
| -2 | 1.74 (1.64-1.83) | 1.74 (1.67-1.8) | 97.18% (1445) | 1.92 (1.81-2.04) | 1.97 (1.87-2.07) | 99.41% (1017) | 1.84 (1.75-1.94) | 1.9 (1.81-1.99) | 98.01% (1233) | 1.63 (1.49-1.76) | 1.6 (1.5-1.7) | 97.84% (679) |
| -1 | 2.87 (2.75-2.99) | 2.87 (2.79-2.96) | 98.79% (1473) | 2.33 (2.2-2.46) | 2.36 (2.25-2.46) | 99.71% (1023) | 2.81 (2.69-2.92) | 2.77 (2.67-2.88) | 99.44% (1252) | 2.06 (1.9-2.22) | 2.07 (1.96-2.19) | 98.13% (684) |
| Year total | 1.1 (1.02-1.17) | 1.36 (1.3-1.42) | - | 1.21 (1.11-1.3) | 1.45 (1.36-1.54) | - | 0.93 (0.86-1) | 1.24 (1.17-1.32) | - | 0.99 (0.89-1.09) | 1.2 (1.1-1.29) | - |
| *Mean number of consultations per patient per month. | | | | | | | | | | | | |
| **Age-adjusted mean number of consultations per patient per month, standardised to the Emergency Presenter group. | | | | | | | | | | | | |
| ***The cumulative proportion of patients who had at least one primary care consultation. | | | | | | | | | | | | |

**Supplementary Table S3.** Summary statistics for consultations with 6 relevant symptoms in the 12 months before diagnosis by diagnostic route.

| **Month before diagnosis** | **Emergency presentation (N=1491)** | | | **GP routine referral (N=1026)** | | | **Urgent referral (N=1259)** | | | **Other (N=697)** | | |
| --- | --- | --- | --- | --- | --- | --- | --- | --- | --- | --- | --- | --- |
| **Time** | **Mean consultations (95%CI)*** | **Age-standardised mean (95%CI)**** | **Cumulative proportion of patients***** | **Mean consultations (95%CI)*** | **Age-standardised mean (95%CI)**** | **Cumulative proportion of patients***** | **Mean consultations (95%CI)*** | **Age-standardised mean (95%CI)**** | **Cumulative proportion of patients***** | **Mean consultations (95%CI)*** | **Age-standardised mean (95%CI)**** | **Cumulative proportion of patients***** |
| -12 | 0.05 (0.04-0.06) | 0.05 (0.04-0.06) | 31.55% (454) | 0.05 (0.04-0.07) | 0.05 (0.04-0.06) | 33.94% (336) | 0.05 (0.04-0.07) | 0.06 (0.04-0.07) | 30.51% (375) | 0.04 (0.03-0.06) | 0.05 (0.03-0.07) | 32.74% (220) |
| -11 | 0.04 (0.03-0.05) | 0.04 (0.03-0.05) | 32.57% (470) | 0.06 (0.05-0.08) | 0.06 (0.05-0.08) | 35.72% (354) | 0.04 (0.03-0.05) | 0.04 (0.03-0.06) | 31.79% (392) | 0.06 (0.04-0.08) | 0.06 (0.04-0.08) | 34.03% (229) |
| -10 | 0.04 (0.03-0.05) | 0.04 (0.03-0.05) | 33.24% (482) | 0.06 (0.04-0.07) | 0.06 (0.04-0.08) | 37.78% (377) | 0.05 (0.03-0.06) | 0.05 (0.04-0.07) | 33.14% (409) | 0.06 (0.04-0.08) | 0.05 (0.04-0.07) | 36% (243) |
| -9 | 0.04 (0.03-0.05) | 0.04 (0.03-0.05) | 34.36% (500) | 0.06 (0.04-0.08) | 0.06 (0.04-0.07) | 38.97% (392) | 0.05 (0.04-0.06) | 0.06 (0.04-0.08) | 34.73% (430) | 0.06 (0.04-0.08) | 0.07 (0.05-0.1) | 37.91% (257) |
| -8 | 0.04 (0.03-0.05) | 0.04 (0.03-0.05) | 35.25% (515) | 0.06 (0.04-0.07) | 0.06 (0.04-0.07) | 40.85% (411) | 0.04 (0.03-0.05) | 0.04 (0.03-0.06) | 35.59% (441) | 0.04 (0.03-0.06) | 0.04 (0.03-0.06) | 39.62% (269) |
| -7 | 0.06 (0.04-0.07) | 0.06 (0.04-0.07) | 37.11% (544) | 0.08 (0.06-0.1) | 0.08 (0.06-0.1) | 42.91% (433) | 0.04 (0.03-0.06) | 0.05 (0.03-0.06) | 37.36% (464) | 0.06 (0.04-0.08) | 0.06 (0.04-0.08) | 40.85% (279) |
| -6 | 0.05 (0.04-0.06) | 0.05 (0.04-0.06) | 38.6% (567) | 0.1 (0.08-0.12) | 0.1 (0.08-0.12) | 45.41% (460) | 0.06 (0.05-0.08) | 0.07 (0.05-0.08) | 39.33% (490) | 0.06 (0.04-0.09) | 0.06 (0.04-0.08) | 41.9% (287) |
| -5 | 0.07 (0.05-0.08) | 0.07 (0.05-0.08) | 40.03% (590) | 0.08 (0.06-0.1) | 0.08 (0.06-0.1) | 47.83% (486) | 0.06 (0.04-0.07) | 0.06 (0.05-0.08) | 40.75% (509) | 0.07 (0.05-0.09) | 0.07 (0.05-0.09) | 43.42% (300) |
| -4 | 0.07 (0.06-0.09) | 0.07 (0.06-0.09) | 41.28% (611) | 0.11 (0.09-0.13) | 0.11 (0.09-0.14) | 50.44% (514) | 0.09 (0.07-0.1) | 0.09 (0.07-0.11) | 43.37% (543) | 0.08 (0.06-0.1) | 0.08 (0.06-0.11) | 44.65% (309) |
| -3 | 0.09 (0.07-0.1) | 0.09 (0.07-0.1) | 43.36% (643) | 0.15 (0.13-0.18) | 0.15 (0.12-0.17) | 54.51% (556) | 0.14 (0.12-0.16) | 0.15 (0.13-0.18) | 48.61% (610) | 0.12 (0.09-0.15) | 0.12 (0.09-0.15) | 48.85% (339) |
| -2 | 0.12 (0.1-0.14) | 0.12 (0.1-0.14) | 47.28% (703) | 0.2 (0.17-0.23) | 0.2 (0.17-0.23) | 60.31% (617) | 0.26 (0.23-0.29) | 0.27 (0.23-0.3) | 58.11% (731) | 0.14 (0.11-0.17) | 0.14 (0.11-0.17) | 51.87% (360) |
| -1 | 0.27 (0.24-0.3) | 0.27 (0.24-0.29) | 57.61% (859) | 0.19 (0.16-0.22) | 0.2 (0.17-0.24) | 65.2% (669) | 0.36 (0.32-0.39) | 0.35 (0.31-0.38) | 70.77% (891) | 0.19 (0.15-0.22) | 0.19 (0.16-0.23) | 57.39% (400) |
| Year total | 0.06 (0.05-0.07) | 0.06 (0.05-0.07) | - | 0.09 (0.07-0.11) | 0.09 (0.07-0.11) | - | 0.08 (0.06-0.1) | 0.09 (0.07-0.11) | - | 0.07 (0.05-0.09) | 0.07 (0.05-0.1) | - |
| *Mean number of symptomatic consultations per patient per month. | | | | | | | | | | | | |
| **Age-adjusted mean number of symptomatic consultations per patient per month, standardised to the Emergency Presenter group. | | | | | | | | | | | | |
| ***The cumulative proportion of patients who had at least one symptomatic consultation. | | | | | | | | | | | | |

**Supplementary Table S4.** Summary statistics for consultations with 3 main respiratory symptoms in the 12 months before diagnosis by diagnostic route.

| **Month before diagnosis** | **Emergency presentation (N=1491)** | | | **GP routine referral (N=1026)** | | | **Urgent referral (N=1259)** | | | **Other (N=697)** | | |
| --- | --- | --- | --- | --- | --- | --- | --- | --- | --- | --- | --- | --- |
| **Time** | **Mean consultations (95%CI)*** | **Age-standardised mean (95%CI)**** | **Cumulative proportion of patients***** | **Mean consultations (95%CI)*** | **Age-standardised mean (95%CI)**** | **Cumulative proportion of patients***** | **Mean consultations (95%CI)*** | **Age-standardised mean (95%CI)**** | **Cumulative proportion of patients***** | **Mean consultations (95%CI)*** | **Age-standardised mean (95%CI)**** | **Cumulative proportion of patients***** |
| -12 | 0.05 (0.04-0.06) | 0.05 (0.04-0.06) | 28.98% (417) | 0.05 (0.03-0.06) | 0.04 (0.03-0.06) | 31.92% (316) | 0.05 (0.04-0.07) | 0.05 (0.04-0.07) | 27.83% (342) | 0.04 (0.02-0.05) | 0.04 (0.03-0.07) | 29.32% (197) |
| -11 | 0.04 (0.03-0.05) | 0.04 (0.03-0.05) | 29.87% (431) | 0.06 (0.04-0.08) | 0.06 (0.04-0.07) | 33.6% (333) | 0.04 (0.03-0.05) | 0.04 (0.03-0.06) | 29.12% (359) | 0.05 (0.03-0.07) | 0.05 (0.03-0.07) | 30.16% (203) |
| -10 | 0.04 (0.03-0.05) | 0.04 (0.03-0.05) | 30.69% (445) | 0.05 (0.04-0.07) | 0.05 (0.04-0.07) | 35.47% (354) | 0.04 (0.03-0.05) | 0.05 (0.03-0.07) | 30.31% (374) | 0.05 (0.03-0.07) | 0.05 (0.03-0.07) | 31.85% (215) |
| -9 | 0.04 (0.03-0.05) | 0.04 (0.03-0.05) | 31.75% (462) | 0.06 (0.04-0.07) | 0.05 (0.04-0.06) | 36.48% (367) | 0.05 (0.03-0.06) | 0.05 (0.04-0.07) | 31.91% (395) | 0.06 (0.04-0.08) | 0.07 (0.05-0.1) | 33.48% (227) |
| -8 | 0.03 (0.02-0.04) | 0.03 (0.03-0.05) | 32.72% (478) | 0.05 (0.04-0.07) | 0.05 (0.04-0.07) | 38.27% (385) | 0.04 (0.03-0.05) | 0.04 (0.03-0.06) | 32.93% (408) | 0.04 (0.02-0.05) | 0.03 (0.02-0.05) | 34.9% (237) |
| -7 | 0.05 (0.04-0.06) | 0.05 (0.04-0.06) | 34.38% (504) | 0.07 (0.05-0.09) | 0.07 (0.05-0.09) | 40.14% (405) | 0.04 (0.03-0.05) | 0.04 (0.03-0.06) | 34.78% (432) | 0.06 (0.04-0.07) | 0.05 (0.04-0.08) | 35.87% (245) |
| -6 | 0.04 (0.03-0.05) | 0.04 (0.03-0.05) | 35.53% (522) | 0.09 (0.07-0.11) | 0.09 (0.07-0.12) | 42.25% (428) | 0.05 (0.04-0.07) | 0.06 (0.04-0.08) | 36.44% (454) | 0.05 (0.03-0.07) | 0.05 (0.03-0.07) | 37.08% (254) |
| -5 | 0.06 (0.05-0.07) | 0.06 (0.05-0.07) | 37.11% (547) | 0.08 (0.06-0.1) | 0.08 (0.06-0.1) | 44.39% (451) | 0.05 (0.04-0.06) | 0.06 (0.04-0.07) | 37.55% (469) | 0.06 (0.04-0.08) | 0.06 (0.04-0.08) | 38.35% (265) |
| -4 | 0.06 (0.05-0.08) | 0.06 (0.05-0.08) | 38.24% (566) | 0.1 (0.08-0.12) | 0.1 (0.08-0.12) | 46.91% (478) | 0.08 (0.06-0.09) | 0.08 (0.06-0.1) | 40.18% (503) | 0.07 (0.05-0.09) | 0.08 (0.05-0.1) | 39.45% (273) |
| -3 | 0.08 (0.06-0.09) | 0.08 (0.06-0.09) | 40.05% (594) | 0.14 (0.12-0.17) | 0.14 (0.11-0.16) | 50.69% (517) | 0.14 (0.11-0.16) | 0.14 (0.12-0.17) | 45.34% (569) | 0.11 (0.08-0.13) | 0.1 (0.08-0.13) | 42.8% (297) |
| -2 | 0.11 (0.09-0.13) | 0.11 (0.09-0.13) | 43.58% (648) | 0.18 (0.15-0.2) | 0.17 (0.14-0.19) | 55.72% (570) | 0.23 (0.21-0.26) | 0.23 (0.2-0.26) | 54.29% (683) | 0.13 (0.1-0.16) | 0.13 (0.1-0.16) | 46.25% (321) |
| -1 | 0.23 (0.2-0.26) | 0.23 (0.2-0.25) | 53.05% (791) | 0.16 (0.13-0.19) | 0.17 (0.14-0.2) | 59.45% (610) | 0.32 (0.29-0.36) | 0.31 (0.27-0.35) | 66.16% (833) | 0.16 (0.13-0.19) | 0.16 (0.13-0.19) | 50.93% (355) |
| Year total | 0.05 (0.04-0.07) | 0.05 (0.04-0.07) | - | 0.08 (0.07-0.1) | 0.08 (0.06-0.1) | - | 0.07 (0.06-0.09) | 0.08 (0.06-0.1) | - | 0.06 (0.04-0.08) | 0.06 (0.05-0.09) | - |
| *Mean number of symptomatic consultations per patient per month. | | | | | | | | | | | | |
| **Age-adjusted mean number of symptomatic consultations per patient per month, standardised to the Emergency Presenter group. | | | | | | | | | | | | |
| ***The cumulative proportion of patients who had at least one symptomatic consultation. | | | | | | | | | | | | |

**Supplementary Table S5.** Summary statistics for consultations with cough in the 12 months before diagnosis by diagnostic route.

| **Month before diagnosis** | **Emergency presentation (N=1491)** | | | **GP routine referral (N=1026)** | | | **Urgent referral (N=1259)** | | | **Other (N=697)** | | |
| --- | --- | --- | --- | --- | --- | --- | --- | --- | --- | --- | --- | --- |
| **Time** | **Mean consultations (95%CI)*** | **Age-standardised mean (95%CI)**** | **Cumulative proportion of patients***** | **Mean consultations (95%CI)*** | **Age-standardised mean (95%CI)**** | **Cumulative proportion of patients***** | **Mean consultations (95%CI)*** | **Age-standardised mean (95%CI)**** | **Cumulative proportion of patients***** | **Mean consultations (95%CI)*** | **Age-standardised mean (95%CI)**** | **Cumulative proportion of patients***** |
| -12 | 0.02 (0.01-0.03) | 0.02 (0.01-0.03) | 15.91% (229) | 0.02 (0.01-0.03) | 0.02 (0.01-0.03) | 18.99% (188) | 0.02 (0.02-0.03) | 0.02 (0.01-0.03) | 17.25% (212) | 0.02 (0.01-0.03) | 0.03 (0.01-0.05) | 16.52% (111) |
| -11 | 0.02 (0.01-0.02) | 0.02 (0.01-0.02) | 16.77% (242) | 0.03 (0.01-0.04) | 0.02 (0.01-0.03) | 19.88% (197) | 0.02 (0.01-0.03) | 0.02 (0.01-0.03) | 18.41% (227) | 0.03 (0.02-0.04) | 0.03 (0.02-0.05) | 17.24% (116) |
| -10 | 0.02 (0.01-0.02) | 0.02 (0.01-0.03) | 17.31% (251) | 0.03 (0.02-0.04) | 0.03 (0.02-0.04) | 21.14% (211) | 0.03 (0.02-0.04) | 0.03 (0.02-0.04) | 20.1% (248) | 0.04 (0.02-0.06) | 0.03 (0.02-0.05) | 18.96% (128) |
| -9 | 0.02 (0.01-0.03) | 0.02 (0.01-0.03) | 18.14% (264) | 0.03 (0.02-0.04) | 0.03 (0.02-0.04) | 22.27% (224) | 0.03 (0.02-0.03) | 0.03 (0.02-0.05) | 21.08% (261) | 0.04 (0.02-0.05) | 0.05 (0.03-0.08) | 20.5% (139) |
| -8 | 0.02 (0.01-0.03) | 0.02 (0.01-0.03) | 19.03% (278) | 0.03 (0.02-0.05) | 0.03 (0.02-0.05) | 24.06% (242) | 0.03 (0.02-0.03) | 0.03 (0.02-0.04) | 21.87% (271) | 0.02 (0.01-0.03) | 0.02 (0.01-0.03) | 21.65% (147) |
| -7 | 0.02 (0.01-0.02) | 0.02 (0.01-0.03) | 19.85% (291) | 0.04 (0.03-0.05) | 0.04 (0.03-0.05) | 25.77% (260) | 0.03 (0.02-0.04) | 0.03 (0.02-0.04) | 23.51% (292) | 0.01 (0-0.02) | 0.01 (0.01-0.03) | 22.11% (151) |
| -6 | 0.02 (0.01-0.03) | 0.02 (0.02-0.03) | 20.97% (308) | 0.04 (0.03-0.06) | 0.05 (0.03-0.06) | 27.44% (278) | 0.04 (0.03-0.05) | 0.04 (0.03-0.06) | 25.04% (312) | 0.03 (0.01-0.04) | 0.03 (0.02-0.04) | 22.92% (157) |
| -5 | 0.03 (0.02-0.04) | 0.03 (0.02-0.04) | 22.18% (327) | 0.05 (0.03-0.06) | 0.04 (0.03-0.06) | 29.82% (303) | 0.03 (0.02-0.04) | 0.04 (0.03-0.06) | 26.18% (327) | 0.04 (0.03-0.06) | 0.04 (0.03-0.06) | 24.17% (167) |
| -4 | 0.04 (0.02-0.05) | 0.04 (0.03-0.05) | 23.24% (344) | 0.06 (0.04-0.07) | 0.06 (0.04-0.08) | 31.89% (325) | 0.05 (0.04-0.06) | 0.05 (0.04-0.06) | 28.35% (355) | 0.04 (0.02-0.06) | 0.04 (0.03-0.06) | 25.14% (174) |
| -3 | 0.04 (0.03-0.05) | 0.04 (0.03-0.05) | 24.75% (367) | 0.08 (0.06-0.1) | 0.08 (0.06-0.1) | 35.2% (359) | 0.1 (0.08-0.11) | 0.1 (0.08-0.12) | 32.83% (412) | 0.06 (0.04-0.08) | 0.06 (0.04-0.08) | 27.38% (190) |
| -2 | 0.05 (0.04-0.06) | 0.05 (0.04-0.06) | 27.3% (406) | 0.11 (0.09-0.14) | 0.11 (0.09-0.14) | 39.69% (406) | 0.15 (0.12-0.17) | 0.14 (0.11-0.16) | 39.75% (500) | 0.07 (0.04-0.09) | 0.06 (0.04-0.08) | 29.83% (207) |
| -1 | 0.07 (0.06-0.09) | 0.07 (0.06-0.09) | 31.66% (472) | 0.09 (0.07-0.11) | 0.09 (0.07-0.11) | 42.2% (433) | 0.19 (0.16-0.21) | 0.17 (0.14-0.19) | 48.13% (606) | 0.07 (0.05-0.09) | 0.07 (0.05-0.09) | 31.99% (223) |
| Year total | 0.03 (0.02-0.03) | 0.03 (0.02-0.04) | - | 0.05 (0.03-0.06) | 0.05 (0.03-0.06) | - | 0.05 (0.04-0.06) | 0.05 (0.04-0.06) | - | 0.04 (0.02-0.05) | 0.04 (0.02-0.05) | - |
| *Mean number of symptomatic consultations per patient per month. | | | | | | | | | | | | |
| **Age-adjusted mean number of symptomatic consultations per patient per month, standardised to the Emergency Presenter group. | | | | | | | | | | | | |
| ***The cumulative proportion of patients who had at least one symptomatic consultation. | | | | | | | | | | | | |

**Supplementary Table S6.** Summary statistics for consultations with dyspnoea in the 12 months before diagnosis by diagnostic route.

| **Month before diagnosis** | **Emergency presentation (N=1491)** | | | **GP routine referral (N=1026)** | | | **Urgent referral (N=1259)** | | | **Other (N=697)** | | |
| --- | --- | --- | --- | --- | --- | --- | --- | --- | --- | --- | --- | --- |
| **Time** | **Mean consultations (95%CI)*** | **Age-standardised mean (95%CI)**** | **Cumulative proportion of patients***** | **Mean consultations (95%CI)*** | **Age-standardised mean (95%CI)**** | **Cumulative proportion of patients***** | **Mean consultations (95%CI)*** | **Age-standardised mean (95%CI)**** | **Cumulative proportion of patients***** | **Mean consultations (95%CI)*** | **Age-standardised mean (95%CI)**** | **Cumulative proportion of patients***** |
| -12 | 0.03 (0.02-0.04) | 0.03 (0.02-0.04) | 18.14% (261) | 0.02 (0.01-0.03) | 0.02 (0.01-0.03) | 18.48% (183) | 0.03 (0.02-0.04) | 0.03 (0.02-0.05) | 15.3% (188) | 0.02 (0.01-0.03) | 0.02 (0.01-0.03) | 18.3% (123) |
| -11 | 0.02 (0.01-0.03) | 0.02 (0.01-0.03) | 18.85% (272) | 0.03 (0.02-0.05) | 0.03 (0.02-0.05) | 19.58% (194) | 0.02 (0.01-0.02) | 0.02 (0.01-0.03) | 15.73% (194) | 0.02 (0.01-0.03) | 0.02 (0.01-0.03) | 18.87% (127) |
| -10 | 0.02 (0.01-0.03) | 0.02 (0.01-0.03) | 19.38% (281) | 0.02 (0.01-0.03) | 0.03 (0.02-0.04) | 20.44% (204) | 0.01 (0.01-0.02) | 0.02 (0.01-0.03) | 16.29% (201) | 0.01 (0-0.02) | 0.01 (0.01-0.03) | 19.41% (131) |
| -9 | 0.01 (0.01-0.02) | 0.01 (0.01-0.02) | 20% (291) | 0.02 (0.01-0.03) | 0.02 (0.01-0.03) | 20.87% (210) | 0.02 (0.01-0.03) | 0.02 (0.01-0.04) | 17.45% (216) | 0.02 (0.01-0.03) | 0.01 (0.01-0.03) | 20.21% (137) |
| -8 | 0.02 (0.01-0.02) | 0.02 (0.01-0.02) | 20.67% (302) | 0.02 (0.01-0.03) | 0.02 (0.01-0.03) | 21.57% (217) | 0.01 (0.01-0.02) | 0.01 (0.01-0.02) | 17.92% (222) | 0.02 (0.01-0.03) | 0.01 (0.01-0.03) | 20.91% (142) |
| -7 | 0.03 (0.02-0.04) | 0.03 (0.02-0.04) | 21.96% (322) | 0.03 (0.02-0.04) | 0.03 (0.02-0.04) | 22.5% (227) | 0.01 (0.01-0.02) | 0.02 (0.01-0.03) | 18.6% (231) | 0.04 (0.02-0.05) | 0.03 (0.02-0.05) | 21.67% (148) |
| -6 | 0.02 (0.01-0.03) | 0.02 (0.01-0.03) | 22.33% (328) | 0.05 (0.03-0.06) | 0.04 (0.03-0.06) | 23.59% (239) | 0.02 (0.01-0.02) | 0.02 (0.01-0.03) | 19.1% (238) | 0.02 (0.01-0.04) | 0.02 (0.01-0.04) | 22.19% (152) |
| -5 | 0.03 (0.02-0.04) | 0.03 (0.02-0.04) | 23.61% (348) | 0.03 (0.02-0.04) | 0.02 (0.02-0.04) | 24.11% (245) | 0.02 (0.01-0.02) | 0.02 (0.01-0.03) | 19.78% (247) | 0.02 (0.01-0.03) | 0.02 (0.01-0.03) | 22.58% (156) |
| -4 | 0.03 (0.02-0.04) | 0.03 (0.02-0.04) | 24.53% (363) | 0.03 (0.02-0.05) | 0.03 (0.02-0.04) | 25.02% (255) | 0.02 (0.01-0.03) | 0.03 (0.02-0.04) | 21.01% (263) | 0.03 (0.02-0.04) | 0.03 (0.02-0.05) | 23.55% (163) |
| -3 | 0.03 (0.02-0.04) | 0.03 (0.02-0.04) | 25.76% (382) | 0.05 (0.03-0.06) | 0.04 (0.03-0.05) | 27.16% (277) | 0.03 (0.02-0.04) | 0.04 (0.02-0.05) | 22.55% (283) | 0.04 (0.02-0.06) | 0.04 (0.02-0.05) | 24.93% (173) |
| -2 | 0.05 (0.04-0.06) | 0.05 (0.04-0.06) | 27.98% (416) | 0.04 (0.03-0.06) | 0.04 (0.03-0.05) | 28.84% (295) | 0.05 (0.04-0.07) | 0.06 (0.04-0.08) | 24.96% (314) | 0.06 (0.04-0.08) | 0.06 (0.04-0.09) | 26.95% (187) |
| -1 | 0.15 (0.12-0.17) | 0.15 (0.13-0.17) | 35.75% (533) | 0.06 (0.05-0.08) | 0.07 (0.05-0.09) | 31.87% (327) | 0.09 (0.07-0.11) | 0.1 (0.08-0.12) | 29.15% (367) | 0.07 (0.05-0.09) | 0.07 (0.05-0.1) | 30.13% (210) |
| Year total | 0.03 (0.02-0.04) | 0.03 (0.02-0.04) | - | 0.03 (0.02-0.04) | 0.03 (0.02-0.04) | - | 0.02 (0.01-0.03) | 0.03 (0.02-0.04) | - | 0.03 (0.01-0.04) | 0.03 (0.02-0.04) | - |
| *Mean number of symptomatic consultations per patient per month. | | | | | | | | | | | | |
| **Age-adjusted mean number of symptomatic consultations per patient per month, standardised to the Emergency Presenter group. | | | | | | | | | | | | |
| ***The cumulative proportion of patients who had at least one symptomatic consultation. | | | | | | | | | | | | |

**Supplementary Table S7.** Summary statistics for consultations with **haemoptysis** in the 12 months before diagnosis by diagnostic route.

| **Month before diagnosis** | **Emergency presentation (N=1491)** | | | **GP routine referral (N=1026)** | | | **Urgent referral (N=1259)** | | | **Other (N=697)** | | |
| --- | --- | --- | --- | --- | --- | --- | --- | --- | --- | --- | --- | --- |
| **Time** | **Mean consultations (95%CI)*** | **Age-standardised mean (95%CI)**** | **Cumulative proportion of patients***** | **Mean consultations (95%CI)*** | **Age-standardised mean (95%CI)**** | **Cumulative proportion of patients***** | **Mean consultations (95%CI)*** | **Age-standardised mean (95%CI)**** | **Cumulative proportion of patients***** | **Mean consultations (95%CI)*** | **Age-standardised mean (95%CI)**** | **Cumulative proportion of patients***** |
| -12 | 0 (0-0) | 0 (-) | 0.49% (7) | 0 (0-0) | 0 (0-0.01) | 1.52% (15) | 0 (0-0) | 0 (-) | 0.24% (3) | 0 (0-0) | 0 (-) | 0.74% (5) |
| -11 | 0 (0-0) | 0 (-) | 0.49% (7) | 0 (0-0) | 0 (0-0.01) | 1.72% (17) | 0 (0-0) | 0 (0-0) | 0.32% (4) | 0 (0-0) | 0 (0-0.01) | 0.74% (5) |
| -10 | 0 (0-0) | 0 (-) | 0.48% (7) | 0 (0-0) | 0 (-) | 1.7% (17) | 0 (0-0) | 0 (-) | 0.32% (4) | 0 (0-0) | 0 (-) | 0.74% (5) |
| -9 | 0 (0-0) | 0 (0-0) | 0.62% (9) | 0 (0-0.01) | 0 (0-0.01) | 1.69% (17) | 0 (0-0) | 0 (0-0) | 0.4% (5) | 0.01 (0-0.01) | 0.01 (0-0.02) | 1.03% (7) |
| -8 | 0 (0-0) | 0 (-) | 0.62% (9) | 0 (0-0) | 0 (0-0.01) | 1.89% (19) | 0 (0-0) | 0 (-) | 0.4% (5) | 0 (0-0) | 0 (0-0.02) | 1.18% (8) |
| -7 | 0 (0-0) | 0 (0-0.01) | 0.82% (12) | 0 (0-0) | 0 (0-0.01) | 2.08% (21) | 0 (0-0) | 0 (-) | 0.4% (5) | 0 (0-0.01) | 0 (0-0.01) | 1.46% (10) |
| -6 | 0 (0-0) | 0 (-) | 0.82% (12) | 0 (0-0.01) | 0.01 (0-0.01) | 2.57% (26) | 0 (0-0) | 0 (-) | 0.4% (5) | 0 (0-0) | 0 (0-0.01) | 1.61% (11) |
| -5 | 0 (0-0) | 0 (0-0) | 0.81% (12) | 0.01 (0-0.01) | 0.01 (0-0.02) | 2.95% (30) | 0 (0-0) | 0 (0-0) | 0.48% (6) | 0 (0-0.01) | 0 (0-0.01) | 1.88% (13) |
| -4 | 0 (0-0) | 0 (-) | 0.81% (12) | 0.01 (0-0.01) | 0.01 (0-0.02) | 3.43% (35) | 0 (0-0.01) | 0 (0-0.01) | 0.8% (10) | 0 (0-0) | 0 (0-0.01) | 1.88% (13) |
| -3 | 0 (0-0.01) | 0 (0-0.01) | 1.15% (17) | 0.01 (0.01-0.02) | 0.01 (0.01-0.03) | 4.51% (46) | 0 (0-0.01) | 0 (0-0.01) | 1.2% (15) | 0.01 (0-0.01) | 0.01 (0-0.02) | 2.31% (16) |
| -2 | 0.0 (0-0.01) | 0 (0-0.01) | 1.55% (23) | 0.02 (0.01-0.02) | 0.02 (0.01-0.03) | 5.87% (60) | 0.04 (0.02-0.05) | 0.04 (0.03-0.05) | 3.97% (50) | 0 (0-0.01) | 0 (0-0.01) | 2.59% (18) |
| -1 | 0.01 (0-0.01) | 0.01 (0-0.01) | 2.35% (35) | 0.01 (0.01-0.02) | 0.01 (0.01-0.02) | 6.92% (71) | 0.05 (0.04-0.06) | 0.05 (0.03-0.07) | 8.26% (104) | 0.02 (0-0.03) | 0.02 (0.01-0.03) | 3.59% (25) |
| **Year total** | 0 (0-0) | 0 (-) | - | 0.01 (0-0.01) | 0.01 (-) | - | 0 (0-0.01) | 0 (-) | - | 0 (0-0) | 0 (-) | - |
| *Mean number of symptomatic consultations per patient per month. | | | | | | | | | | | | |
| **Age-adjusted mean number of symptomatic consultations per patient per month, standardised to the Emergency Presenter group. | | | | | | | | | | | | |
| ***The cumulative proportion of patients who had at least one symptomatic consultation. | | | | | | | | | | | | |

**Supplementary Table S8.** Summary statistics for any chest imaging events in the 12 months before diagnosis by diagnostic route.

Pre-diagnostic rates of any chest imaging events

| **Month before diagnosis** | **Emergency presentation (N=1491)** | | | **GP routine referral (N=1026)** | | | **Urgent referral (N=1259)** | | | **Other (N=697)** | | |
| --- | --- | --- | --- | --- | --- | --- | --- | --- | --- | --- | --- | --- |
| **Time** | **Mean consultations (95%CI)*** | **Age-standardised mean (95%CI)**** | **Cumulative proportion of patients***** | **Mean consultations (95%CI)*** | **Age-standardised mean (95%CI)**** | **Cumulative proportion of patients***** | **Mean consultations (95%CI)*** | **Age-standardised mean (95%CI)**** | **Cumulative proportion of patients***** | **Mean consultations (95%CI)*** | **Age-standardised mean (95%CI)**** | **Cumulative proportion of patients***** |
| -12 | 0.02 (0.01-0.03) | 0.02 (-0.25-0.37) | 8.69% (125) | 0.02 (0.01-0.03) | 0.02 (-0.24-0.39) | 12.02% (119) | 0.01 (0-0.02) | 0.01 (-0.18-0.29) | 6.51% (80) | 0.01 (0-0.02) | 0.01 (-0.14-0.28) | 9.67% (65) |
| -11 | 0.01 (0.01-0.02) | 0.01 (-0.2-0.31) | 9.22% (133) | 0.02 (0.01-0.04) | 0.02 (-0.25-0.4) | 12.41% (123) | 0 (0-0.01) | 0 (-0.09-0.21) | 6.65% (82) | 0.02 (0.01-0.03) | 0.02 (-0.23-0.4) | 10.25% (69) |
| -10 | 0.02 (0.01-0.03) | 0.02 (-0.23-0.35) | 10% (145) | 0.01 (0.01-0.02) | 0.01 (-0.19-0.32) | 12.63% (126) | 0.01 (0-0.01) | 0.01 (-0.12-0.22) | 6.97% (86) | 0.02 (0.01-0.03) | 0.02 (-0.21-0.37) | 11.11% (75) |
| -9 | 0.02 (0.01-0.03) | 0.02 (-0.24-0.36) | 10.79% (157) | 0.02 (0.01-0.03) | 0.02 (-0.23-0.36) | 13.02% (131) | 0.01 (0-0.02) | 0.01 (-0.17-0.29) | 7.43% (92) | 0.03 (0.01-0.04) | 0.02 (-0.23-0.39) | 11.8% (80) |
| -8 | 0.02 (0.01-0.02) | 0.02 (-0.21-0.32) | 11.09% (162) | 0.03 (0.01-0.04) | 0.03 (-0.28-0.44) | 13.92% (140) | 0.01 (0-0.01) | 0.01 (-0.15-0.28) | 7.75% (96) | 0.02 (0.01-0.03) | 0.02 (-0.22-0.39) | 12.08% (82) |
| -7 | 0.02 (0.01-0.03) | 0.02 (-0.23-0.35) | 11.66% (171) | 0.02 (0.01-0.03) | 0.02 (-0.21-0.34) | 14.77% (149) | 0.02 (0.01-0.03) | 0.02 (-0.23-0.38) | 8.86% (110) | 0.03 (0.01-0.04) | 0.03 (-0.28-0.48) | 13.03% (89) |
| -6 | 0.03 (0.02-0.04) | 0.03 (-0.28-0.41) | 12.66% (186) | 0.04 (0.03-0.06) | 0.05 (-0.37-0.58) | 16.58% (168) | 0.01 (0.01-0.02) | 0.02 (-0.2-0.33) | 9.63% (120) | 0.03 (0.02-0.05) | 0.03 (-0.29-0.48) | 13.87% (95) |
| -5 | 0.04 (0.03-0.06) | 0.04 (-0.34-0.5) | 13.98% (206) | 0.04 (0.03-0.06) | 0.04 (-0.34-0.53) | 18.11% (184) | 0.02 (0.01-0.03) | 0.02 (-0.2-0.32) | 9.85% (123) | 0.04 (0.02-0.06) | 0.04 (-0.33-0.54) | 14.91% (103) |
| -4 | 0.03 (0.02-0.05) | 0.03 (-0.3-0.44) | 15.07% (223) | 0.07 (0.05-0.09) | 0.09 (-0.46-0.74) | 20.71% (211) | 0.03 (0.02-0.04) | 0.03 (-0.27-0.42) | 11.1% (139) | 0.06 (0.04-0.08) | 0.07 (-0.41-0.66) | 17.05% (118) |
| -3 | 0.06 (0.05-0.08) | 0.06 (-0.4-0.6) | 17.13% (254) | 0.11 (0.09-0.14) | 0.11 (-0.52-0.84) | 25.1% (256) | 0.08 (0.06-0.1) | 0.09 (-0.47-0.74) | 15.54% (195) | 0.08 (0.05-0.1) | 0.07 (-0.42-0.68) | 19.6% (136) |
| -2 | 0.11 (0.09-0.14) | 0.11 (-0.52-0.82) | 20.71% (308) | 0.22 (0.19-0.26) | 0.22 (-0.67-1.19) | 32.75% (335) | 0.25 (0.22-0.29) | 0.27 (-0.72-1.33) | 27.34% (344) | 0.19 (0.15-0.23) | 0.2 (-0.64-1.15) | 25.07% (174) |
| -1 | 0.63 (0.58-0.69) | 0.63 (-0.9-2.24) | 40.51% (604) | 0.43 (0.38-0.48) | 0.42 (-0.82-1.74) | 43.76% (449) | 0.53 (0.48-0.57) | 0.53 (-0.87-2.01) | 43.84% (552) | 0.35 (0.29-0.4) | 0.31 (-0.75-1.48) | 35.01% (244) |
| Year total | 0.04 (0.02-0.05) | 0.04 (-0.29-0.44) | - | 0.06 (0.04-0.07) | 0.06 (-0.34-0.56) | - | 0.04 (0.03-0.05) | 0.04 (-0.25-0.44) | - | 0.05 (0.03-0.06) | 0.05 (-0.31-0.53) | - |
| *Mean number of imaging events per patient per month. | | | | | | | | | | | | |
| **Age-adjusted mean number of imaging events per patient per month, standardised to the Emergency Presenter group. | | | | | | | | | | | | |
| ***The cumulative proportion of patients who had at least one imaging event. | | | | | | | | | | | | |

**Supplementary Table S9.** Summary statistics for chest X-rays in the 12 months before diagnosis by diagnostic route.

| **Month before diagnosis** | **Emergency presentation (N=1491)** | | | **GP routine referral (N=1026)** | | | **Urgent referral (N=1259)** | | | **Other (N=697)** | | |
| --- | --- | --- | --- | --- | --- | --- | --- | --- | --- | --- | --- | --- |
| **Time** | **Mean consultations (95%CI)*** | **Age-standardised mean (95%CI)**** | **Cumulative proportion of patients***** | **Mean consultations (95%CI)*** | **Age-standardised mean (95%CI)**** | **Cumulative proportion of patients***** | **Mean consultations (95%CI)*** | **Age-standardised mean (95%CI)**** | **Cumulative proportion of patients***** | **Mean consultations (95%CI)*** | **Age-standardised mean (95%CI)**** | **Cumulative proportion of patients***** |
| -12 | 0.02 (0.01-0.03) | 0.02 (-0.22-0.34) | 8.41% (121) | 0.02 (0.01-0.03) | 0.02 (-0.22-0.36) | 11.41% (113) | 0.01 (0-0.02) | 0.02 (-0.22-0.34) | 6.35% (78) | 0.01 (0-0.02) | 0.01 (-0.14-0.28) | 8.48% (57) |
| -11 | 0.01 (0.01-0.02) | 0.01 (-0.19-0.3) | 8.94% (129) | 0.02 (0.01-0.03) | 0.02 (-0.21-0.35) | 11.81% (117) | 0 (0-0.01) | 0.01 (-0.19-0.3) | 6.49% (80) | 0.02 (0.01-0.03) | 0.02 (-0.22-0.39) | 9.21% (62) |
| -10 | 0.02 (0.01-0.03) | 0.02 (-0.21-0.32) | 9.59% (139) | 0.01 (0-0.02) | 0.01 (-0.17-0.3) | 12.12% (121) | 0.01 (0-0.01) | 0.02 (-0.21-0.32) | 6.81% (84) | 0.01 (0-0.02) | 0.01 (-0.18-0.34) | 9.93% (67) |
| -9 | 0.02 (0.01-0.03) | 0.02 (-0.22-0.34) | 10.31% (150) | 0.01 (0.01-0.02) | 0.01 (-0.17-0.29) | 12.43% (125) | 0.01 (0-0.02) | 0.02 (-0.22-0.34) | 7.27% (90) | 0.02 (0-0.04) | 0.02 (-0.21-0.35) | 10.62% (72) |
| -8 | 0.01 (0.01-0.02) | 0.01 (-0.2-0.31) | 10.54% (154) | 0.02 (0.01-0.04) | 0.02 (-0.25-0.4) | 13.32% (134) | 0 (0-0.01) | 0.01 (-0.2-0.31) | 7.59% (94) | 0.01 (0-0.02) | 0.01 (-0.18-0.33) | 10.9% (74) |
| -7 | 0.02 (0.01-0.03) | 0.02 (-0.22-0.34) | 11.19% (164) | 0.01 (0.01-0.02) | 0.01 (-0.18-0.3) | 14.07% (142) | 0.02 (0.01-0.03) | 0.02 (-0.22-0.34) | 8.7% (108) | 0.02 (0.01-0.03) | 0.02 (-0.24-0.41) | 11.71% (80) |
| -6 | 0.03 (0.02-0.04) | 0.03 (-0.27-0.4) | 12.12% (178) | 0.03 (0.02-0.05) | 0.04 (-0.33-0.54) | 15.6% (158) | 0.01 (0.01-0.02) | 0.03 (-0.27-0.4) | 9.47% (118) | 0.02 (0.01-0.03) | 0.02 (-0.24-0.42) | 12.26% (84) |
| -5 | 0.04 (0.02-0.05) | 0.04 (-0.31-0.46) | 13.43% (198) | 0.03 (0.02-0.04) | 0.03 (-0.3-0.47) | 17.13% (174) | 0.01 (0-0.02) | 0.04 (-0.31-0.46) | 9.69% (121) | 0.03 (0.02-0.04) | 0.03 (-0.28-0.47) | 13.46% (93) |
| -4 | 0.03 (0.02-0.04) | 0.03 (-0.28-0.42) | 14.46% (214) | 0.05 (0.03-0.06) | 0.06 (-0.38-0.61) | 19.14% (195) | 0.03 (0.02-0.03) | 0.03 (-0.28-0.42) | 10.94% (137) | 0.04 (0.03-0.06) | 0.05 (-0.36-0.6) | 15.46% (107) |
| -3 | 0.05 (0.03-0.06) | 0.05 (-0.35-0.52) | 16.05% (238) | 0.08 (0.06-0.09) | 0.08 (-0.43-0.68) | 23.04% (235) | 0.06 (0.05-0.08) | 0.05 (-0.35-0.52) | 15.22% (191) | 0.05 (0.03-0.07) | 0.05 (-0.34-0.55) | 17.87% (124) |
| -2 | 0.09 (0.07-0.11) | 0.09 (-0.47-0.72) | 19.5% (290) | 0.14 (0.11-0.17) | 0.14 (-0.56-0.93) | 28.93% (296) | 0.17 (0.15-0.2) | 0.09 (-0.47-0.72) | 26.23% (330) | 0.12 (0.09-0.15) | 0.13 (-0.53-0.9) | 22.91% (159) |
| -1 | 0.47 (0.43-0.52) | 0.47 (-0.85-1.87) | 38.7% (577) | 0.24 (0.21-0.28) | 0.24 (-0.69-1.27) | 38.89% (399) | 0.25 (0.22-0.28) | 0.47 (-0.85-1.87) | 40.75% (513) | 0.21 (0.18-0.25) | 0.19 (-0.63-1.12) | 32.14% (224) |
| Year total | 0.03 (0.02-0.04) | 0.03 (-0.27-0.41) | - | 0.04 (0.03-0.05) | 0.04 (-0.29-0.47) | - | 0.03 (0.02-0.04) | 0.03 (-0.27-0.41) | - | 0.03 (0.02-0.05) | 0.04 (-0.27-0.46) | - |
| *Mean number of imaging events per patient per month. | | | | | | | | | | | | |
| **Age-adjusted mean number of imaging events per patient per month, standardised to the Emergency Presenter group. | | | | | | | | | | | | |
| ***The cumulative proportion of patients who had at least one imaging event. | | | | | | | | | | | | |

**Supplementary Table S10.** Summary statistics for chest CT scans in the 12 months before diagnosis by diagnostic route.

| **Month before diagnosis** | **Emergency presentation (N=1491)** | | | **GP routine referral (N=1026)** | | | **Urgent referral (N=1259)** | | | **Other (N=697)** | | |
| --- | --- | --- | --- | --- | --- | --- | --- | --- | --- | --- | --- | --- |
| **Time** | **Mean consultations (95%CI)*** | **Age-standardised mean (95%CI)**** | **Cumulative proportion of patients***** | **Mean consultations (95%CI)*** | **Age-standardised mean (95%CI)**** | **Cumulative proportion of patients***** | **Mean consultations (95%CI)*** | **Age-standardised mean (95%CI)**** | **Cumulative proportion of patients***** | **Mean consultations (95%CI)*** | **Age-standardised mean (95%CI)**** | **Cumulative proportion of patients***** |
| -12 | 0 (0-0.01) | 0 (-0.1-0.19) | 1.53% (22) | 0.01 (0-0.01) | 0 (-0.1-0.2) | 3.74% (37) | 0 (0-0) | 0 (-0.1-0.19) | 0.73% (9) | 0 (0-0) | 0 (-) | 2.68% (18) |
| -11 | 0 (0-0) | 0 (-0.03-0.12) | 1.52% (22) | 0.01 (0-0.01) | 0.01 (-0.12-0.26) | 3.83% (38) | 0 (0-0) | 0 (-0.03-0.12) | 0.73% (9) | 0 (0-0.01) | 0 (-0.06-0.17) | 2.67% (18) |
| -10 | 0 (0-0.01) | 0 (-0.09-0.18) | 1.79% (26) | 0 (0-0.01) | 0 (-0.07-0.17) | 3.91% (39) | 0 (0-0) | 0 (-0.09-0.18) | 0.73% (9) | 0 (0-0.01) | 0 (-0.07-0.21) | 2.81% (19) |
| -9 | 0 (0-0.01) | 0 (-0.09-0.18) | 1.99% (29) | 0.01 (0-0.01) | 0.01 (-0.14-0.25) | 4.17% (42) | 0 (0-0) | 0 (-0.09-0.18) | 0.73% (9) | 0 (0-0.01) | 0 (-0.09-0.22) | 2.95% (20) |
| -8 | 0 (0-0.01) | 0 (-0.07-0.17) | 2.12% (31) | 0 (0-0.01) | 0.01 (-0.11-0.23) | 4.37% (44) | 0 (0-0) | 0 (-0.07-0.17) | 0.81% (10) | 0 (0-0.01) | 0.01 (-0.11-0.28) | 3.24% (22) |
| -7 | 0 (0-0) | 0 (-0.06-0.15) | 2.25% (33) | 0 (0-0.01) | 0 (-0.1-0.22) | 4.56% (46) | 0 (0-0) | 0 (-0.06-0.15) | 0.81% (10) | 0.01 (0-0.01) | 0.01 (-0.14-0.29) | 3.95% (27) |
| -6 | 0 (0-0.01) | 0 (-0.07-0.17) | 2.45% (36) | 0.01 (0.01-0.02) | 0.01 (-0.16-0.27) | 5.43% (55) | 0 (0-0) | 0 (-0.07-0.17) | 0.88% (11) | 0.01 (0-0.02) | 0.01 (-0.14-0.29) | 4.82% (33) |
| -5 | 0.01 (0-0.01) | 0.01 (-0.12-0.22) | 2.85% (42) | 0.01 (0-0.02) | 0.01 (-0.15-0.28) | 6% (61) | 0 (0-0) | 0.01 (-0.12-0.22) | 0.96% (12) | 0.01 (0-0.02) | 0.01 (-0.16-0.31) | 5.5% (38) |
| -4 | 0 (0-0.01) | 0 (-0.08-0.18) | 3.04% (45) | 0.03 (0.02-0.04) | 0.03 (-0.28-0.44) | 7.65% (78) | 0 (0-0.01) | 0 (-0.08-0.18) | 1.2% (15) | 0.02 (0.01-0.03) | 0.01 (-0.18-0.33) | 6.65% (46) |
| -3 | 0.01 (0.01-0.02) | 0.01 (-0.2-0.31) | 4.25% (63) | 0.04 (0.03-0.05) | 0.04 (-0.33-0.5) | 10.49% (107) | 0.01 (0.01-0.02) | 0.01 (-0.2-0.31) | 2.47% (31) | 0.03 (0.02-0.04) | 0.03 (-0.25-0.41) | 8.36% (58) |
| -2 | 0.03 (0.02-0.03) | 0.03 (-0.27-0.4) | 6.39% (95) | 0.08 (0.07-0.1) | 0.08 (-0.45-0.7) | 16.91% (173) | 0.09 (0.07-0.1) | 0.03 (-0.27-0.4) | 10.49% (132) | 0.07 (0.05-0.09) | 0.07 (-0.42-0.68) | 13.98% (97) |
| -1 | 0.18 (0.16-0.2) | 0.18 (-0.63-1.06) | 22.87% (341) | 0.2 (0.18-0.23) | 0.19 (-0.64-1.11) | 34.41% (353) | 0.28 (0.26-0.31) | 0.18 (-0.63-1.06) | 36.93% (465) | 0.14 (0.11-0.17) | 0.13 (-0.54-0.91) | 25.68% (179) |
| Year total | 0.01 (0-0.01) | 0.01 (-0.11-0.2) | - | 0.02 (0.01-0.03) | 0.02 (-0.18-0.32) | - | 0.01 (0.01-0.01) | 0.01 (-0.11-0.2) | - | 0.01 (0.01-0.02) | 0.01 (-) | - |
| *Mean number of imaging events per patient per month. | | | | | | | | | | | | |
| **Age-adjusted mean number of imaging events per patient per month, standardised to the Emergency Presenter group. | | | | | | | | | | | | |
| ***The cumulative proportion of patients who had at least one imaging event. | | | | | | | | | | | | |

**Supplementary Table S11.** Modelled incidence rate ratios (IRRs) for pre-diagnostic healthcare use*, by diagnostic route, with and without adjustment for socio-demographic factors ( -12 to -2 months).

# Primary care consultations (-12 to -2)

| **Variable** |  | **IRR** | **95% CI** | **p-value** | **IRR** | **95% CI** | **p-value** | **IRR** | **95% CI** | **p-value** | **IRR** | **95% CI** | **p-value** |
| --- | --- | --- | --- | --- | --- | --- | --- | --- | --- | --- | --- | --- | --- |
| Diagnostic route | GP referral | 1.16 | 1.09-1.24 | 0.000 | 1.34 | 1.26-1.42 | 0.000 | 1.34 | 1.25-1.42 | 0.000 | 1.33 | 1.25-1.42 | 0.000 |
|  | TWW | 0.89 | 0.84-0.95 | 0.000 | 1.11 | 1.04-1.18 | 0.001 | 1.11 | 1.04-1.18 | 0.001 | 1.11 | 1.04-1.18 | 0.001 |
|  | Other | 0.93 | 0.86-1 | 0.049 | 1.07 | 1-1.15 | 0.060 | 1.07 | 1-1.15 | 0.065 | 1.06 | 0.98-1.14 | 0.123 |
| Age at diagnosis | 85-99 |  |  |  | 1.08 | 1-1.16 | 0.048 | 1.08 | 1-1.16 | 0.044 | 1.08 | 1-1.16 | 0.053 |
|  | 30-64 |  |  |  | 0.79 | 0.74-0.84 | 0.000 | 0.79 | 0.74-0.84 | 0.000 | 0.79 | 0.74-0.84 | 0.000 |
|  | 65-74 |  |  |  | 0.91 | 0.86-0.96 | 0.001 | 0.91 | 0.86-0.96 | 0.001 | 0.91 | 0.86-0.96 | 0.001 |
| Gender | Female |  |  |  | 1.13 | 1.08-1.18 | 0.000 | 1.13 | 1.08-1.18 | 0.000 | 1.13 | 1.08-1.18 | 0.000 |
| Ethnicity | Non-White |  |  |  | 0.86 | 0.78-0.96 | 0.009 | 0.87 | 0.78-0.97 | 0.009 | 0.87 | 0.78-0.97 | 0.010 |
|  | Unknown |  |  |  | 0.79 | 0.66-0.93 | 0.006 | 0.79 | 0.66-0.94 | 0.007 | 0.78 | 0.66-0.93 | 0.005 |
| IMD | 2 |  |  |  | 0.95 | 0.88-1.02 | 0.149 | 0.95 | 0.88-1.02 | 0.151 | 0.95 | 0.88-1.02 | 0.146 |
|  | 3 |  |  |  | 1.02 | 0.95-1.09 | 0.634 | 1.02 | 0.95-1.09 | 0.638 | 1.02 | 0.95-1.09 | 0.659 |
|  | 4 |  |  |  | 0.91 | 0.85-0.98 | 0.014 | 0.91 | 0.85-0.98 | 0.014 | 0.91 | 0.85-0.98 | 0.016 |
|  | 5 - Most deprived |  |  |  | 0.89 | 0.83-0.96 | 0.002 | 0.89 | 0.83-0.96 | 0.003 | 0.89 | 0.82-0.96 | 0.002 |
| Smoker status | Non-smoker |  |  |  | 1.00 | 0.92-1.08 | 0.901 | 1.00 | 0.92-1.08 | 0.940 | 1.00 | 0.92-1.08 | 0.939 |
| COPD status | Pre-existing COPD (at -24 months or earlier) |  |  |  | 1.10 | 1.02-1.19 | 0.016 | 1.10 | 1.02-1.19 | 0.016 | 1.10 | 1.02-1.19 | 0.013 |
|  | No COPD |  |  |  | 0.85 | 0.79-0.91 | 0.000 | 0.85 | 0.79-0.91 | 0.000 | 0.85 | 0.79-0.91 | 0.000 |
| Elixhauser comorbidity score | 1 |  |  |  | 1.20 | 1.12-1.29 | 0.000 | 1.20 | 1.12-1.29 | 0.000 | 1.20 | 1.11-1.29 | 0.000 |
|  | 2 |  |  |  | 1.30 | 1.21-1.4 | 0.000 | 1.30 | 1.21-1.4 | 0.000 | 1.30 | 1.21-1.4 | 0.000 |
|  | 3+ |  |  |  | 1.67 | 1.56-1.78 | 0.000 | 1.67 | 1.56-1.78 | 0.000 | 1.67 | 1.57-1.78 | 0.000 |
| Morphology | NSCLC |  |  |  |  |  |  | 0.97 | 0.9-1.05 | 0.449 | 0.96 | 0.89-1.04 | 0.320 |
|  | Unspecified |  |  |  |  |  |  | 0.97 | 0.89-1.05 | 0.428 | 0.95 | 0.87-1.03 | 0.215 |
| Stage at diagnosis | Unknown |  |  |  |  |  |  |  |  |  | 1.10 | 1.05-1.16 | 0.000 |
|  | Not advanced |  |  |  |  |  |  |  |  |  | 1.08 | 1.01-1.16 | 0.021 |

# 6 selected relevant symptoms (-12 to -2)

| **Variable** |  | **IRR** | **95% CI** | **p-value** | **IRR** | **95% CI** | **p-value** | **IRR** | **95% CI** | **p-value** | **IRR** | **95% CI** | **p-value** |
| --- | --- | --- | --- | --- | --- | --- | --- | --- | --- | --- | --- | --- | --- |
| Diagnostic route | GP referral | 1.60 | 1.42-1.81 | 0.000 | 1.71 | 1.51-1.94 | 0.000 | 1.68 | 1.48-1.9 | 0.000 | 1.66 | 1.47-1.89 | 0.000 |
|  | TWW | 1.41 | 1.25-1.59 | 0.000 | 1.59 | 1.4-1.79 | 0.000 | 1.53 | 1.35-1.73 | 0.000 | 1.52 | 1.34-1.73 | 0.000 |
|  | Other | 1.22 | 1.05-1.41 | 0.007 | 1.31 | 1.14-1.51 | 0.000 | 1.29 | 1.11-1.49 | 0.001 | 1.27 | 1.1-1.47 | 0.001 |
| Age at diagnosis | 85-99 |  |  |  | 0.92 | 0.79-1.06 | 0.250 | 0.95 | 0.82-1.11 | 0.541 | 0.95 | 0.82-1.11 | 0.528 |
|  | 30-64 |  |  |  | 0.87 | 0.77-0.99 | 0.038 | 0.85 | 0.75-0.97 | 0.012 | 0.85 | 0.75-0.97 | 0.014 |
|  | 65-74 |  |  |  | 0.90 | 0.81-1.01 | 0.069 | 0.89 | 0.8-0.99 | 0.036 | 0.89 | 0.8-0.99 | 0.037 |
| Gender | Female |  |  |  | 1.07 | 0.98-1.17 | 0.129 | 1.07 | 0.98-1.17 | 0.125 | 1.07 | 0.98-1.17 | 0.127 |
| Ethnicity | Non-White |  |  |  | 1.00 | 0.8-1.24 | 0.970 | 1.01 | 0.81-1.26 | 0.922 | 1.01 | 0.81-1.26 | 0.905 |
|  | Unknown |  |  |  | 0.79 | 0.55-1.13 | 0.192 | 0.84 | 0.58-1.2 | 0.334 | 0.83 | 0.58-1.2 | 0.327 |
| IMD | 2 |  |  |  | 0.87 | 0.75-1.01 | 0.068 | 0.88 | 0.76-1.02 | 0.084 | 0.88 | 0.76-1.02 | 0.084 |
|  | 3 |  |  |  | 0.92 | 0.8-1.07 | 0.277 | 0.92 | 0.8-1.07 | 0.283 | 0.92 | 0.8-1.07 | 0.283 |
|  | 4 |  |  |  | 0.82 | 0.71-0.95 | 0.008 | 0.83 | 0.72-0.96 | 0.010 | 0.83 | 0.72-0.96 | 0.011 |
|  | 5 - Most deprived |  |  |  | 0.86 | 0.75-1 | 0.047 | 0.87 | 0.76-1.01 | 0.064 | 0.87 | 0.75-1.01 | 0.061 |
| Smoker status | Non-smoker |  |  |  | 0.95 | 0.81-1.13 | 0.569 | 0.96 | 0.81-1.13 | 0.627 | 0.96 | 0.81-1.14 | 0.642 |
| COPD status | Pre-existing COPD (at -24 months or earlier) |  |  |  | 1.35 | 1.18-1.55 | 0.000 | 1.37 | 1.2-1.58 | 0.000 | 1.37 | 1.2-1.58 | 0.000 |
|  | No COPD |  |  |  | 0.54 | 0.48-0.62 | 0.000 | 0.54 | 0.48-0.62 | 0.000 | 0.54 | 0.48-0.62 | 0.000 |
| Elixhauser comorbidity score | 1 |  |  |  | 1.16 | 1-1.34 | 0.045 | 1.16 | 1.01-1.35 | 0.039 | 1.16 | 1-1.34 | 0.045 |
|  | 2 |  |  |  | 1.04 | 0.89-1.21 | 0.616 | 1.04 | 0.9-1.21 | 0.597 | 1.04 | 0.89-1.21 | 0.625 |
|  | 3+ |  |  |  | 1.14 | 1-1.3 | 0.045 | 1.14 | 1-1.3 | 0.044 | 1.14 | 1-1.3 | 0.047 |
| Morphology | NSCLC |  |  |  |  |  |  | 0.88 | 0.76-1.02 | 0.084 | 0.87 | 0.75-1.01 | 0.064 |
|  | Unspecified |  |  |  |  |  |  | 0.77 | 0.65-0.91 | 0.002 | 0.76 | 0.64-0.9 | 0.001 |
| Stage at diagnosis | Unknown |  |  |  |  |  |  |  |  |  | 1.04 | 0.94-1.15 | 0.394 |
|  | Not advanced |  |  |  |  |  |  |  |  |  | 1.07 | 0.94-1.21 | 0.309 |

# 3 selected main respiratory symptoms (-12 to -2)

| **Variable** |  | **IRR** | **95% CI** | **p-value** | **IRR** | **95% CI** | **p-value** | **IRR** | **95% CI** | **p-value** | **IRR** | **95% CI** | **p-value** |
| --- | --- | --- | --- | --- | --- | --- | --- | --- | --- | --- | --- | --- | --- |
| Diagnostic route | GP referral | 1.62 | 1.42-1.84 | 0.000 | 1.72 | 1.51-1.96 | 0.000 | 1.68 | 1.47-1.92 | 0.000 | 1.67 | 1.46-1.91 | 0.000 |
|  | TWW | 1.44 | 1.27-1.63 | 0.000 | 1.62 | 1.42-1.84 | 0.000 | 1.55 | 1.36-1.78 | 0.000 | 1.55 | 1.35-1.77 | 0.000 |
|  | Other | 1.19 | 1.02-1.39 | 0.025 | 1.28 | 1.1-1.5 | 0.001 | 1.26 | 1.08-1.47 | 0.004 | 1.24 | 1.07-1.45 | 0.006 |
| Age at diagnosis | 85-99 |  |  |  | 0.90 | 0.77-1.05 | 0.186 | 0.94 | 0.8-1.1 | 0.421 | 0.93 | 0.8-1.1 | 0.403 |
|  | 30-64 |  |  |  | 0.90 | 0.78-1.03 | 0.116 | 0.87 | 0.76-1 | 0.047 | 0.87 | 0.76-1 | 0.051 |
|  | 65-74 |  |  |  | 0.95 | 0.84-1.06 | 0.364 | 0.93 | 0.83-1.05 | 0.239 | 0.93 | 0.83-1.05 | 0.243 |
| Gender | Female |  |  |  | 1.07 | 0.98-1.18 | 0.138 | 1.08 | 0.98-1.18 | 0.133 | 1.08 | 0.98-1.18 | 0.133 |
| Ethnicity | Non-White |  |  |  | 1.04 | 0.83-1.31 | 0.740 | 1.06 | 0.84-1.33 | 0.645 | 1.06 | 0.84-1.33 | 0.630 |
|  | Unknown |  |  |  | 0.73 | 0.49-1.09 | 0.124 | 0.78 | 0.52-1.16 | 0.224 | 0.78 | 0.52-1.16 | 0.216 |
| IMD | 2 |  |  |  | 0.87 | 0.75-1.02 | 0.089 | 0.88 | 0.75-1.03 | 0.108 | 0.88 | 0.75-1.03 | 0.107 |
|  | 3 |  |  |  | 0.91 | 0.78-1.06 | 0.206 | 0.91 | 0.78-1.06 | 0.211 | 0.91 | 0.78-1.06 | 0.209 |
|  | 4 |  |  |  | 0.83 | 0.72-0.97 | 0.019 | 0.84 | 0.72-0.98 | 0.025 | 0.84 | 0.72-0.98 | 0.025 |
|  | 5 - Most deprived |  |  |  | 0.83 | 0.71-0.97 | 0.017 | 0.84 | 0.72-0.98 | 0.024 | 0.84 | 0.72-0.97 | 0.022 |
| Smoker status | Non-smoker |  |  |  | 0.94 | 0.79-1.13 | 0.507 | 0.95 | 0.79-1.14 | 0.565 | 0.95 | 0.79-1.14 | 0.582 |
| COPD status | Pre-existing COPD (at -24 months or earlier) |  |  |  | 1.37 | 1.19-1.59 | 0.000 | 1.39 | 1.21-1.61 | 0.000 | 1.39 | 1.21-1.61 | 0.000 |
|  | No COPD |  |  |  | 0.50 | 0.43-0.57 | 0.000 | 0.50 | 0.43-0.57 | 0.000 | 0.50 | 0.43-0.57 | 0.000 |
| Elixhauser comorbidity score | 1 |  |  |  | 1.14 | 0.97-1.33 | 0.103 | 1.14 | 0.98-1.33 | 0.091 | 1.14 | 0.97-1.33 | 0.103 |
|  | 2 |  |  |  | 1.00 | 0.85-1.17 | 0.987 | 1.00 | 0.85-1.17 | 0.984 | 1.00 | 0.85-1.17 | 0.979 |
|  | 3+ |  |  |  | 1.11 | 0.97-1.27 | 0.138 | 1.11 | 0.97-1.27 | 0.138 | 1.11 | 0.97-1.27 | 0.143 |
| Morphology | NSCLC |  |  |  |  |  |  | 0.87 | 0.74-1.01 | 0.072 | 0.86 | 0.73-1 | 0.052 |
|  | Unspecified |  |  |  |  |  |  | 0.75 | 0.63-0.9 | 0.002 | 0.74 | 0.62-0.89 | 0.001 |
| Stage at diagnosis | Unknown |  |  |  |  |  |  |  |  |  | 1.07 | 0.96-1.19 | 0.242 |
|  | Not advanced |  |  |  |  |  |  |  |  |  | 1.09 | 0.95-1.25 | 0.223 |

# Cough consultations (-12 to -2)

| **Variable** |  | **IRR** | **95% CI** | **p-value** | **IRR** | **95% CI** | **p-value** | **IRR** | **95% CI** | **p-value** | **IRR** | **95% CI** | **p-value** |
| --- | --- | --- | --- | --- | --- | --- | --- | --- | --- | --- | --- | --- | --- |
| Diagnostic route | GP referral | 1.90 | 1.58-2.28 | 0.000 | 1.90 | 1.58-2.3 | 0.000 | 1.82 | 1.51-2.2 | 0.000 | 1.81 | 1.49-2.19 | 0.000 |
|  | TWW | 1.90 | 1.6-2.27 | 0.000 | 1.94 | 1.61-2.33 | 0.000 | 1.81 | 1.5-2.18 | 0.000 | 1.80 | 1.49-2.18 | 0.000 |
|  | Other | 1.28 | 1.03-1.59 | 0.025 | 1.30 | 1.04-1.62 | 0.019 | 1.25 | 1-1.56 | 0.050 | 1.23 | 0.99-1.54 | 0.066 |
| Age at diagnosis | 85-99 |  |  |  | 0.90 | 0.71-1.12 | 0.347 | 0.96 | 0.76-1.21 | 0.736 | 0.96 | 0.76-1.21 | 0.721 |
|  | 30-64 |  |  |  | 1.04 | 0.87-1.26 | 0.657 | 0.99 | 0.82-1.19 | 0.911 | 0.99 | 0.82-1.2 | 0.931 |
|  | 65-74 |  |  |  | 0.99 | 0.84-1.17 | 0.927 | 0.96 | 0.81-1.14 | 0.658 | 0.96 | 0.82-1.14 | 0.664 |
| Gender | Female |  |  |  | 1.16 | 1.01-1.32 | 0.034 | 1.16 | 1.01-1.32 | 0.030 | 1.16 | 1.01-1.32 | 0.031 |
| Ethnicity | Non-White |  |  |  | 1.08 | 0.79-1.48 | 0.621 | 1.11 | 0.81-1.52 | 0.504 | 1.12 | 0.82-1.53 | 0.488 |
|  | Unknown |  |  |  | 0.54 | 0.3-0.99 | 0.045 | 0.60 | 0.33-1.1 | 0.100 | 0.60 | 0.33-1.1 | 0.098 |
| IMD | 2 |  |  |  | 0.87 | 0.7-1.09 | 0.221 | 0.88 | 0.71-1.1 | 0.263 | 0.88 | 0.71-1.1 | 0.265 |
|  | 3 |  |  |  | 0.92 | 0.75-1.14 | 0.460 | 0.93 | 0.75-1.15 | 0.482 | 0.93 | 0.75-1.15 | 0.484 |
|  | 4 |  |  |  | 0.86 | 0.69-1.06 | 0.156 | 0.87 | 0.7-1.07 | 0.190 | 0.87 | 0.7-1.07 | 0.196 |
|  | 5 - Most deprived |  |  |  | 0.79 | 0.63-0.98 | 0.034 | 0.80 | 0.64-1 | 0.048 | 0.80 | 0.64-1 | 0.047 |
| Smoker status | Non-smoker |  |  |  | 1.03 | 0.81-1.3 | 0.813 | 1.04 | 0.82-1.31 | 0.764 | 1.04 | 0.82-1.32 | 0.746 |
| COPD status | Pre-existing COPD (at -24 months or earlier) |  |  |  | 0.94 | 0.76-1.16 | 0.554 | 0.96 | 0.77-1.19 | 0.706 | 0.96 | 0.77-1.19 | 0.707 |
|  | No COPD |  |  |  | 0.64 | 0.52-0.77 | 0.000 | 0.63 | 0.52-0.77 | 0.000 | 0.64 | 0.52-0.77 | 0.000 |
| Elixhauser comorbidity score | 1 |  |  |  | 1.11 | 0.9-1.36 | 0.341 | 1.11 | 0.9-1.37 | 0.316 | 1.11 | 0.9-1.37 | 0.336 |
|  | 2 |  |  |  | 1.02 | 0.82-1.26 | 0.892 | 1.02 | 0.82-1.26 | 0.886 | 1.01 | 0.82-1.26 | 0.908 |
|  | 3+ |  |  |  | 1.00 | 0.83-1.21 | 0.982 | 1.00 | 0.83-1.21 | 0.998 | 1.00 | 0.83-1.21 | 0.995 |
| Morphology | NSCLC |  |  |  |  |  |  | 0.84 | 0.68-1.05 | 0.122 | 0.83 | 0.67-1.03 | 0.099 |
|  | Unspecified |  |  |  |  |  |  | 0.65 | 0.51-0.84 | 0.001 | 0.64 | 0.5-0.83 | 0.001 |
| Stage at diagnosis | Unknown |  |  |  |  |  |  |  |  |  | 1.06 | 0.91-1.23 | 0.449 |
|  | Not advanced |  |  |  |  |  |  |  |  |  | 1.09 | 0.9-1.32 | 0.378 |

# Dyspnoea consultations (-12 to -2)

| **Variable** |  | **IRR** | **95% CI** | **p-value** | **IRR** | **95% CI** | **p-value** | **IRR** | **95% CI** | **p-value** | **IRR** | **95% CI** | **p-value** |
| --- | --- | --- | --- | --- | --- | --- | --- | --- | --- | --- | --- | --- | --- |
| Diagnostic route | GP referral | 1.18 | 0.97-1.44 | 0.100 | 1.33 | 1.1-1.61 | 0.004 | 1.35 | 1.11-1.64 | 0.003 | 1.34 | 1.1-1.63 | 0.004 |
|  | TWW | 0.88 | 0.72-1.07 | 0.204 | 1.07 | 0.88-1.3 | 0.528 | 1.07 | 0.88-1.31 | 0.488 | 1.07 | 0.88-1.31 | 0.499 |
|  | Other | 1.01 | 0.8-1.27 | 0.934 | 1.13 | 0.9-1.41 | 0.287 | 1.14 | 0.91-1.42 | 0.259 | 1.13 | 0.9-1.41 | 0.291 |
| Age at diagnosis | 85-99 |  |  |  | 0.91 | 0.72-1.14 | 0.402 | 0.90 | 0.71-1.15 | 0.404 | 0.90 | 0.71-1.14 | 0.395 |
|  | 30-64 |  |  |  | 0.73 | 0.59-0.91 | 0.005 | 0.74 | 0.59-0.92 | 0.007 | 0.74 | 0.59-0.92 | 0.007 |
|  | 65-74 |  |  |  | 0.95 | 0.8-1.12 | 0.516 | 0.95 | 0.8-1.13 | 0.562 | 0.95 | 0.8-1.13 | 0.566 |
| Gender | Female |  |  |  | 1.03 | 0.89-1.18 | 0.729 | 1.02 | 0.88-1.18 | 0.780 | 1.02 | 0.89-1.18 | 0.767 |
| Ethnicity | Non-White |  |  |  | 0.87 | 0.59-1.29 | 0.494 | 0.87 | 0.59-1.28 | 0.485 | 0.87 | 0.59-1.28 | 0.479 |
|  | Unknown |  |  |  | 1.20 | 0.7-2.07 | 0.511 | 1.19 | 0.69-2.06 | 0.523 | 1.19 | 0.69-2.05 | 0.536 |
| IMD | 2 |  |  |  | 0.89 | 0.7-1.13 | 0.327 | 0.89 | 0.7-1.12 | 0.322 | 0.89 | 0.7-1.12 | 0.317 |
|  | 3 |  |  |  | 0.88 | 0.7-1.12 | 0.302 | 0.88 | 0.7-1.11 | 0.287 | 0.88 | 0.7-1.11 | 0.284 |
|  | 4 |  |  |  | 0.80 | 0.63-1.02 | 0.068 | 0.80 | 0.63-1.01 | 0.064 | 0.80 | 0.63-1.01 | 0.063 |
|  | 5 - Most deprived |  |  |  | 0.87 | 0.69-1.1 | 0.236 | 0.87 | 0.69-1.09 | 0.220 | 0.86 | 0.69-1.09 | 0.213 |
| Smoker status | Non-smoker |  |  |  | 0.74 | 0.53-1.04 | 0.082 | 0.75 | 0.54-1.05 | 0.097 | 0.75 | 0.54-1.05 | 0.097 |
| COPD status | Pre-existing COPD (at -24 months or earlier) |  |  |  | 1.89 | 1.56-2.29 | 0.000 | 1.89 | 1.56-2.29 | 0.000 | 1.89 | 1.56-2.3 | 0.000 |
|  | No COPD |  |  |  | 0.29 | 0.24-0.36 | 0.000 | 0.29 | 0.24-0.36 | 0.000 | 0.29 | 0.24-0.36 | 0.000 |
| Elixhauser comorbidity score | 1 |  |  |  | 1.13 | 0.88-1.47 | 0.334 | 1.14 | 0.88-1.47 | 0.314 | 1.14 | 0.88-1.47 | 0.333 |
|  | 2 |  |  |  | 1.04 | 0.8-1.35 | 0.765 | 1.04 | 0.81-1.35 | 0.739 | 1.04 | 0.8-1.35 | 0.761 |
|  | 3+ |  |  |  | 1.26 | 1.01-1.57 | 0.037 | 1.27 | 1.02-1.58 | 0.034 | 1.27 | 1.02-1.57 | 0.035 |
| Morphology | NSCLC |  |  |  |  |  |  | 0.85 | 0.67-1.08 | 0.184 | 0.84 | 0.66-1.07 | 0.162 |
|  | Unspecified |  |  |  |  |  |  | 0.89 | 0.68-1.16 | 0.380 | 0.88 | 0.67-1.14 | 0.326 |
| Stage at diagnosis | Unknown |  |  |  |  |  |  |  |  |  | 1.06 | 0.9-1.24 | 0.492 |
|  | Not advanced |  |  |  |  |  |  |  |  |  | 1.06 | 0.87-1.3 | 0.569 |

# Haemoptysis consultations (-12 to -2)

| **Variable** |  | **IRR** | **95% CI** | **p-value** | **IRR** | **95% CI** | **p-value** | **IRR** | **95% CI** | **p-value** | **IRR** | **95% CI** | **p-value** |
| --- | --- | --- | --- | --- | --- | --- | --- | --- | --- | --- | --- | --- | --- |
| Diagnostic route | GP referral | 3.75 | 1.09-12.95 | 0.037 | 3.25 | 1.33-7.96 | 0.010 | 2.42 | 0.93-6.29 | 0.070 | 4.05 | 1.12-14.66 | 0.033 |
|  | TWW | 3.06 | 0.89-10.58 | 0.077 | 2.92 | 1.19-7.18 | 0.020 | 2.03 | 0.78-5.31 | 0.149 | 3.51 | 0.94-13.12 | 0.062 |
|  | Other | 1.83 | 0.39-8.59 | 0.446 | 1.62 | 0.44-5.95 | 0.467 | 1.27 | 0.34-4.69 | 0.718 | 2.04 | 0.43-9.66 | 0.371 |
| Age at diagnosis | 85-99 |  |  |  | 0.93 | 0.27-3.18 | 0.908 | 0.87 | 0.25-3.04 | 0.823 | 0.99 | 0.26-3.85 | 0.992 |
|  | 30-64 |  |  |  | 0.65 | 0.65-0.65 | 0.000 | 0.69 | 0.69-0.69 | 0.000 | 0.68 | 0.21-2.19 | 0.520 |
|  | 65-74 |  |  |  | 0.68 | 0.28-1.65 | 0.396 | 0.73 | 0.31-1.7 | 0.460 | 0.68 | 0.25-1.82 | 0.444 |
| Gender | Female |  |  |  | 0.67 | 0.66-0.67 | 0.000 | 0.61 | 0.61-0.61 | 0.000 | 0.71 | 0.31-1.64 | 0.428 |
| Ethnicity | Non-White |  |  |  | 1.38 | 0.24-8.02 | 0.722 | 0.95 | 0.13-6.78 | 0.958 | 1.02 | 0.13-7.95 | 0.983 |
|  | Unknown |  |  |  | 0.05 | 0-908.51 | 0.546 | 0.23 | 0-90.45 | 0.630 | 0.52 | 0.01-42.62 | 0.773 |
| IMD | 2 |  |  |  | 0.71 | 0.71-0.72 | 0.000 | 0.68 | 0.68-0.68 | 0.000 | 0.94 | 0.24-3.72 | 0.932 |
|  | 3 |  |  |  | 1.00 | 0.37-2.68 | 0.997 | 0.87 | 0.33-2.27 | 0.769 | 1.30 | 0.36-4.68 | 0.685 |
|  | 4 |  |  |  | 0.63 | 0.2-1.93 | 0.415 | 0.59 | 0.2-1.73 | 0.335 | 0.82 | 0.2-3.27 | 0.776 |
|  | 5 - Most deprived |  |  |  | 0.89 | 0.32-2.47 | 0.825 | 0.73 | 0.27-2 | 0.540 | 1.12 | 0.3-4.2 | 0.872 |
| Smoker status | Non-smoker |  |  |  | 0.60 | 0.6-0.6 | 0.000 | 0.64 | 0.64-0.64 | 0.000 | 0.69 | 0.12-4.05 | 0.680 |
| COPD status | Pre-existing COPD (at -24 months or earlier) |  |  |  | 0.86 | 0.36-2.06 | 0.731 | 0.76 | 0.32-1.76 | 0.516 | 1.06 | 0.31-3.61 | 0.930 |
|  | No COPD |  |  |  | 0.53 | 0.53-0.54 | 0.000 | 0.41 | 0.4-0.41 | 0.000 | 0.67 | 0.21-2.11 | 0.497 |
| Elixhauser comorbidity score | 1 |  |  |  | 1.04 | 0.36-3 | 0.944 | 0.85 | 0.29-2.47 | 0.765 | 1.35 | 0.37-4.99 | 0.649 |
|  | 2 |  |  |  | 0.87 | 0.28-2.75 | 0.813 | 0.80 | 0.27-2.42 | 0.697 | 1.10 | 0.27-4.49 | 0.898 |
|  | 3+ |  |  |  | 1.07 | 1.07-1.07 | 0.000 | 0.88 | 0.88-0.88 | 0.000 | 1.51 | 0.46-4.93 | 0.497 |
| Morphology | NSCLC |  |  |  |  |  |  | 0.67 | 0.3-1.46 | 0.312 | 1.19 | 0.28-5.07 | 0.811 |
|  | Unspecified |  |  |  |  |  |  | 0.56 | 0.56-0.56 | 0.000 | 0.89 | 0.17-4.75 | 0.889 |
| Stage at diagnosis | Unknown |  |  |  |  |  |  |  |  |  | 1.38 | 0.56-3.43 | 0.482 |
|  | Not advanced |  |  |  |  |  |  |  |  |  | 1.25 | 0.41-3.79 | 0.699 |

# Any chest imaging event (-12 to -2)

| **Variable** |  | **IRR** | **95% CI** | **p-value** | **IRR** | **95% CI** | **p-value** | **IRR** | **95% CI** | **p-value** | **IRR** | **95% CI** | **p-value** |
| --- | --- | --- | --- | --- | --- | --- | --- | --- | --- | --- | --- | --- | --- |
| Diagnostic route | GP referral | 2.00 | 1.61-2.49 | 0.000 | 2.45 | 1.97-3.05 | 0.000 | 2.28 | 1.82-2.84 | 0.000 | 1.84 | 1.48-2.29 | 0.000 |
|  | TWW | 1.43 | 1.16-1.77 | 0.001 | 1.95 | 1.56-2.44 | 0.000 | 1.77 | 1.41-2.22 | 0.000 | 1.36 | 1.09-1.7 | 0.006 |
|  | Other | 1.48 | 1.15-1.9 | 0.002 | 1.75 | 1.37-2.25 | 0.000 | 1.63 | 1.27-2.1 | 0.000 | 1.37 | 1.07-1.75 | 0.012 |
| Age at diagnosis | 85-99 |  |  |  | 1.11 | 0.86-1.43 | 0.411 | 1.22 | 0.94-1.57 | 0.135 | 1.26 | 0.98-1.61 | 0.069 |
|  | 30-64 |  |  |  | 0.94 | 0.75-1.18 | 0.591 | 0.88 | 0.7-1.1 | 0.273 | 0.87 | 0.7-1.08 | 0.215 |
|  | 65-74 |  |  |  | 0.90 | 0.74-1.1 | 0.312 | 0.86 | 0.71-1.05 | 0.147 | 0.88 | 0.73-1.07 | 0.191 |
| Gender | Female |  |  |  | 1.10 | 0.94-1.29 | 0.238 | 1.11 | 0.95-1.3 | 0.187 | 1.06 | 0.91-1.23 | 0.460 |
| Ethnicity | Non-White |  |  |  | 0.68 | 0.45-1.03 | 0.072 | 0.71 | 0.47-1.08 | 0.107 | 0.74 | 0.5-1.12 | 0.156 |
|  | Unknown |  |  |  | 0.96 | 0.53-1.74 | 0.897 | 1.11 | 0.61-2.02 | 0.724 | 1.29 | 0.72-2.3 | 0.397 |
| IMD | 2 |  |  |  | 0.93 | 0.72-1.2 | 0.572 | 0.95 | 0.73-1.23 | 0.687 | 0.93 | 0.73-1.2 | 0.592 |
|  | 3 |  |  |  | 0.93 | 0.72-1.2 | 0.570 | 0.94 | 0.73-1.21 | 0.630 | 0.94 | 0.74-1.21 | 0.644 |
|  | 4 |  |  |  | 0.92 | 0.71-1.19 | 0.522 | 0.94 | 0.73-1.21 | 0.641 | 0.94 | 0.73-1.2 | 0.593 |
|  | 5 - Most deprived |  |  |  | 0.87 | 0.67-1.13 | 0.293 | 0.90 | 0.7-1.17 | 0.436 | 0.92 | 0.72-1.19 | 0.535 |
| Smoker status | Non-smoker |  |  |  | 1.32 | 1.01-1.73 | 0.042 | 1.31 | 1-1.71 | 0.052 | 1.41 | 1.09-1.83 | 0.009 |
| COPD status | Pre-existing COPD (at -24 months or earlier) |  |  |  | 1.05 | 0.82-1.36 | 0.682 | 1.09 | 0.84-1.4 | 0.520 | 0.97 | 0.76-1.24 | 0.838 |
|  | No COPD |  |  |  | 0.63 | 0.5-0.8 | 0.000 | 0.63 | 0.5-0.79 | 0.000 | 0.63 | 0.51-0.79 | 0.000 |
| Elixhauser comorbidity score | 1 |  |  |  | 1.31 | 1.01-1.71 | 0.041 | 1.30 | 1-1.69 | 0.050 | 1.22 | 0.94-1.58 | 0.126 |
|  | 2 |  |  |  | 1.20 | 0.91-1.57 | 0.189 | 1.19 | 0.91-1.56 | 0.212 | 1.09 | 0.83-1.41 | 0.541 |
|  | 3+ |  |  |  | 2.05 | 1.63-2.58 | 0.000 | 2.03 | 1.61-2.56 | 0.000 | 1.86 | 1.49-2.33 | 0.000 |
| Morphology | NSCLC |  |  |  |  |  |  | 1.19 | 0.91-1.56 | 0.208 | 0.97 | 0.74-1.26 | 0.808 |
|  | Unspecified |  |  |  |  |  |  | 0.79 | 0.58-1.08 | 0.139 | 0.85 | 0.63-1.15 | 0.303 |
| Stage at diagnosis | Unknown |  |  |  |  |  |  |  |  |  | 0.25 | 0.2-0.31 | 0.000 |
|  | Not advanced |  |  |  |  |  |  |  |  |  | 2.29 | 1.89-2.77 | 0.000 |

# Chest X-rays (-12 to -2)

| **Variable** |  | **IRR** | **95% CI** | **p-value** | **IRR** | **95% CI** | **p-value** | **IRR** | **95% CI** | **p-value** | **IRR** | **95% CI** | **p-value** |
| --- | --- | --- | --- | --- | --- | --- | --- | --- | --- | --- | --- | --- | --- |
| Diagnostic route | GP referral | 1.55 | 1.25-1.93 | 0.000 | 1.91 | 1.53-2.38 | 0.000 | 1.82 | 1.46-2.27 | 0.000 | 1.56 | 1.25-1.95 | 0.000 |
|  | TWW | 1.31 | 1.06-1.61 | 0.012 | 1.77 | 1.42-2.21 | 0.000 | 1.65 | 1.32-2.07 | 0.000 | 1.34 | 1.07-1.67 | 0.010 |
|  | Other | 1.24 | 0.96-1.59 | 0.098 | 1.46 | 1.14-1.88 | 0.003 | 1.40 | 1.08-1.8 | 0.010 | 1.24 | 0.96-1.59 | 0.095 |
| Age at diagnosis | 85-99 |  |  |  | 1.18 | 0.92-1.52 | 0.184 | 1.27 | 0.98-1.63 | 0.068 | 1.31 | 1.02-1.69 | 0.032 |
|  | 30-64 |  |  |  | 0.96 | 0.77-1.2 | 0.710 | 0.91 | 0.73-1.15 | 0.431 | 0.90 | 0.72-1.13 | 0.357 |
|  | 65-74 |  |  |  | 0.87 | 0.71-1.06 | 0.166 | 0.84 | 0.69-1.03 | 0.093 | 0.85 | 0.7-1.04 | 0.109 |
| Gender | Female |  |  |  | 1.07 | 0.91-1.25 | 0.417 | 1.08 | 0.92-1.26 | 0.363 | 1.04 | 0.89-1.21 | 0.641 |
| Ethnicity | Non-White |  |  |  | 0.68 | 0.45-1.04 | 0.074 | 0.70 | 0.46-1.07 | 0.098 | 0.73 | 0.48-1.11 | 0.143 |
|  | Unknown |  |  |  | 1.08 | 0.6-1.93 | 0.801 | 1.20 | 0.67-2.15 | 0.547 | 1.40 | 0.79-2.5 | 0.250 |
| IMD | 2 |  |  |  | 0.93 | 0.72-1.21 | 0.590 | 0.94 | 0.73-1.22 | 0.663 | 0.93 | 0.72-1.2 | 0.584 |
|  | 3 |  |  |  | 0.91 | 0.7-1.17 | 0.445 | 0.91 | 0.7-1.18 | 0.470 | 0.91 | 0.71-1.17 | 0.467 |
|  | 4 |  |  |  | 0.88 | 0.68-1.14 | 0.322 | 0.89 | 0.69-1.15 | 0.375 | 0.89 | 0.69-1.14 | 0.342 |
|  | 5 - Most deprived |  |  |  | 0.91 | 0.7-1.17 | 0.463 | 0.93 | 0.72-1.2 | 0.570 | 0.95 | 0.74-1.22 | 0.681 |
| Smoker status | Non-smoker |  |  |  | 1.31 | 1-1.71 | 0.051 | 1.31 | 1-1.71 | 0.054 | 1.39 | 1.07-1.82 | 0.014 |
| COPD status | Pre-existing COPD (at -24 months or earlier) |  |  |  | 1.06 | 0.83-1.37 | 0.626 | 1.09 | 0.85-1.4 | 0.511 | 0.98 | 0.77-1.25 | 0.872 |
|  | No COPD |  |  |  | 0.64 | 0.51-0.8 | 0.000 | 0.63 | 0.5-0.79 | 0.000 | 0.62 | 0.5-0.78 | 0.000 |
| Elixhauser comorbidity score | 1 |  |  |  | 1.14 | 0.88-1.49 | 0.326 | 1.14 | 0.87-1.48 | 0.344 | 1.09 | 0.84-1.42 | 0.517 |
|  | 2 |  |  |  | 1.04 | 0.79-1.37 | 0.781 | 1.03 | 0.79-1.36 | 0.810 | 0.97 | 0.74-1.27 | 0.807 |
|  | 3+ |  |  |  | 1.85 | 1.47-2.34 | 0.000 | 1.84 | 1.46-2.32 | 0.000 | 1.73 | 1.38-2.17 | 0.000 |
| Morphology | NSCLC |  |  |  |  |  |  | 1.02 | 0.78-1.34 | 0.870 | 0.89 | 0.68-1.17 | 0.400 |
|  | Unspecified |  |  |  |  |  |  | 0.77 | 0.57-1.05 | 0.101 | 0.86 | 0.63-1.17 | 0.333 |
| Stage at diagnosis | Unknown |  |  |  |  |  |  |  |  |  | 0.27 | 0.21-0.33 | 0.000 |
|  | Not advanced |  |  |  |  |  |  |  |  |  | 1.73 | 1.42-2.11 | 0.000 |

# Chest CT scans (-12 to -2)

| **Variable** |  | **IRR** | **95% CI** | **p-value** | **IRR** | **95% CI** | **p-value** | **IRR** | **95% CI** | **p-value** | **IRR** | **95% CI** | **p-value** |
| --- | --- | --- | --- | --- | --- | --- | --- | --- | --- | --- | --- | --- | --- |
| Diagnostic route | GP referral | 2.90 | 2.12-3.96 | 0.000 | 3.39 | 2.47-4.66 | 0.000 | 2.97 | 2.16-4.08 | 0.000 | 2.23 | 1.66-3.01 | 0.000 |
|  | TWW | 1.61 | 1.16-2.22 | 0.004 | 2.07 | 1.48-2.9 | 0.000 | 1.77 | 1.26-2.49 | 0.001 | 1.29 | 0.94-1.78 | 0.119 |
|  | Other | 2.31 | 1.62-3.29 | 0.000 | 2.63 | 1.84-3.75 | 0.000 | 2.34 | 1.64-3.33 | 0.000 | 1.73 | 1.24-2.42 | 0.001 |
| Age at diagnosis | 85-99 |  |  |  | 0.88 | 0.6-1.29 | 0.516 | 1.00 | 0.68-1.47 | 0.990 | 1.01 | 0.71-1.45 | 0.940 |
|  | 30-64 |  |  |  | 0.90 | 0.65-1.24 | 0.503 | 0.82 | 0.59-1.13 | 0.221 | 0.81 | 0.6-1.09 | 0.159 |
|  | 65-74 |  |  |  | 1.01 | 0.77-1.33 | 0.925 | 0.95 | 0.72-1.24 | 0.693 | 0.98 | 0.76-1.26 | 0.850 |
| Gender | Female |  |  |  | 1.13 | 0.9-1.42 | 0.280 | 1.15 | 0.92-1.44 | 0.211 | 1.08 | 0.88-1.33 | 0.472 |
| Ethnicity | Non-White |  |  |  | 0.72 | 0.38-1.33 | 0.290 | 0.76 | 0.41-1.41 | 0.379 | 0.80 | 0.44-1.45 | 0.459 |
|  | Unknown |  |  |  | 0.73 | 0.28-1.9 | 0.520 | 0.96 | 0.37-2.46 | 0.930 | 0.98 | 0.4-2.4 | 0.958 |
| IMD | 2 |  |  |  | 1.00 | 0.69-1.45 | 0.985 | 1.04 | 0.72-1.5 | 0.833 | 1.01 | 0.72-1.42 | 0.944 |
|  | 3 |  |  |  | 1.02 | 0.71-1.47 | 0.901 | 1.05 | 0.73-1.5 | 0.792 | 1.05 | 0.75-1.47 | 0.772 |
|  | 4 |  |  |  | 1.08 | 0.76-1.55 | 0.667 | 1.13 | 0.79-1.61 | 0.503 | 1.13 | 0.81-1.57 | 0.474 |
|  | 5 - Most deprived |  |  |  | 0.83 | 0.57-1.21 | 0.324 | 0.88 | 0.61-1.28 | 0.506 | 0.88 | 0.62-1.25 | 0.471 |
| Smoker status | Non-smoker |  |  |  | 1.26 | 0.86-1.86 | 0.237 | 1.22 | 0.83-1.78 | 0.315 | 1.31 | 0.91-1.87 | 0.142 |
| COPD status | Pre-existing COPD (at -24 months or earlier) |  |  |  | 0.88 | 0.62-1.26 | 0.489 | 0.93 | 0.65-1.31 | 0.666 | 0.87 | 0.63-1.2 | 0.384 |
|  | No COPD |  |  |  | 0.67 | 0.49-0.93 | 0.016 | 0.66 | 0.48-0.91 | 0.011 | 0.71 | 0.53-0.96 | 0.025 |
| Elixhauser comorbidity score | 1 |  |  |  | 1.68 | 1.14-2.48 | 0.008 | 1.64 | 1.11-2.4 | 0.012 | 1.48 | 1.03-2.12 | 0.034 |
|  | 2 |  |  |  | 1.66 | 1.12-2.46 | 0.012 | 1.63 | 1.1-2.41 | 0.015 | 1.43 | 0.99-2.08 | 0.057 |
|  | 3+ |  |  |  | 2.31 | 1.64-3.27 | 0.000 | 2.27 | 1.61-3.2 | 0.000 | 2.02 | 1.47-2.79 | 0.000 |
| Morphology | NSCLC |  |  |  |  |  |  | 1.98 | 1.27-3.08 | 0.003 | 1.40 | 0.92-2.12 | 0.120 |
|  | Unspecified |  |  |  |  |  |  | 0.98 | 0.59-1.63 | 0.945 | 0.87 | 0.54-1.41 | 0.581 |
| Stage at diagnosis | Unknown |  |  |  |  |  |  |  |  |  | 0.38 | 0.27-0.52 | 0.000 |
|  | Not advanced |  |  |  |  |  |  |  |  |  | 3.31 | 2.62-4.19 | 0.000 |

**Supplementary Table S12.** Modelled incidence rate ratios (IRRs) for pre-diagnostic healthcare use, by diagnostic route, with and without adjustment for socio-demographic and tumour factors **(-24 to -2 months)**.

# Primary care consultations (-24 to -2)

| **Variable** |  | **IRR** | **95% CI** | **p-value** | **IRR** | **95% CI** | **p-value** | **IRR** | **95% CI** | **p-value** | **IRR** | **95% CI** | **p-value** |
| --- | --- | --- | --- | --- | --- | --- | --- | --- | --- | --- | --- | --- | --- |
| Diagnostic route | GP referral | 1.12 | 1.05-1.19 | 0.001 | 1.31 | 1.23-1.39 | 0.000 | 1.31 | 1.23-1.4 | 0.000 | 1.30 | 1.22-1.38 | 0.000 |
|  | TWW | 0.88 | 0.82-0.93 | 0.000 | 1.12 | 1.06-1.19 | 0.000 | 1.12 | 1.05-1.19 | 0.000 | 1.12 | 1.05-1.19 | 0.000 |
|  | Other | 0.93 | 0.86-1 | 0.053 | 1.09 | 1.02-1.17 | 0.014 | 1.09 | 1.02-1.17 | 0.014 | 1.08 | 1-1.16 | 0.036 |
| Age at diagnosis | 85-99 |  |  |  | 1.07 | 0.99-1.15 | 0.070 | 1.07 | 0.99-1.15 | 0.071 | 1.07 | 0.99-1.15 | 0.082 |
|  | 30-64 |  |  |  | 0.72 | 0.67-0.76 | 0.000 | 0.72 | 0.67-0.76 | 0.000 | 0.72 | 0.67-0.77 | 0.000 |
|  | 65-74 |  |  |  | 0.88 | 0.83-0.93 | 0.000 | 0.88 | 0.83-0.93 | 0.000 | 0.88 | 0.83-0.93 | 0.000 |
| Gender | Female |  |  |  | 1.12 | 1.07-1.17 | 0.000 | 1.12 | 1.07-1.17 | 0.000 | 1.12 | 1.07-1.17 | 0.000 |
| Ethnicity | Non-White |  |  |  | 0.87 | 0.78-0.96 | 0.008 | 0.87 | 0.78-0.96 | 0.009 | 0.87 | 0.78-0.97 | 0.010 |
|  | Unknown |  |  |  | 0.76 | 0.64-0.9 | 0.001 | 0.76 | 0.64-0.9 | 0.001 | 0.76 | 0.64-0.89 | 0.001 |
| IMD | 2 |  |  |  | 0.94 | 0.87-1.01 | 0.073 | 0.94 | 0.87-1.01 | 0.073 | 0.93 | 0.87-1.01 | 0.071 |
|  | 3 |  |  |  | 1.00 | 0.93-1.07 | 0.996 | 1.00 | 0.93-1.07 | 1.000 | 1.00 | 0.93-1.07 | 0.983 |
|  | 4 |  |  |  | 0.91 | 0.84-0.97 | 0.007 | 0.91 | 0.84-0.97 | 0.007 | 0.91 | 0.85-0.98 | 0.008 |
|  | 5 - Most deprived |  |  |  | 0.88 | 0.82-0.95 | 0.001 | 0.88 | 0.82-0.95 | 0.001 | 0.88 | 0.82-0.95 | 0.001 |
| Smoker status | Non-smoker |  |  |  | 0.97 | 0.9-1.04 | 0.393 | 0.97 | 0.9-1.05 | 0.414 | 0.97 | 0.9-1.05 | 0.415 |
| COPD status | Pre-existing COPD (at -24 months or earlier) |  |  |  | 1.13 | 1.05-1.22 | 0.001 | 1.13 | 1.05-1.22 | 0.001 | 1.14 | 1.05-1.22 | 0.001 |
|  | No COPD |  |  |  | 0.85 | 0.79-0.9 | 0.000 | 0.84 | 0.79-0.9 | 0.000 | 0.85 | 0.79-0.91 | 0.000 |
| Elixhauser comorbidity score | 1 |  |  |  | 1.22 | 1.13-1.31 | 0.000 | 1.22 | 1.13-1.31 | 0.000 | 1.21 | 1.13-1.3 | 0.000 |
|  | 2 |  |  |  | 1.32 | 1.23-1.42 | 0.000 | 1.33 | 1.23-1.43 | 0.000 | 1.32 | 1.23-1.42 | 0.000 |
|  | 3+ |  |  |  | 1.73 | 1.63-1.85 | 0.000 | 1.73 | 1.63-1.85 | 0.000 | 1.73 | 1.63-1.85 | 0.000 |
| Morphology | NSCLC |  |  |  |  |  |  | 0.98 | 0.91-1.06 | 0.588 | 0.97 | 0.9-1.04 | 0.405 |
|  | Unspecified |  |  |  |  |  |  | 0.98 | 0.9-1.07 | 0.633 | 0.96 | 0.88-1.05 | 0.351 |
| Stage at diagnosis | Unknown |  |  |  |  |  |  |  |  |  | 1.09 | 1.04-1.14 | 0.001 |
|  | Not advanced |  |  |  |  |  |  |  |  |  | 1.09 | 1.02-1.17 | 0.008 |

# 6 selected relevant symptoms (-24 to -2)

| **Variable** |  | **IRR** | **95% CI** | **p-value** | **IRR** | **95% CI** | **p-value** | **IRR** | **95% CI** | **p-value** | **IRR** | **95% CI** | **p-value** |
| --- | --- | --- | --- | --- | --- | --- | --- | --- | --- | --- | --- | --- | --- |
| Diagnostic route | GP referral | 1.50 | 1.33-1.68 | 0.000 | 1.64 | 1.47-1.83 | 0.000 | 1.62 | 1.45-1.81 | 0.000 | 1.60 | 1.43-1.79 | 0.000 |
|  | TWW | 1.26 | 1.13-1.41 | 0.000 | 1.47 | 1.32-1.64 | 0.000 | 1.44 | 1.29-1.61 | 0.000 | 1.43 | 1.28-1.6 | 0.000 |
|  | Other | 1.21 | 1.06-1.38 | 0.004 | 1.33 | 1.17-1.51 | 0.000 | 1.31 | 1.16-1.49 | 0.000 | 1.30 | 1.14-1.47 | 0.000 |
| Age at diagnosis | 85-99 |  |  |  | 0.89 | 0.79-1.02 | 0.092 | 0.91 | 0.8-1.04 | 0.178 | 0.91 | 0.8-1.04 | 0.172 |
|  | 30-64 |  |  |  | 0.84 | 0.75-0.94 | 0.002 | 0.82 | 0.73-0.92 | 0.001 | 0.82 | 0.73-0.92 | 0.001 |
|  | 65-74 |  |  |  | 0.88 | 0.8-0.97 | 0.008 | 0.87 | 0.79-0.96 | 0.005 | 0.87 | 0.79-0.96 | 0.005 |
| Gender | Female |  |  |  | 1.08 | 1-1.17 | 0.046 | 1.08 | 1-1.17 | 0.046 | 1.08 | 1-1.17 | 0.048 |
| Ethnicity | Non-White |  |  |  | 0.88 | 0.72-1.07 | 0.197 | 0.88 | 0.72-1.08 | 0.224 | 0.89 | 0.72-1.08 | 0.241 |
|  | Unknown |  |  |  | 0.78 | 0.57-1.08 | 0.135 | 0.81 | 0.59-1.12 | 0.196 | 0.81 | 0.58-1.11 | 0.191 |
| IMD | 2 |  |  |  | 0.91 | 0.8-1.04 | 0.153 | 0.91 | 0.8-1.04 | 0.172 | 0.91 | 0.8-1.04 | 0.168 |
|  | 3 |  |  |  | 0.96 | 0.84-1.09 | 0.483 | 0.96 | 0.84-1.09 | 0.487 | 0.96 | 0.84-1.09 | 0.487 |
|  | 4 |  |  |  | 0.90 | 0.8-1.03 | 0.126 | 0.91 | 0.8-1.03 | 0.140 | 0.91 | 0.8-1.03 | 0.145 |
|  | 5 - Most deprived |  |  |  | 0.86 | 0.76-0.98 | 0.024 | 0.87 | 0.76-0.99 | 0.029 | 0.86 | 0.76-0.98 | 0.028 |
| Smoker status | Non-smoker |  |  |  | 0.92 | 0.79-1.07 | 0.277 | 0.92 | 0.79-1.08 | 0.304 | 0.92 | 0.79-1.08 | 0.318 |
| COPD status | Pre-existing COPD (at -24 months or earlier) |  |  |  | 1.57 | 1.39-1.77 | 0.000 | 1.58 | 1.4-1.78 | 0.000 | 1.58 | 1.4-1.78 | 0.000 |
|  | No COPD |  |  |  | 0.49 | 0.44-0.55 | 0.000 | 0.49 | 0.44-0.55 | 0.000 | 0.49 | 0.44-0.55 | 0.000 |
| Elixhauser comorbidity score | 1 |  |  |  | 1.19 | 1.04-1.36 | 0.010 | 1.19 | 1.05-1.36 | 0.008 | 1.19 | 1.04-1.35 | 0.011 |
|  | 2 |  |  |  | 1.09 | 0.96-1.25 | 0.197 | 1.09 | 0.96-1.25 | 0.188 | 1.09 | 0.95-1.25 | 0.217 |
|  | 3+ |  |  |  | 1.27 | 1.13-1.43 | 0.000 | 1.27 | 1.13-1.43 | 0.000 | 1.27 | 1.13-1.42 | 0.000 |
| Morphology | NSCLC |  |  |  |  |  |  | 0.92 | 0.81-1.05 | 0.228 | 0.91 | 0.79-1.04 | 0.155 |
|  | Unspecified |  |  |  |  |  |  | 0.86 | 0.74-1 | 0.044 | 0.84 | 0.72-0.98 | 0.026 |
| Stage at diagnosis | Unknown |  |  |  |  |  |  |  |  |  | 1.05 | 0.96-1.15 | 0.254 |
|  | Not advanced |  |  |  |  |  |  |  |  |  | 1.10 | 0.98-1.24 | 0.091 |

# 3 selected main respiratory symptoms (-24 to -2)

| **Variable** |  | **IRR** | **95% CI** | **p-value** | **IRR** | **95% CI** | **p-value** | **IRR** | **95% CI** | **p-value** | **IRR** | **95% CI** | **p-value** |
| --- | --- | --- | --- | --- | --- | --- | --- | --- | --- | --- | --- | --- | --- |
| Diagnostic route | GP referral | 1.52 | 1.34-1.71 | 0.00 | 1.66 | 1.48-1.87 | 0.000 | 1.64 | 1.46-1.85 | 0.000 | 1.62 | 1.44-1.83 | 0.000 |
|  | TWW | 1.27 | 1.13-1.43 | 0.00 | 1.49 | 1.33-1.67 | 0.000 | 1.46 | 1.3-1.64 | 0.000 | 1.45 | 1.29-1.64 | 0.000 |
|  | Other | 1.18 | 1.03-1.36 | 0.02 | 1.30 | 1.13-1.48 | 0.000 | 1.28 | 1.12-1.47 | 0.000 | 1.26 | 1.1-1.45 | 0.001 |
| Age at diagnosis | 85-99 |  |  |  | 0.90 | 0.78-1.03 | 0.125 | 0.92 | 0.8-1.05 | 0.222 | 0.91 | 0.79-1.05 | 0.209 |
|  | 30-64 |  |  |  | 0.86 | 0.76-0.97 | 0.018 | 0.85 | 0.75-0.96 | 0.009 | 0.85 | 0.75-0.96 | 0.010 |
|  | 65-74 |  |  |  | 0.92 | 0.83-1.02 | 0.105 | 0.91 | 0.82-1.01 | 0.075 | 0.91 | 0.82-1.01 | 0.079 |
| Gender | Female |  |  |  | 1.09 | 1-1.18 | 0.052 | 1.09 | 1-1.18 | 0.052 | 1.09 | 1-1.18 | 0.052 |
| Ethnicity | Non-White |  |  |  | 0.93 | 0.75-1.15 | 0.507 | 0.94 | 0.76-1.16 | 0.553 | 0.94 | 0.76-1.16 | 0.574 |
|  | Unknown |  |  |  | 0.77 | 0.54-1.08 | 0.131 | 0.79 | 0.56-1.12 | 0.185 | 0.79 | 0.55-1.11 | 0.177 |
| IMD | 2 |  |  |  | 0.90 | 0.78-1.03 | 0.129 | 0.90 | 0.78-1.04 | 0.144 | 0.90 | 0.78-1.04 | 0.141 |
|  | 3 |  |  |  | 0.93 | 0.81-1.07 | 0.316 | 0.93 | 0.81-1.07 | 0.319 | 0.93 | 0.81-1.07 | 0.314 |
|  | 4 |  |  |  | 0.89 | 0.77-1.02 | 0.088 | 0.89 | 0.78-1.02 | 0.097 | 0.89 | 0.78-1.02 | 0.101 |
|  | 5 - Most deprived |  |  |  | 0.82 | 0.71-0.94 | 0.004 | 0.82 | 0.71-0.94 | 0.005 | 0.82 | 0.71-0.94 | 0.004 |
| Smoker status | Non-smoker |  |  |  | 0.90 | 0.76-1.06 | 0.217 | 0.91 | 0.77-1.07 | 0.241 | 0.91 | 0.77-1.07 | 0.254 |
| COPD status | Pre-existing COPD (at -24 months or earlier) |  |  |  | 1.61 | 1.42-1.83 | 0.000 | 1.63 | 1.43-1.85 | 0.000 | 1.63 | 1.43-1.85 | 0.000 |
|  | No COPD |  |  |  | 0.44 | 0.39-0.5 | 0.000 | 0.44 | 0.39-0.5 | 0.000 | 0.44 | 0.39-0.5 | 0.000 |
| Elixhauser comorbidity score | 1 |  |  |  | 1.15 | 1-1.33 | 0.047 | 1.16 | 1.01-1.33 | 0.041 | 1.15 | 1-1.32 | 0.052 |
|  | 2 |  |  |  | 1.03 | 0.9-1.19 | 0.650 | 1.04 | 0.9-1.2 | 0.629 | 1.03 | 0.89-1.19 | 0.684 |
|  | 3+ |  |  |  | 1.23 | 1.09-1.4 | 0.001 | 1.24 | 1.09-1.4 | 0.001 | 1.23 | 1.09-1.4 | 0.001 |
| Morphology | NSCLC |  |  |  |  |  |  | 0.91 | 0.79-1.05 | 0.211 | 0.90 | 0.78-1.04 | 0.143 |
|  | Unspecified |  |  |  |  |  |  | 0.85 | 0.73-1 | 0.049 | 0.83 | 0.71-0.98 | 0.026 |
| Stage at diagnosis | Unknown |  |  |  |  |  |  |  |  |  | 1.08 | 0.98-1.19 | 0.119 |
|  | Not advanced |  |  |  |  |  |  |  |  |  | 1.11 | 0.99-1.26 | 0.084 |

# Cough consultations (-24 to -2)

| **Variable** |  | **IRR** | **95% CI** | **p-value** | **IRR** | **95% CI** | **p-value** | **IRR** | **95% CI** | **p-value** | **IRR** | **95% CI** | **p-value** |
| --- | --- | --- | --- | --- | --- | --- | --- | --- | --- | --- | --- | --- | --- |
| Diagnostic route | GP referral | 1.79 | 1.52-2.1 | 0.000 | 1.86 | 1.58-2.19 | 0.000 | 1.80 | 1.52-2.13 | 0.000 | 1.79 | 1.51-2.11 | 0.000 |
|  | TWW | 1.67 | 1.43-1.95 | 0.000 | 1.79 | 1.52-2.11 | 0.000 | 1.70 | 1.44-2.01 | 0.000 | 1.70 | 1.44-2.01 | 0.000 |
|  | Other | 1.26 | 1.04-1.52 | 0.018 | 1.31 | 1.08-1.59 | 0.005 | 1.27 | 1.05-1.54 | 0.014 | 1.26 | 1.04-1.52 | 0.021 |
| Age at diagnosis | 85-99 |  |  |  | 0.91 | 0.75-1.11 | 0.361 | 0.96 | 0.78-1.17 | 0.675 | 0.95 | 0.78-1.17 | 0.647 |
|  | 30-64 |  |  |  | 1.04 | 0.88-1.23 | 0.618 | 1.00 | 0.85-1.18 | 0.979 | 1.00 | 0.85-1.19 | 0.955 |
|  | 65-74 |  |  |  | 0.98 | 0.85-1.13 | 0.777 | 0.96 | 0.83-1.11 | 0.559 | 0.96 | 0.83-1.11 | 0.565 |
| Gender | Female |  |  |  | 1.21 | 1.07-1.36 | 0.002 | 1.21 | 1.08-1.36 | 0.001 | 1.21 | 1.08-1.36 | 0.001 |
| Ethnicity | Non-White |  |  |  | 0.96 | 0.72-1.28 | 0.803 | 0.98 | 0.74-1.31 | 0.905 | 0.99 | 0.74-1.32 | 0.927 |
|  | Unknown |  |  |  | 0.62 | 0.38-1.02 | 0.060 | 0.67 | 0.4-1.1 | 0.114 | 0.66 | 0.4-1.09 | 0.108 |
| IMD | 2 |  |  |  | 0.92 | 0.75-1.11 | 0.369 | 0.92 | 0.76-1.12 | 0.423 | 0.92 | 0.76-1.12 | 0.423 |
|  | 3 |  |  |  | 0.96 | 0.8-1.17 | 0.707 | 0.97 | 0.8-1.17 | 0.733 | 0.97 | 0.8-1.17 | 0.722 |
|  | 4 |  |  |  | 0.93 | 0.77-1.12 | 0.433 | 0.94 | 0.77-1.13 | 0.485 | 0.94 | 0.78-1.13 | 0.505 |
|  | 5 - Most deprived |  |  |  | 0.78 | 0.64-0.95 | 0.012 | 0.79 | 0.65-0.96 | 0.016 | 0.79 | 0.65-0.95 | 0.015 |
| Smoker status | Non-smoker |  |  |  | 0.97 | 0.78-1.2 | 0.761 | 0.97 | 0.78-1.21 | 0.800 | 0.97 | 0.78-1.21 | 0.816 |
| COPD status | Pre-existing COPD (at -24 months or earlier) |  |  |  | 1.16 | 0.96-1.39 | 0.132 | 1.18 | 0.97-1.42 | 0.090 | 1.18 | 0.98-1.42 | 0.086 |
|  | No COPD |  |  |  | 0.59 | 0.5-0.71 | 0.000 | 0.59 | 0.5-0.7 | 0.000 | 0.59 | 0.5-0.71 | 0.000 |
| Elixhauser comorbidity score | 1 |  |  |  | 1.14 | 0.95-1.38 | 0.165 | 1.15 | 0.95-1.39 | 0.151 | 1.14 | 0.94-1.38 | 0.171 |
|  | 2 |  |  |  | 1.06 | 0.87-1.28 | 0.574 | 1.06 | 0.87-1.28 | 0.567 | 1.05 | 0.87-1.28 | 0.591 |
|  | 3+ |  |  |  | 1.13 | 0.96-1.34 | 0.150 | 1.13 | 0.96-1.34 | 0.152 | 1.13 | 0.96-1.34 | 0.147 |
| Morphology | NSCLC |  |  |  |  |  |  | 0.88 | 0.73-1.07 | 0.200 | 0.87 | 0.72-1.06 | 0.159 |
|  | Unspecified |  |  |  |  |  |  | 0.73 | 0.58-0.91 | 0.006 | 0.71 | 0.57-0.89 | 0.003 |
| Stage at diagnosis | Unknown |  |  |  |  |  |  |  |  |  | 1.13 | 0.99-1.29 | 0.073 |
|  | Not advanced |  |  |  |  |  |  |  |  |  | 1.11 | 0.93-1.31 | 0.251 |

# Dyspnoea consultations (-24 to -2)

| **Variable** |  | **IRR** | **95% CI** | **p-value** | **IRR** | **95% CI** | **p-value** | **IRR** | **95% CI** | **p-value** | **IRR** | **95% CI** | **p-value** |
| --- | --- | --- | --- | --- | --- | --- | --- | --- | --- | --- | --- | --- | --- |
| Diagnostic route | GP referral | 1.12 | 0.94-1.34 | 0.215 | 1.29 | 1.1-1.51 | 0.002 | 1.31 | 1.12-1.54 | 0.001 | 1.30 | 1.1-1.53 | 0.002 |
|  | TWW | 0.82 | 0.69-0.98 | 0.028 | 1.03 | 0.88-1.21 | 0.701 | 1.05 | 0.89-1.24 | 0.540 | 1.04 | 0.88-1.23 | 0.605 |
|  | Other | 1.04 | 0.85-1.28 | 0.700 | 1.18 | 0.98-1.41 | 0.074 | 1.19 | 1-1.43 | 0.055 | 1.18 | 0.98-1.42 | 0.076 |
| Age at diagnosis | 85-99 |  |  |  | 0.88 | 0.73-1.06 | 0.188 | 0.87 | 0.72-1.05 | 0.147 | 0.87 | 0.72-1.05 | 0.148 |
|  | 30-64 |  |  |  | 0.65 | 0.54-0.78 | 0.000 | 0.66 | 0.55-0.8 | 0.000 | 0.66 | 0.55-0.8 | 0.000 |
|  | 65-74 |  |  |  | 0.88 | 0.77-1.01 | 0.075 | 0.89 | 0.77-1.03 | 0.107 | 0.89 | 0.78-1.03 | 0.112 |
| Gender | Female |  |  |  | 0.97 | 0.86-1.09 | 0.583 | 0.96 | 0.86-1.08 | 0.542 | 0.96 | 0.86-1.08 | 0.535 |
| Ethnicity | Non-White |  |  |  | 0.81 | 0.58-1.13 | 0.211 | 0.80 | 0.58-1.12 | 0.195 | 0.81 | 0.58-1.12 | 0.203 |
|  | Unknown |  |  |  | 1.09 | 0.68-1.74 | 0.722 | 1.06 | 0.66-1.7 | 0.804 | 1.06 | 0.66-1.7 | 0.810 |
| IMD | 2 |  |  |  | 0.87 | 0.72-1.07 | 0.184 | 0.87 | 0.72-1.06 | 0.172 | 0.87 | 0.71-1.06 | 0.167 |
|  | 3 |  |  |  | 0.91 | 0.75-1.11 | 0.352 | 0.91 | 0.75-1.1 | 0.340 | 0.91 | 0.75-1.1 | 0.342 |
|  | 4 |  |  |  | 0.84 | 0.7-1.02 | 0.085 | 0.84 | 0.69-1.02 | 0.077 | 0.84 | 0.69-1.02 | 0.077 |
|  | 5 - Most deprived |  |  |  | 0.85 | 0.7-1.03 | 0.100 | 0.85 | 0.7-1.02 | 0.086 | 0.84 | 0.7-1.02 | 0.085 |
| Smoker status | Non-smoker |  |  |  | 0.72 | 0.54-0.96 | 0.025 | 0.72 | 0.54-0.96 | 0.028 | 0.72 | 0.54-0.97 | 0.028 |
| COPD status | Pre-existing COPD (at -24 months or earlier) |  |  |  | 2.11 | 1.8-2.47 | 0.000 | 2.10 | 1.79-2.46 | 0.000 | 2.09 | 1.78-2.45 | 0.000 |
|  | No COPD |  |  |  | 0.25 | 0.21-0.3 | 0.000 | 0.25 | 0.21-0.3 | 0.000 | 0.25 | 0.21-0.3 | 0.000 |
| Elixhauser comorbidity score | 1 |  |  |  | 1.13 | 0.91-1.4 | 0.278 | 1.13 | 0.91-1.4 | 0.272 | 1.12 | 0.9-1.4 | 0.297 |
|  | 2 |  |  |  | 1.07 | 0.86-1.33 | 0.549 | 1.07 | 0.86-1.33 | 0.540 | 1.06 | 0.85-1.32 | 0.585 |
|  | 3+ |  |  |  | 1.38 | 1.15-1.65 | 0.001 | 1.38 | 1.15-1.66 | 0.001 | 1.37 | 1.15-1.65 | 0.001 |
| Morphology | NSCLC |  |  |  |  |  |  | 0.93 | 0.76-1.14 | 0.465 | 0.91 | 0.75-1.12 | 0.389 |
|  | Unspecified |  |  |  |  |  |  | 1.01 | 0.81-1.26 | 0.942 | 1.00 | 0.8-1.24 | 0.971 |
| Stage at diagnosis | Unknown |  |  |  |  |  |  |  |  |  | 1.01 | 0.89-1.16 | 0.856 |
|  | Not advanced |  |  |  |  |  |  |  |  |  | 1.09 | 0.92-1.28 | 0.312 |

# Haemoptysis consultations (-24 to -2)

| **Variable** |  | **IRR** | **95% CI** | **p-value** | **IRR** | **95% CI** | **p-value** | **IRR** | **95% CI** | **p-value** | **IRR** | **95% CI** | **p-value** |
| --- | --- | --- | --- | --- | --- | --- | --- | --- | --- | --- | --- | --- | --- |
| Diagnostic route | GP referral | 3.38 | 1.18-9.69 | 0.023 | 4.01 | 1.35-11.88 | 0.012 | 3.96 | 1.31-11.93 | 0.014 | 3.33 | 0.19-59.64 | 0.414 |
|  | TWW | 2.34 | 0.8-6.83 | 0.120 | 2.94 | 0.96-9.06 | 0.060 | 2.92 | 0.92-9.25 | 0.069 | 2.50 | 0.12-53.39 | 0.558 |
|  | Other | 1.61 | 0.42-6.15 | 0.488 | 1.95 | 0.5-7.52 | 0.333 | 1.93 | 0.5-7.52 | 0.343 | 1.55 | 0.05-53.08 | 0.808 |
| Age at diagnosis | 85-99 |  |  |  | 1.11 | 0.34-3.61 | 0.858 | 1.12 | 0.34-3.7 | 0.855 | 1.02 | 0.05-20.92 | 0.988 |
|  | 30-64 |  |  |  | 0.89 | 0.32-2.47 | 0.816 | 0.88 | 0.31-2.49 | 0.811 | 0.79 | 0.06-10.47 | 0.861 |
|  | 65-74 |  |  |  | 0.74 | 0.3-1.84 | 0.521 | 0.74 | 0.3-1.84 | 0.512 | 0.65 | 0.07-6.28 | 0.710 |
| Gender | Female |  |  |  | 0.69 | 0.32-1.47 | 0.334 | 0.69 | 0.32-1.47 | 0.336 | 0.70 | 0.1-4.84 | 0.714 |
| Ethnicity | Non-White |  |  |  | 0.85 | 0.12-6.19 | 0.877 | 0.86 | 0.12-6.26 | 0.880 | 0.84 | 0.01-137.97 | 0.945 |
|  | Unknown |  |  |  | 0.43 | 0.01-30.54 | 0.696 | 0.43 | 0.01-31.39 | 0.699 | 0.72 | 0-19848.85 | 0.949 |
| IMD | 2 |  |  |  | 1.16 | 0.33-4.05 | 0.816 | 1.17 | 0.33-4.08 | 0.808 | 1.11 | 0.04-28.07 | 0.951 |
|  | 3 |  |  |  | 1.34 | 0.4-4.48 | 0.639 | 1.34 | 0.4-4.52 | 0.633 | 1.27 | 0.06-25.82 | 0.877 |
|  | 4 |  |  |  | 1.01 | 0.29-3.56 | 0.990 | 1.02 | 0.29-3.59 | 0.980 | 0.98 | 0.04-24.01 | 0.991 |
|  | 5 - Most deprived |  |  |  | 1.17 | 0.34-4.05 | 0.804 | 1.18 | 0.34-4.11 | 0.792 | 1.08 | 0.05-24.83 | 0.960 |
| Smoker status | Non-smoker |  |  |  | 0.94 | 0.21-4.15 | 0.940 | 0.93 | 0.21-4.1 | 0.922 | 0.78 | 0.01-47.38 | 0.907 |
| COPD status | Pre-existing COPD (at -24 months or earlier) |  |  |  | 0.97 | 0.33-2.84 | 0.950 | 0.96 | 0.33-2.84 | 0.947 | 0.93 | 0.06-13.46 | 0.957 |
|  | No COPD |  |  |  | 0.56 | 0.2-1.55 | 0.264 | 0.56 | 0.2-1.55 | 0.264 | 0.53 | 0.04-7.29 | 0.632 |
| Elixhauser comorbidity score | 1 |  |  |  | 1.50 | 0.44-5.1 | 0.521 | 1.48 | 0.43-5.05 | 0.530 | 1.29 | 0.06-29.3 | 0.871 |
|  | 2 |  |  |  | 1.20 | 0.32-4.46 | 0.781 | 1.19 | 0.32-4.44 | 0.792 | 1.03 | 0.04-29.74 | 0.984 |
|  | 3+ |  |  |  | 1.72 | 0.57-5.17 | 0.335 | 1.71 | 0.57-5.14 | 0.340 | 1.50 | 0.09-24.88 | 0.778 |
| Morphology | NSCLC |  |  |  |  |  |  | 1.25 | 0.33-4.78 | 0.747 | 0.99 | 0.03-28.52 | 0.994 |
|  | Unspecified |  |  |  |  |  |  | 1.17 | 0.26-5.34 | 0.836 | 0.83 | 0.02-38.98 | 0.923 |
| Stage at diagnosis | Unknown |  |  |  |  |  |  |  |  |  | 1.36 | 0.17-10.73 | 0.767 |
|  | Not advanced |  |  |  |  |  |  |  |  |  | 1.16 | 0.09-14.54 | 0.908 |

# Any chest imaging event (-24 to -2)

| **Variable** |  | **IRR** | **95% CI** | **p-value** | **IRR** | **95% CI** | **p-value** | **IRR** | **95% CI** | **p-value** | **IRR** | **95% CI** | **p-value** |
| --- | --- | --- | --- | --- | --- | --- | --- | --- | --- | --- | --- | --- | --- |
| Diagnostic route | GP referral | 1.95 | 1.57-2.42 | 0.000 | 2.49 | 2-3.1 | 0.000 | 2.35 | 1.88-2.93 | 0.000 | 1.88 | 1.52-2.34 | 0.000 |
|  | TWW | 1.26 | 1.02-1.55 | 0.036 | 1.83 | 1.46-2.28 | 0.000 | 1.70 | 1.36-2.13 | 0.000 | 1.28 | 1.03-1.6 | 0.027 |
|  | Other | 1.39 | 1.08-1.78 | 0.011 | 1.70 | 1.33-2.19 | 0.000 | 1.61 | 1.25-2.08 | 0.000 | 1.35 | 1.05-1.72 | 0.017 |
| Age at diagnosis | 85-99 |  |  |  | 1.16 | 0.91-1.5 | 0.237 | 1.24 | 0.96-1.6 | 0.099 | 1.28 | 1-1.64 | 0.048 |
|  | 30-64 |  |  |  | 0.88 | 0.7-1.1 | 0.261 | 0.84 | 0.67-1.05 | 0.129 | 0.83 | 0.66-1.03 | 0.093 |
|  | 65-74 |  |  |  | 0.87 | 0.71-1.06 | 0.157 | 0.84 | 0.69-1.02 | 0.081 | 0.85 | 0.7-1.03 | 0.104 |
| Gender | Female |  |  |  | 1.16 | 0.99-1.36 | 0.069 | 1.17 | 1-1.37 | 0.054 | 1.11 | 0.95-1.29 | 0.186 |
| Ethnicity | Non-White |  |  |  | 0.65 | 0.43-0.98 | 0.040 | 0.67 | 0.44-1.01 | 0.056 | 0.70 | 0.46-1.05 | 0.082 |
|  | Unknown |  |  |  | 1.09 | 0.61-1.95 | 0.782 | 1.21 | 0.67-2.18 | 0.527 | 1.39 | 0.78-2.46 | 0.261 |
| IMD | 2 |  |  |  | 0.88 | 0.68-1.14 | 0.340 | 0.90 | 0.69-1.16 | 0.405 | 0.88 | 0.69-1.14 | 0.340 |
|  | 3 |  |  |  | 0.88 | 0.68-1.13 | 0.306 | 0.88 | 0.68-1.14 | 0.341 | 0.89 | 0.7-1.14 | 0.357 |
|  | 4 |  |  |  | 0.94 | 0.73-1.21 | 0.604 | 0.95 | 0.74-1.23 | 0.704 | 0.95 | 0.75-1.22 | 0.692 |
|  | 5 - Most deprived |  |  |  | 0.85 | 0.66-1.1 | 0.223 | 0.88 | 0.68-1.14 | 0.319 | 0.90 | 0.7-1.16 | 0.424 |
| Smoker status | Non-smoker |  |  |  | 1.33 | 1.01-1.75 | 0.039 | 1.31 | 1-1.73 | 0.052 | 1.43 | 1.09-1.86 | 0.009 |
| COPD status | Pre-existing COPD (at -24 months or earlier) |  |  |  | 1.00 | 0.77-1.28 | 0.978 | 1.02 | 0.79-1.31 | 0.890 | 0.90 | 0.71-1.15 | 0.407 |
|  | No COPD |  |  |  | 0.56 | 0.45-0.71 | 0.000 | 0.56 | 0.44-0.7 | 0.000 | 0.56 | 0.45-0.7 | 0.000 |
| Elixhauser comorbidity score | 1 |  |  |  | 1.39 | 1.06-1.81 | 0.016 | 1.37 | 1.05-1.79 | 0.020 | 1.28 | 0.98-1.66 | 0.066 |
|  | 2 |  |  |  | 1.28 | 0.97-1.68 | 0.077 | 1.27 | 0.96-1.66 | 0.090 | 1.15 | 0.88-1.5 | 0.312 |
|  | 3+ |  |  |  | 2.37 | 1.88-3 | 0.000 | 2.36 | 1.86-2.98 | 0.000 | 2.14 | 1.71-2.69 | 0.000 |
| Morphology | NSCLC |  |  |  |  |  |  | 1.25 | 0.95-1.65 | 0.113 | 1.02 | 0.78-1.33 | 0.903 |
|  | Unspecified |  |  |  |  |  |  | 0.92 | 0.67-1.26 | 0.595 | 1.02 | 0.75-1.38 | 0.916 |
| Stage at diagnosis | Unknown |  |  |  |  |  |  |  |  |  | 0.23 | 0.19-0.28 | 0.000 |
|  | Not advanced |  |  |  |  |  |  |  |  |  | 2.36 | 1.95-2.87 | 0.000 |

# Chest X-rays (-24 to -2)

| **Variable** |  | **IRR** | **95% CI** | **p-value** | **IRR** | **95% CI** | **p-value** | **IRR** | **95% CI** | **p-value** | **IRR** | **95% CI** | **p-value** |
| --- | --- | --- | --- | --- | --- | --- | --- | --- | --- | --- | --- | --- | --- |
| Diagnostic route | GP referral | 1.55 | 1.25-1.92 | 0.000 | 1.99 | 1.6-2.46 | 0.000 | 1.92 | 1.54-2.38 | 0.000 | 1.62 | 1.31-2.01 | 0.000 |
|  | TWW | 1.15 | 0.94-1.42 | 0.174 | 1.67 | 1.34-2.07 | 0.000 | 1.59 | 1.27-1.98 | 0.000 | 1.26 | 1.01-1.57 | 0.037 |
|  | Other | 1.18 | 0.93-1.51 | 0.180 | 1.45 | 1.14-1.85 | 0.003 | 1.40 | 1.1-1.8 | 0.007 | 1.23 | 0.96-1.58 | 0.096 |
| Age at diagnosis | 85-99 |  |  |  | 1.23 | 0.96-1.56 | 0.096 | 1.28 | 1-1.64 | 0.048 | 1.33 | 1.04-1.69 | 0.023 |
|  | 30-64 |  |  |  | 0.90 | 0.72-1.12 | 0.348 | 0.87 | 0.7-1.09 | 0.224 | 0.86 | 0.69-1.07 | 0.175 |
|  | 65-74 |  |  |  | 0.84 | 0.69-1.02 | 0.083 | 0.82 | 0.68-1 | 0.052 | 0.83 | 0.69-1.01 | 0.063 |
| Gender | Female |  |  |  | 1.13 | 0.97-1.32 | 0.113 | 1.14 | 0.98-1.33 | 0.097 | 1.09 | 0.94-1.27 | 0.255 |
| Ethnicity | Non-White |  |  |  | 0.64 | 0.42-0.97 | 0.036 | 0.65 | 0.43-0.99 | 0.045 | 0.68 | 0.45-1.03 | 0.069 |
|  | Unknown |  |  |  | 1.19 | 0.68-2.09 | 0.545 | 1.28 | 0.72-2.25 | 0.397 | 1.48 | 0.85-2.59 | 0.169 |
| IMD | 2 |  |  |  | 0.89 | 0.69-1.15 | 0.384 | 0.90 | 0.7-1.16 | 0.427 | 0.89 | 0.69-1.15 | 0.368 |
|  | 3 |  |  |  | 0.86 | 0.67-1.11 | 0.253 | 0.87 | 0.67-1.12 | 0.268 | 0.87 | 0.68-1.11 | 0.272 |
|  | 4 |  |  |  | 0.91 | 0.71-1.16 | 0.441 | 0.92 | 0.71-1.17 | 0.487 | 0.91 | 0.71-1.17 | 0.466 |
|  | 5 - Most deprived |  |  |  | 0.90 | 0.7-1.16 | 0.411 | 0.91 | 0.71-1.18 | 0.488 | 0.94 | 0.73-1.2 | 0.616 |
| Smoker status | Non-smoker |  |  |  | 1.28 | 0.98-1.68 | 0.073 | 1.27 | 0.97-1.67 | 0.081 | 1.37 | 1.05-1.79 | 0.019 |
| COPD status | Pre-existing COPD (at -24 months or earlier) |  |  |  | 0.99 | 0.77-1.26 | 0.925 | 1.00 | 0.78-1.28 | 0.983 | 0.90 | 0.71-1.14 | 0.376 |
|  | No COPD |  |  |  | 0.56 | 0.44-0.69 | 0.000 | 0.55 | 0.44-0.69 | 0.000 | 0.54 | 0.44-0.68 | 0.000 |
| Elixhauser comorbidity score | 1 |  |  |  | 1.24 | 0.95-1.62 | 0.107 | 1.24 | 0.95-1.61 | 0.116 | 1.17 | 0.9-1.52 | 0.241 |
|  | 2 |  |  |  | 1.13 | 0.86-1.48 | 0.384 | 1.12 | 0.86-1.47 | 0.406 | 1.04 | 0.79-1.36 | 0.786 |
|  | 3+ |  |  |  | 2.20 | 1.75-2.76 | 0.000 | 2.19 | 1.74-2.75 | 0.000 | 2.03 | 1.62-2.54 | 0.000 |
| Morphology | NSCLC |  |  |  |  |  |  | 1.08 | 0.83-1.41 | 0.560 | 0.94 | 0.72-1.23 | 0.644 |
|  | Unspecified |  |  |  |  |  |  | 0.89 | 0.66-1.2 | 0.451 | 1.01 | 0.75-1.37 | 0.936 |
| Stage at diagnosis | Unknown |  |  |  |  |  |  |  |  |  | 0.24 | 0.2-0.3 | 0.000 |
|  | Not advanced |  |  |  |  |  |  |  |  |  | 1.82 | 1.5-2.21 | 0.000 |

# Chest CT scans (-24 to -2)

| **Variable** |  | **IRR** | **95% CI** | **p-value** | **IRR** | **95% CI** | **p-value** | **IRR** | **95% CI** | **p-value** | **IRR** | **95% CI** | **p-value** |
| --- | --- | --- | --- | --- | --- | --- | --- | --- | --- | --- | --- | --- | --- |
| Diagnostic route | GP referral | 2.99 | 2.15-4.16 | 0.000 | 3.59 | 2.59-4.98 | 0.000 | 3.18 | 2.29-4.4 | 0.000 | 2.38 | 1.75-3.24 | 0.000 |
|  | TWW | 1.48 | 1.05-2.08 | 0.024 | 1.97 | 1.39-2.8 | 0.000 | 1.71 | 1.21-2.43 | 0.003 | 1.23 | 0.88-1.72 | 0.230 |
|  | Other | 2.31 | 1.59-3.35 | 0.000 | 2.68 | 1.85-3.86 | 0.000 | 2.40 | 1.66-3.46 | 0.000 | 1.77 | 1.25-2.51 | 0.001 |
| Age at diagnosis | 85-99 |  |  |  | 0.87 | 0.59-1.29 | 0.483 | 0.97 | 0.66-1.44 | 0.889 | 0.98 | 0.68-1.42 | 0.911 |
|  | 30-64 |  |  |  | 0.86 | 0.61-1.2 | 0.378 | 0.79 | 0.57-1.11 | 0.175 | 0.78 | 0.56-1.07 | 0.117 |
|  | 65-74 |  |  |  | 1.00 | 0.75-1.33 | 0.984 | 0.94 | 0.71-1.25 | 0.679 | 0.97 | 0.75-1.27 | 0.838 |
| Gender | Female |  |  |  | 1.18 | 0.93-1.49 | 0.168 | 1.20 | 0.95-1.52 | 0.122 | 1.13 | 0.91-1.41 | 0.273 |
| Ethnicity | Non-White |  |  |  | 0.70 | 0.37-1.34 | 0.281 | 0.74 | 0.39-1.4 | 0.348 | 0.78 | 0.42-1.45 | 0.429 |
|  | Unknown |  |  |  | 0.87 | 0.34-2.2 | 0.763 | 1.09 | 0.43-2.75 | 0.851 | 1.09 | 0.45-2.66 | 0.852 |
| IMD | 2 |  |  |  | 1.02 | 0.7-1.5 | 0.911 | 1.05 | 0.72-1.54 | 0.789 | 1.02 | 0.72-1.46 | 0.898 |
|  | 3 |  |  |  | 0.99 | 0.67-1.44 | 0.942 | 1.01 | 0.69-1.47 | 0.966 | 1.00 | 0.7-1.43 | 0.990 |
|  | 4 |  |  |  | 1.13 | 0.78-1.64 | 0.516 | 1.18 | 0.81-1.7 | 0.386 | 1.17 | 0.83-1.66 | 0.372 |
|  | 5 - Most deprived |  |  |  | 0.82 | 0.56-1.22 | 0.327 | 0.87 | 0.59-1.29 | 0.492 | 0.86 | 0.6-1.25 | 0.433 |
| Smoker status | Non-smoker |  |  |  | 1.35 | 0.9-2.02 | 0.142 | 1.30 | 0.87-1.93 | 0.198 | 1.39 | 0.96-2.03 | 0.081 |
| COPD status | Pre-existing COPD (at -24 months or earlier) |  |  |  | 0.83 | 0.58-1.2 | 0.326 | 0.87 | 0.6-1.24 | 0.439 | 0.80 | 0.57-1.12 | 0.188 |
|  | No COPD |  |  |  | 0.62 | 0.44-0.86 | 0.004 | 0.61 | 0.44-0.84 | 0.003 | 0.64 | 0.47-0.87 | 0.004 |
| Elixhauser comorbidity score | 1 |  |  |  | 1.71 | 1.14-2.56 | 0.010 | 1.66 | 1.11-2.48 | 0.014 | 1.49 | 1.02-2.19 | 0.041 |
|  | 2 |  |  |  | 1.72 | 1.14-2.6 | 0.010 | 1.69 | 1.12-2.54 | 0.012 | 1.47 | 1-2.17 | 0.052 |
|  | 3+ |  |  |  | 2.51 | 1.75-3.6 | 0.000 | 2.47 | 1.73-3.53 | 0.000 | 2.21 | 1.58-3.1 | 0.000 |
| Morphology | NSCLC |  |  |  |  |  |  | 2.05 | 1.29-3.25 | 0.002 | 1.44 | 0.93-2.24 | 0.101 |
|  | Unspecified |  |  |  |  |  |  | 1.10 | 0.66-1.86 | 0.710 | 1.01 | 0.62-1.65 | 0.976 |
| Stage at diagnosis | Unknown |  |  |  |  |  |  |  |  |  | 0.40 | 0.29-0.55 | 0.000 |
|  | Not advanced |  |  |  |  |  |  |  |  |  | 3.65 | 2.84-4.69 | 0.000 |

**Supplementary Table S13.** Modelled incidence rate ratios (IRRs) for pre-diagnostic healthcare use, by diagnostic route, with and without adjustment for socio-demographic and tumour factors **(-24 to -6 months)**.

# Primary care consultations (-24 to -6 months)

| **Variable** |  | **IRR** | **95% CI** | **p-value** | **IRR** | **95% CI** | **p-value** | **IRR** | **95% CI** | **p-value** | **IRR** | **95% CI** | **p-value** |
| --- | --- | --- | --- | --- | --- | --- | --- | --- | --- | --- | --- | --- | --- |
| Diagnostic route | GP referral | 1.10 | 1.02-1.18 | 0.014 | 1.30 | 1.22-1.39 | 0.000 | 1.30 | 1.22-1.4 | 0.000 | 1.29 | 1.2-1.38 | 0.000 |
|  | TWW | 0.84 | 0.79-0.9 | 0.000 | 1.11 | 1.04-1.19 | 0.002 | 1.11 | 1.03-1.19 | 0.003 | 1.10 | 1.03-1.18 | 0.005 |
|  | Other | 0.94 | 0.86-1.02 | 0.136 | 1.12 | 1.04-1.21 | 0.004 | 1.12 | 1.04-1.21 | 0.004 | 1.10 | 1.02-1.19 | 0.015 |
| Age at diagnosis | 85-99 |  |  |  | 1.06 | 0.98-1.15 | 0.138 | 1.06 | 0.98-1.15 | 0.136 | 1.06 | 0.98-1.15 | 0.155 |
|  | 30-64 |  |  |  | 0.66 | 0.61-0.7 | 0.000 | 0.65 | 0.61-0.7 | 0.000 | 0.66 | 0.61-0.71 | 0.000 |
|  | 65-74 |  |  |  | 0.84 | 0.79-0.9 | 0.000 | 0.84 | 0.79-0.9 | 0.000 | 0.84 | 0.79-0.9 | 0.000 |
| Gender | Female |  |  |  | 1.13 | 1.08-1.19 | 0.000 | 1.13 | 1.08-1.19 | 0.000 | 1.13 | 1.08-1.19 | 0.000 |
| Ethnicity | Non-White |  |  |  | 0.86 | 0.77-0.97 | 0.015 | 0.86 | 0.77-0.97 | 0.015 | 0.87 | 0.77-0.98 | 0.019 |
|  | Unknown |  |  |  | 0.76 | 0.63-0.91 | 0.003 | 0.76 | 0.63-0.91 | 0.003 | 0.75 | 0.63-0.91 | 0.003 |
| IMD | 2 |  |  |  | 0.94 | 0.87-1.02 | 0.147 | 0.94 | 0.87-1.02 | 0.148 | 0.94 | 0.87-1.02 | 0.142 |
|  | 3 |  |  |  | 0.99 | 0.92-1.08 | 0.887 | 0.99 | 0.92-1.08 | 0.886 | 0.99 | 0.92-1.08 | 0.868 |
|  | 4 |  |  |  | 0.91 | 0.84-0.98 | 0.017 | 0.91 | 0.84-0.98 | 0.018 | 0.91 | 0.84-0.99 | 0.020 |
|  | 5 - Most deprived |  |  |  | 0.90 | 0.83-0.98 | 0.013 | 0.90 | 0.83-0.98 | 0.013 | 0.90 | 0.83-0.98 | 0.010 |
| Smoker status | Non-smoker |  |  |  | 0.96 | 0.88-1.05 | 0.386 | 0.96 | 0.88-1.05 | 0.394 | 0.96 | 0.88-1.05 | 0.389 |
| COPD status | Pre-existing COPD (at -24 months or earlier) |  |  |  | 1.19 | 1.1-1.3 | 0.000 | 1.19 | 1.1-1.3 | 0.000 | 1.20 | 1.1-1.3 | 0.000 |
|  | No COPD |  |  |  | 0.84 | 0.78-0.91 | 0.000 | 0.84 | 0.78-0.91 | 0.000 | 0.85 | 0.79-0.91 | 0.000 |
| Elixhauser comorbidity score | 1 |  |  |  | 1.25 | 1.15-1.35 | 0.000 | 1.25 | 1.15-1.35 | 0.000 | 1.24 | 1.14-1.34 | 0.000 |
|  | 2 |  |  |  | 1.36 | 1.26-1.48 | 0.000 | 1.36 | 1.26-1.48 | 0.000 | 1.36 | 1.25-1.47 | 0.000 |
|  | 3+ |  |  |  | 1.81 | 1.69-1.95 | 0.000 | 1.82 | 1.69-1.95 | 0.000 | 1.81 | 1.69-1.95 | 0.000 |
| Morphology | NSCLC |  |  |  |  |  |  | 0.99 | 0.91-1.08 | 0.823 | 0.98 | 0.9-1.06 | 0.567 |
|  | Unspecified |  |  |  |  |  |  | 0.99 | 0.9-1.08 | 0.783 | 0.96 | 0.88-1.06 | 0.443 |
| Stage at diagnosis | Unknown |  |  |  |  |  |  |  |  |  | 1.10 | 1.04-1.16 | 0.001 |
|  | Not advanced |  |  |  |  |  |  |  |  |  | 1.12 | 1.04-1.21 | 0.002 |

# 6 selected relevant symptoms (-24 to -6)

| **Variable** |  | **IRR** | **95% CI** | **p-value** | **IRR** | **95% CI** | **p-value** | **IRR** | **95% CI** | **p-value** | **IRR** | **95% CI** | **p-value** |
| --- | --- | --- | --- | --- | --- | --- | --- | --- | --- | --- | --- | --- | --- |
| Diagnostic route | GP referral | 1.37 | 1.19-1.58 | 0.000 | 1.57 | 1.38-1.79 | 0.000 | 1.56 | 1.37-1.79 | 0.000 | 1.53 | 1.34-1.75 | 0.000 |
|  | TWW | 1.01 | 0.88-1.16 | 0.870 | 1.27 | 1.11-1.45 | 0.000 | 1.26 | 1.1-1.45 | 0.001 | 1.25 | 1.09-1.43 | 0.002 |
|  | Other | 1.21 | 1.03-1.42 | 0.023 | 1.37 | 1.17-1.59 | 0.000 | 1.36 | 1.17-1.58 | 0.000 | 1.33 | 1.14-1.55 | 0.000 |
| Age at diagnosis | 85-99 |  |  |  | 0.85 | 0.73-1 | 0.049 | 0.86 | 0.73-1.01 | 0.059 | 0.86 | 0.73-1 | 0.056 |
|  | 30-64 |  |  |  | 0.75 | 0.65-0.86 | 0.000 | 0.75 | 0.65-0.86 | 0.000 | 0.75 | 0.65-0.86 | 0.000 |
|  | 65-74 |  |  |  | 0.81 | 0.72-0.91 | 0.001 | 0.81 | 0.72-0.91 | 0.001 | 0.81 | 0.72-0.91 | 0.001 |
| Gender | Female |  |  |  | 1.15 | 1.04-1.27 | 0.005 | 1.15 | 1.04-1.27 | 0.005 | 1.15 | 1.04-1.26 | 0.006 |
| Ethnicity | Non-White |  |  |  | 0.77 | 0.59-1 | 0.054 | 0.77 | 0.6-1.01 | 0.057 | 0.78 | 0.6-1.02 | 0.068 |
|  | Unknown |  |  |  | 0.74 | 0.49-1.11 | 0.148 | 0.74 | 0.49-1.12 | 0.159 | 0.74 | 0.49-1.12 | 0.157 |
| IMD | 2 |  |  |  | 0.91 | 0.78-1.07 | 0.261 | 0.91 | 0.78-1.07 | 0.266 | 0.91 | 0.77-1.07 | 0.256 |
|  | 3 |  |  |  | 0.94 | 0.8-1.1 | 0.417 | 0.94 | 0.8-1.1 | 0.423 | 0.94 | 0.8-1.1 | 0.427 |
|  | 4 |  |  |  | 0.92 | 0.79-1.08 | 0.304 | 0.92 | 0.79-1.08 | 0.313 | 0.92 | 0.79-1.08 | 0.323 |
|  | 5 - Most deprived |  |  |  | 0.85 | 0.73-1 | 0.051 | 0.85 | 0.73-1 | 0.055 | 0.85 | 0.73-1 | 0.053 |
| Smoker status | Non-smoker |  |  |  | 0.80 | 0.66-0.99 | 0.036 | 0.80 | 0.65-0.98 | 0.034 | 0.81 | 0.66-0.99 | 0.037 |
| COPD status | Pre-existing COPD (at -24 months or earlier) |  |  |  | 1.92 | 1.66-2.22 | 0.000 | 1.92 | 1.66-2.23 | 0.000 | 1.92 | 1.66-2.22 | 0.000 |
|  | No COPD |  |  |  | 0.44 | 0.38-0.51 | 0.000 | 0.44 | 0.38-0.5 | 0.000 | 0.44 | 0.38-0.51 | 0.000 |
| Elixhauser comorbidity score | 1 |  |  |  | 1.24 | 1.04-1.46 | 0.014 | 1.23 | 1.04-1.46 | 0.015 | 1.22 | 1.03-1.45 | 0.021 |
|  | 2 |  |  |  | 1.17 | 0.98-1.39 | 0.076 | 1.17 | 0.98-1.38 | 0.077 | 1.15 | 0.97-1.37 | 0.101 |
|  | 3+ |  |  |  | 1.49 | 1.29-1.73 | 0.000 | 1.49 | 1.29-1.73 | 0.000 | 1.48 | 1.28-1.72 | 0.000 |
| Morphology | NSCLC |  |  |  |  |  |  | 1.03 | 0.87-1.22 | 0.701 | 1.00 | 0.85-1.19 | 0.962 |
|  | Unspecified |  |  |  |  |  |  | 1.01 | 0.84-1.22 | 0.919 | 0.98 | 0.81-1.18 | 0.838 |
| Stage at diagnosis | Unknown |  |  |  |  |  |  |  |  |  | 1.07 | 0.95-1.19 | 0.258 |
|  | Not advanced |  |  |  |  |  |  |  |  |  | 1.19 | 1.04-1.36 | 0.014 |

**3 selected main respiratory symptoms (-24 to -6)**

| **Variable** |  | **IRR** | **95% CI** | **p-value** | **IRR** | **95% CI** | **p-value** | **IRR** | **95% CI** | **p-value** | **IRR** | **95% CI** | **p-value** |
| --- | --- | --- | --- | --- | --- | --- | --- | --- | --- | --- | --- | --- | --- |
| Diagnostic route | GP referral | 1.39 | 1.2-1.61 | 0.000 | 1.60 | 1.39-1.85 | 0.000 | 1.60 | 1.38-1.84 | 0.000 | 1.57 | 1.36-1.81 | 0.000 |
|  | TWW | 1.00 | 0.87-1.16 | 0.950 | 1.27 | 1.1-1.47 | 0.001 | 1.27 | 1.09-1.47 | 0.002 | 1.26 | 1.08-1.46 | 0.002 |
|  | Other | 1.16 | 0.97-1.38 | 0.098 | 1.32 | 1.12-1.55 | 0.001 | 1.31 | 1.12-1.54 | 0.001 | 1.28 | 1.09-1.51 | 0.003 |
| Age at diagnosis | 85-99 |  |  |  | 0.87 | 0.73-1.02 | 0.093 | 0.87 | 0.73-1.03 | 0.103 | 0.87 | 0.73-1.03 | 0.095 |
|  | 30-64 |  |  |  | 0.74 | 0.64-0.86 | 0.000 | 0.74 | 0.64-0.86 | 0.000 | 0.74 | 0.64-0.87 | 0.000 |
|  | 65-74 |  |  |  | 0.84 | 0.74-0.95 | 0.006 | 0.84 | 0.74-0.95 | 0.006 | 0.84 | 0.74-0.95 | 0.007 |
| Gender | Female |  |  |  | 1.16 | 1.04-1.28 | 0.006 | 1.16 | 1.04-1.28 | 0.005 | 1.16 | 1.04-1.28 | 0.006 |
| Ethnicity | Non-White |  |  |  | 0.83 | 0.63-1.1 | 0.194 | 0.83 | 0.63-1.1 | 0.198 | 0.84 | 0.64-1.11 | 0.219 |
|  | Unknown |  |  |  | 0.77 | 0.5-1.19 | 0.242 | 0.77 | 0.5-1.2 | 0.252 | 0.77 | 0.5-1.19 | 0.244 |
| IMD | 2 |  |  |  | 0.90 | 0.76-1.07 | 0.215 | 0.90 | 0.76-1.07 | 0.218 | 0.90 | 0.75-1.06 | 0.211 |
|  | 3 |  |  |  | 0.90 | 0.76-1.06 | 0.217 | 0.90 | 0.76-1.07 | 0.221 | 0.90 | 0.76-1.07 | 0.220 |
|  | 4 |  |  |  | 0.90 | 0.76-1.06 | 0.215 | 0.90 | 0.76-1.06 | 0.220 | 0.90 | 0.76-1.07 | 0.227 |
|  | 5 - Most deprived |  |  |  | 0.80 | 0.68-0.95 | 0.012 | 0.81 | 0.68-0.96 | 0.013 | 0.80 | 0.68-0.95 | 0.012 |
| Smoker status | Non-smoker |  |  |  | 0.76 | 0.6-0.94 | 0.014 | 0.75 | 0.6-0.94 | 0.013 | 0.76 | 0.6-0.94 | 0.014 |
| COPD status | Pre-existing COPD (at -24 months or earlier) |  |  |  | 2.00 | 1.72-2.33 | 0.000 | 2.00 | 1.72-2.33 | 0.000 | 2.00 | 1.72-2.33 | 0.000 |
|  | No COPD |  |  |  | 0.39 | 0.34-0.46 | 0.000 | 0.39 | 0.34-0.46 | 0.000 | 0.39 | 0.34-0.46 | 0.000 |
| Elixhauser comorbidity score | 1 |  |  |  | 1.20 | 1-1.43 | 0.053 | 1.19 | 1-1.43 | 0.055 | 1.18 | 0.98-1.41 | 0.074 |
|  | 2 |  |  |  | 1.09 | 0.91-1.31 | 0.350 | 1.09 | 0.91-1.31 | 0.356 | 1.08 | 0.9-1.3 | 0.410 |
|  | 3+ |  |  |  | 1.47 | 1.26-1.71 | 0.000 | 1.47 | 1.26-1.71 | 0.000 | 1.46 | 1.25-1.71 | 0.000 |
| Morphology | NSCLC |  |  |  |  |  |  | 1.05 | 0.87-1.25 | 0.630 | 1.02 | 0.85-1.22 | 0.847 |
|  | Unspecified |  |  |  |  |  |  | 1.03 | 0.84-1.25 | 0.795 | 0.99 | 0.81-1.21 | 0.951 |
| Stage at diagnosis | Unknown |  |  |  |  |  |  |  |  |  | 1.10 | 0.98-1.24 | 0.113 |
|  | Not advanced |  |  |  |  |  |  |  |  |  | 1.18 | 1.02-1.36 | 0.030 |

# Cough consultations (-24 to -6)

| **Variable** |  | **IRR** | **95% CI** | **p-value** | **IRR** | **95% CI** | **p-value** | **IRR** | **95% CI** | **p-value** | **IRR** | **95% CI** | **p-value** |
| --- | --- | --- | --- | --- | --- | --- | --- | --- | --- | --- | --- | --- | --- |
| Diagnostic route | GP referral | 1.58 | 1.29-1.94 | 0.000 | 1.74 | 1.42-2.14 | 0.000 | 1.69 | 1.37-2.08 | 0.000 | 1.66 | 1.34-2.05 | 0.000 |
|  | TWW | 1.29 | 1.06-1.57 | 0.011 | 1.51 | 1.23-1.86 | 0.000 | 1.45 | 1.17-1.79 | 0.001 | 1.44 | 1.17-1.79 | 0.001 |
|  | Other | 1.22 | 0.96-1.55 | 0.102 | 1.34 | 1.06-1.7 | 0.016 | 1.30 | 1.02-1.65 | 0.031 | 1.27 | 1-1.62 | 0.052 |
| Age at diagnosis | 85-99 |  |  |  | 0.87 | 0.68-1.11 | 0.268 | 0.90 | 0.7-1.16 | 0.414 | 0.89 | 0.7-1.15 | 0.381 |
|  | 30-64 |  |  |  | 0.88 | 0.71-1.09 | 0.255 | 0.86 | 0.69-1.07 | 0.166 | 0.86 | 0.7-1.07 | 0.182 |
|  | 65-74 |  |  |  | 0.88 | 0.73-1.05 | 0.158 | 0.86 | 0.71-1.03 | 0.109 | 0.86 | 0.72-1.04 | 0.115 |
| Gender | Female |  |  |  | 1.36 | 1.17-1.58 | 0.000 | 1.37 | 1.18-1.59 | 0.000 | 1.37 | 1.18-1.59 | 0.000 |
| Ethnicity | Non-White |  |  |  | 0.84 | 0.57-1.23 | 0.377 | 0.85 | 0.58-1.26 | 0.424 | 0.86 | 0.59-1.27 | 0.454 |
|  | Unknown |  |  |  | 0.70 | 0.37-1.3 | 0.256 | 0.74 | 0.39-1.38 | 0.343 | 0.73 | 0.39-1.37 | 0.324 |
| IMD | 2 |  |  |  | 0.91 | 0.71-1.17 | 0.459 | 0.92 | 0.72-1.18 | 0.501 | 0.92 | 0.72-1.18 | 0.499 |
|  | 3 |  |  |  | 0.92 | 0.73-1.18 | 0.528 | 0.93 | 0.73-1.18 | 0.556 | 0.93 | 0.73-1.18 | 0.547 |
|  | 4 |  |  |  | 0.94 | 0.74-1.19 | 0.606 | 0.95 | 0.75-1.21 | 0.660 | 0.95 | 0.75-1.21 | 0.693 |
|  | 5 - Most deprived |  |  |  | 0.79 | 0.62-1.01 | 0.063 | 0.80 | 0.63-1.03 | 0.082 | 0.80 | 0.62-1.02 | 0.076 |
| Smoker status | Non-smoker |  |  |  | 0.76 | 0.56-1.02 | 0.070 | 0.75 | 0.56-1.01 | 0.062 | 0.76 | 0.56-1.02 | 0.065 |
| COPD status | Pre-existing COPD (at -24 months or earlier) |  |  |  | 1.39 | 1.1-1.75 | 0.005 | 1.41 | 1.12-1.78 | 0.004 | 1.41 | 1.12-1.78 | 0.004 |
|  | No COPD |  |  |  | 0.54 | 0.44-0.68 | 0.000 | 0.54 | 0.44-0.67 | 0.000 | 0.55 | 0.44-0.68 | 0.000 |
| Elixhauser comorbidity score | 1 |  |  |  | 1.25 | 0.97-1.6 | 0.079 | 1.25 | 0.97-1.6 | 0.083 | 1.23 | 0.96-1.58 | 0.106 |
|  | 2 |  |  |  | 1.15 | 0.89-1.48 | 0.280 | 1.15 | 0.89-1.48 | 0.288 | 1.14 | 0.88-1.47 | 0.316 |
|  | 3+ |  |  |  | 1.40 | 1.13-1.75 | 0.003 | 1.40 | 1.12-1.74 | 0.003 | 1.40 | 1.13-1.75 | 0.003 |
| Morphology | NSCLC |  |  |  |  |  |  | 1.08 | 0.84-1.39 | 0.555 | 1.05 | 0.81-1.36 | 0.701 |
|  | Unspecified |  |  |  |  |  |  | 0.91 | 0.68-1.22 | 0.535 | 0.87 | 0.65-1.16 | 0.346 |
| Stage at diagnosis | Unknown |  |  |  |  |  |  |  |  |  | 1.23 | 1.04-1.46 | 0.014 |
|  | Not advanced |  |  |  |  |  |  |  |  |  | 1.21 | 0.98-1.5 | 0.083 |

**Dyspnoea consultations (-24 to -6)**

| **Variable** |  | **IRR** | **95% CI** | **p-value** | **IRR** | **95% CI** | **p-value** | **IRR** | **95% CI** | **p-value** | **IRR** | **95% CI** | **p-value** |
| --- | --- | --- | --- | --- | --- | --- | --- | --- | --- | --- | --- | --- | --- |
| Diagnostic route | GP referral | 1.15 | 0.93-1.41 | 0.195 | 1.36 | 1.14-1.62 | 0.001 | 1.39 | 1.16-1.67 | 0.000 | 1.37 | 1.14-1.64 | 0.001 |
|  | TWW | 0.79 | 0.64-0.97 | 0.022 | 1.03 | 0.86-1.24 | 0.746 | 1.06 | 0.88-1.28 | 0.543 | 1.05 | 0.87-1.26 | 0.645 |
|  | Other | 1.05 | 0.83-1.33 | 0.667 | 1.20 | 0.98-1.46 | 0.081 | 1.22 | 0.99-1.49 | 0.058 | 1.20 | 0.97-1.47 | 0.087 |
| Age at diagnosis | 85-99 |  |  |  | 0.87 | 0.7-1.07 | 0.182 | 0.85 | 0.68-1.05 | 0.131 | 0.85 | 0.68-1.05 | 0.134 |
|  | 30-64 |  |  |  | 0.59 | 0.48-0.73 | 0.000 | 0.60 | 0.48-0.74 | 0.000 | 0.60 | 0.49-0.75 | 0.000 |
|  | 65-74 |  |  |  | 0.83 | 0.71-0.97 | 0.021 | 0.85 | 0.72-0.99 | 0.035 | 0.85 | 0.73-0.99 | 0.039 |
| Gender | Female |  |  |  | 0.93 | 0.81-1.06 | 0.263 | 0.92 | 0.81-1.05 | 0.239 | 0.92 | 0.81-1.05 | 0.224 |
| Ethnicity | Non-White |  |  |  | 0.78 | 0.52-1.15 | 0.211 | 0.77 | 0.52-1.14 | 0.188 | 0.78 | 0.52-1.15 | 0.207 |
|  | Unknown |  |  |  | 0.94 | 0.53-1.68 | 0.844 | 0.91 | 0.51-1.63 | 0.759 | 0.91 | 0.51-1.63 | 0.754 |
| IMD | 2 |  |  |  | 0.86 | 0.69-1.07 | 0.176 | 0.85 | 0.68-1.07 | 0.162 | 0.85 | 0.68-1.06 | 0.151 |
|  | 3 |  |  |  | 0.89 | 0.72-1.11 | 0.312 | 0.89 | 0.72-1.11 | 0.301 | 0.89 | 0.72-1.11 | 0.301 |
|  | 4 |  |  |  | 0.83 | 0.67-1.03 | 0.093 | 0.82 | 0.66-1.03 | 0.083 | 0.82 | 0.66-1.02 | 0.082 |
|  | 5 - Most deprived |  |  |  | 0.81 | 0.65-1.01 | 0.058 | 0.80 | 0.65-1 | 0.048 | 0.80 | 0.65-1 | 0.047 |
| Smoker status | Non-smoker |  |  |  | 0.65 | 0.44-0.95 | 0.025 | 0.65 | 0.45-0.95 | 0.026 | 0.65 | 0.45-0.95 | 0.027 |
| COPD status | Pre-existing COPD (at -24 months or earlier) |  |  |  | 2.48 | 2.08-2.96 | 0.000 | 2.46 | 2.06-2.94 | 0.000 | 2.44 | 2.04-2.92 | 0.000 |
|  | No COPD |  |  |  | 0.22 | 0.18-0.27 | 0.000 | 0.22 | 0.18-0.27 | 0.000 | 0.22 | 0.18-0.27 | 0.000 |
| Elixhauser comorbidity score | 1 |  |  |  | 1.16 | 0.89-1.5 | 0.271 | 1.16 | 0.89-1.5 | 0.276 | 1.15 | 0.88-1.49 | 0.305 |
|  | 2 |  |  |  | 1.09 | 0.84-1.41 | 0.511 | 1.09 | 0.84-1.41 | 0.510 | 1.08 | 0.83-1.4 | 0.567 |
|  | 3+ |  |  |  | 1.55 | 1.25-1.92 | 0.000 | 1.56 | 1.26-1.93 | 0.000 | 1.55 | 1.25-1.92 | 0.000 |
| Morphology | NSCLC |  |  |  |  |  |  | 0.95 | 0.75-1.2 | 0.679 | 0.93 | 0.74-1.18 | 0.563 |
|  | Unspecified |  |  |  |  |  |  | 1.06 | 0.83-1.36 | 0.647 | 1.05 | 0.81-1.35 | 0.715 |
| Stage at diagnosis | Unknown |  |  |  |  |  |  |  |  |  | 0.98 | 0.84-1.14 | 0.780 |
|  | Not advanced |  |  |  |  |  |  |  |  |  | 1.13 | 0.94-1.35 | 0.202 |

# Haemoptysis consultations (-24 to -6)

| **Variable** |  | **IRR** | **95% CI** | **p-value** | **IRR** | **95% CI** | **p-value** | **IRR** | **95% CI** | **p-value** | **IRR** | **95% CI** | **p-value** |
| --- | --- | --- | --- | --- | --- | --- | --- | --- | --- | --- | --- | --- | --- |
| Diagnostic route | GP referral | 2.71 | 0.47-15.76 | 0.267 | 3.49 | 0.02-572.28 | 0.631 | 4.04 | 0.67-24.41 | 0.128 | 4.12 | 0.67-25.38 | 0.127 |
|  | TWW | 0.50 | 0.04-6.75 | 0.601 | 0.70 | 0-1258.33 | 0.927 | 0.86 | 0.07-11.17 | 0.907 | 0.88 | 0.07-11.52 | 0.922 |
|  | Other | 1.86 | 0.23-14.7 | 0.557 | 2.43 | 0.01-988.43 | 0.772 | 2.74 | 0.34-21.93 | 0.342 | 2.78 | 0.34-22.76 | 0.341 |
| Age at diagnosis | 85-99 |  |  |  | 1.43 | 0-554.45 | 0.906 | 1.24 | 0.17-9.04 | 0.835 | 1.23 | 0.17-9.05 | 0.836 |
|  | 30-64 |  |  |  | 1.04 | 0.01-187.32 | 0.990 | 1.20 | 0.18-8.18 | 0.852 | 1.21 | 0.18-8.31 | 0.844 |
|  | 65-74 |  |  |  | 0.63 | 0-121.98 | 0.864 | 0.69 | 0.11-4.21 | 0.688 | 0.69 | 0.11-4.21 | 0.687 |
| Gender | Female |  |  |  | 0.94 | 0.02-53.82 | 0.976 | 0.92 | 0.23-3.63 | 0.906 | 0.93 | 0.23-3.69 | 0.919 |
| Ethnicity | Non-White |  |  |  | 0.45 | 0-1745703.31 | 0.918 | 0.42 | 0-53.1 | 0.723 | 0.41 | 0-53.35 | 0.718 |
|  | Unknown |  |  |  | 0.00 | 0-Inf | 1.000 | 0.00 | 0-5.92055872024073e+131 | 0.931 | 0.00 | 0-4.07743582102134e+301 | 0.967 |
| IMD | 2 |  |  |  | 2.49 | 0-4383.14 | 0.811 | 2.35 | 0.2-27.81 | 0.499 | 2.36 | 0.2-28.29 | 0.497 |
|  | 3 |  |  |  | 1.44 | 0-5028.93 | 0.930 | 1.46 | 0.1-20.82 | 0.779 | 1.47 | 0.1-21.08 | 0.775 |
|  | 4 |  |  |  | 2.07 | 0-4452.27 | 0.852 | 2.06 | 0.17-25.32 | 0.572 | 2.07 | 0.17-25.64 | 0.571 |
|  | 5 - Most deprived |  |  |  | 1.35 | 0-4497.72 | 0.942 | 1.30 | 0.09-19.14 | 0.848 | 1.31 | 0.09-19.39 | 0.845 |
| Smoker status | Non-smoker |  |  |  | 1.45 | 0-2591.32 | 0.923 | 1.49 | 0.14-16.29 | 0.742 | 1.48 | 0.14-16.14 | 0.748 |
| COPD status | Pre-existing COPD (at -24 months or earlier) |  |  |  | 0.82 | 0-141.66 | 0.941 | 0.77 | 0.12-4.85 | 0.777 | 0.77 | 0.12-4.93 | 0.785 |
|  | No COPD |  |  |  | 0.36 | 0-130.57 | 0.736 | 0.36 | 0.06-2.24 | 0.275 | 0.36 | 0.06-2.25 | 0.275 |
| Elixhauser comorbidity score | 1 |  |  |  | 1.09 | 0-4362.7 | 0.985 | 1.10 | 0.07-16.22 | 0.944 | 1.11 | 0.07-16.56 | 0.938 |
|  | 2 |  |  |  | 1.21 | 0-3267.34 | 0.962 | 1.24 | 0.1-16.08 | 0.870 | 1.25 | 0.1-16.31 | 0.864 |
|  | 3+ |  |  |  | 1.78 | 0-1451.06 | 0.866 | 1.80 | 0.21-15.59 | 0.593 | 1.82 | 0.21-15.92 | 0.586 |
| Morphology | NSCLC |  |  |  |  |  |  | 0.95 | 0.06-14.32 | 0.973 | 0.97 | 0.06-14.94 | 0.983 |
|  | Unspecified |  |  |  |  |  |  | 1.86 | 0.11-31.63 | 0.668 | 1.83 | 0.1-32.41 | 0.680 |
| Stage at diagnosis | Unknown |  |  |  |  |  |  |  |  |  | 1.18 | 0.26-5.39 | 0.828 |
|  | Not advanced |  |  |  |  |  |  |  |  |  | 0.91 | 0.12-6.65 | 0.927 |

# Any chest imaging event (-24 to -6)

| **Variable** |  | **IRR** | **95% CI** | **p-value** | **IRR** | **95% CI** | **p-value** | **IRR** | **95% CI** | **p-value** | **IRR** | **95% CI** | **p-value** |
| --- | --- | --- | --- | --- | --- | --- | --- | --- | --- | --- | --- | --- | --- |
| Diagnostic route | GP referral | 1.39 | 0.91-2.12 | 0.126 | 2.11 | 1.41-3.16 | 0.000 | 2.12 | 1.41-3.19 | 0.000 | 1.80 | 1.24-2.62 | 0.002 |
|  | TWW | 0.65 | 0.41-1.02 | 0.059 | 1.04 | 0.68-1.6 | 0.847 | 1.06 | 0.68-1.64 | 0.806 | 0.81 | 0.54-1.22 | 0.315 |
|  | Other | 1.09 | 0.66-1.79 | 0.734 | 1.52 | 0.96-2.42 | 0.075 | 1.53 | 0.96-2.45 | 0.073 | 1.29 | 0.84-1.99 | 0.250 |
| Age at diagnosis | 85-99 |  |  |  | 1.33 | 0.84-2.1 | 0.217 | 1.31 | 0.83-2.09 | 0.248 | 1.35 | 0.89-2.06 | 0.159 |
|  | 30-64 |  |  |  | 0.66 | 0.42-1.04 | 0.074 | 0.67 | 0.43-1.06 | 0.087 | 0.65 | 0.43-0.99 | 0.045 |
|  | 65-74 |  |  |  | 0.73 | 0.5-1.07 | 0.104 | 0.74 | 0.5-1.07 | 0.112 | 0.74 | 0.52-1.04 | 0.086 |
| Gender | Female |  |  |  | 1.28 | 0.95-1.74 | 0.109 | 1.28 | 0.95-1.74 | 0.109 | 1.24 | 0.94-1.64 | 0.121 |
| Ethnicity | Non-White |  |  |  | 0.52 | 0.21-1.26 | 0.146 | 0.51 | 0.21-1.25 | 0.142 | 0.53 | 0.24-1.21 | 0.132 |
|  | Unknown |  |  |  | 1.54 | 0.52-4.52 | 0.432 | 1.51 | 0.51-4.46 | 0.458 | 1.67 | 0.62-4.5 | 0.312 |
| IMD | 2 |  |  |  | 0.93 | 0.56-1.55 | 0.793 | 0.93 | 0.56-1.54 | 0.783 | 0.92 | 0.58-1.46 | 0.719 |
|  | 3 |  |  |  | 0.86 | 0.52-1.43 | 0.565 | 0.86 | 0.52-1.43 | 0.567 | 0.87 | 0.55-1.38 | 0.565 |
|  | 4 |  |  |  | 1.20 | 0.74-1.94 | 0.471 | 1.20 | 0.74-1.94 | 0.472 | 1.23 | 0.79-1.9 | 0.367 |
|  | 5 - Most deprived |  |  |  | 0.99 | 0.6-1.63 | 0.960 | 0.99 | 0.6-1.63 | 0.959 | 1.01 | 0.64-1.59 | 0.977 |
| Smoker status | Non-smoker |  |  |  | 0.92 | 0.52-1.62 | 0.760 | 0.91 | 0.51-1.61 | 0.743 | 0.98 | 0.58-1.63 | 0.924 |
| COPD status | Pre-existing COPD (at -24 months or earlier) |  |  |  | 1.10 | 0.69-1.75 | 0.686 | 1.10 | 0.69-1.74 | 0.699 | 0.98 | 0.64-1.5 | 0.932 |
|  | No COPD |  |  |  | 0.52 | 0.34-0.81 | 0.003 | 0.52 | 0.34-0.81 | 0.003 | 0.51 | 0.34-0.75 | 0.001 |
| Elixhauser comorbidity score | 1 |  |  |  | 1.40 | 0.79-2.49 | 0.250 | 1.40 | 0.79-2.48 | 0.254 | 1.28 | 0.76-2.17 | 0.353 |
|  | 2 |  |  |  | 1.54 | 0.87-2.72 | 0.136 | 1.54 | 0.87-2.71 | 0.136 | 1.40 | 0.84-2.36 | 0.198 |
|  | 3+ |  |  |  | 3.43 | 2.12-5.56 | 0.000 | 3.44 | 2.12-5.56 | 0.000 | 3.32 | 2.15-5.14 | 0.000 |
| Morphology | NSCLC |  |  |  |  |  |  | 1.12 | 0.65-1.93 | 0.688 | 0.93 | 0.56-1.54 | 0.780 |
|  | Unspecified |  |  |  |  |  |  | 1.16 | 0.64-2.1 | 0.625 | 1.23 | 0.71-2.12 | 0.456 |
| Stage at diagnosis | Unknown |  |  |  |  |  |  |  |  |  | 0.33 | 0.23-0.47 | 0.000 |
|  | Not advanced |  |  |  |  |  |  |  |  |  | 2.47 | 1.73-3.52 | 0.000 |

**Chest X-rays (-24 to -6)**

| **Variable** |  | **IRR** | **95% CI** | **p-value** | **IRR** | **95% CI** | **p-value** | **IRR** | **95% CI** | **p-value** | **IRR** | **95% CI** | **p-value** |
| --- | --- | --- | --- | --- | --- | --- | --- | --- | --- | --- | --- | --- | --- |
| Diagnostic route | GP referral | 1.29 | 0.85-1.97 | 0.235 | 1.91 | 1.3-2.81 | 0.001 | 1.94 | 1.31-2.87 | 0.001 | 1.64 | 1.14-2.37 | 0.008 |
|  | TWW | 0.68 | 0.44-1.06 | 0.087 | 1.09 | 0.72-1.65 | 0.674 | 1.12 | 0.73-1.7 | 0.611 | 0.88 | 0.59-1.3 | 0.525 |
|  | Other | 0.99 | 0.6-1.63 | 0.971 | 1.36 | 0.86-2.12 | 0.185 | 1.37 | 0.87-2.16 | 0.170 | 1.18 | 0.77-1.8 | 0.452 |
| Age at diagnosis | 85-99 |  |  |  | 1.38 | 0.9-2.14 | 0.143 | 1.36 | 0.87-2.11 | 0.175 | 1.39 | 0.92-2.09 | 0.114 |
|  | 30-64 |  |  |  | 0.69 | 0.45-1.06 | 0.087 | 0.70 | 0.45-1.08 | 0.105 | 0.68 | 0.46-1.03 | 0.066 |
|  | 65-74 |  |  |  | 0.72 | 0.5-1.04 | 0.078 | 0.73 | 0.51-1.05 | 0.088 | 0.73 | 0.52-1.03 | 0.069 |
| Gender | Female |  |  |  | 1.27 | 0.95-1.7 | 0.113 | 1.26 | 0.94-1.69 | 0.116 | 1.22 | 0.93-1.6 | 0.146 |
| Ethnicity | Non-White |  |  |  | 0.53 | 0.22-1.25 | 0.145 | 0.52 | 0.22-1.23 | 0.139 | 0.54 | 0.24-1.21 | 0.136 |
|  | Unknown |  |  |  | 1.68 | 0.61-4.66 | 0.316 | 1.63 | 0.58-4.55 | 0.350 | 1.82 | 0.7-4.71 | 0.218 |
| IMD | 2 |  |  |  | 0.94 | 0.58-1.53 | 0.805 | 0.94 | 0.58-1.52 | 0.790 | 0.92 | 0.59-1.45 | 0.734 |
|  | 3 |  |  |  | 0.88 | 0.54-1.43 | 0.602 | 0.88 | 0.54-1.43 | 0.600 | 0.89 | 0.57-1.4 | 0.625 |
|  | 4 |  |  |  | 1.15 | 0.72-1.84 | 0.562 | 1.15 | 0.72-1.83 | 0.570 | 1.17 | 0.76-1.8 | 0.490 |
|  | 5 - Most deprived |  |  |  | 1.03 | 0.64-1.67 | 0.899 | 1.03 | 0.64-1.66 | 0.914 | 1.05 | 0.67-1.64 | 0.832 |
| Smoker status | Non-smoker |  |  |  | 0.85 | 0.48-1.48 | 0.556 | 0.84 | 0.48-1.48 | 0.551 | 0.91 | 0.54-1.52 | 0.710 |
| COPD status | Pre-existing COPD (at -24 months or earlier) |  |  |  | 1.06 | 0.68-1.64 | 0.801 | 1.05 | 0.68-1.64 | 0.822 | 0.95 | 0.63-1.43 | 0.808 |
|  | No COPD |  |  |  | 0.50 | 0.33-0.76 | 0.001 | 0.50 | 0.33-0.76 | 0.001 | 0.49 | 0.33-0.71 | 0.000 |
| Elixhauser comorbidity score | 1 |  |  |  | 1.32 | 0.76-2.3 | 0.326 | 1.32 | 0.76-2.3 | 0.327 | 1.21 | 0.72-2.04 | 0.465 |
|  | 2 |  |  |  | 1.49 | 0.86-2.58 | 0.151 | 1.50 | 0.87-2.58 | 0.149 | 1.37 | 0.82-2.28 | 0.225 |
|  | 3+ |  |  |  | 3.29 | 2.07-5.23 | 0.000 | 3.30 | 2.08-5.23 | 0.000 | 3.15 | 2.06-4.84 | 0.000 |
| Morphology | NSCLC |  |  |  |  |  |  | 1.04 | 0.62-1.74 | 0.884 | 0.89 | 0.55-1.44 | 0.629 |
|  | Unspecified |  |  |  |  |  |  | 1.12 | 0.64-1.97 | 0.694 | 1.21 | 0.71-2.05 | 0.485 |
| Stage at diagnosis | Unknown |  |  |  |  |  |  |  |  |  | 0.33 | 0.23-0.47 | 0.000 |
|  | Not advanced |  |  |  |  |  |  |  |  |  | 2.09 | 1.48-2.96 | 0.000 |

# Chest CT scans (-24 to -6)

| **Variable** |  | **IRR** | **95% CI** | **p-value** | **IRR** | **95% CI** | **p-value** | **IRR** | **95% CI** | **p-value** | **IRR** | **95% CI** | **p-value** |
| --- | --- | --- | --- | --- | --- | --- | --- | --- | --- | --- | --- | --- | --- |
| Diagnostic route | GP referral | 2.19 | 0.81-5.87 | 0.121 | 2.91 | 1.07-7.9 | 0.036 | 2.78 | 1.01-7.69 | 0.049 | 2.15 | 0.79-5.83 | 0.132 |
|  | TWW | 0.31 | 0.06-1.57 | 0.157 | 0.46 | 0.09-2.28 | 0.342 | 0.45 | 0.09-2.24 | 0.327 | 0.32 | 0.07-1.53 | 0.154 |
|  | Other | 1.81 | 0.58-5.65 | 0.306 | 2.33 | 0.75-7.21 | 0.142 | 2.25 | 0.72-7.07 | 0.163 | 1.69 | 0.55-5.18 | 0.358 |
| Age at diagnosis | 85-99 |  |  |  | 1.02 | 0.3-3.45 | 0.973 | 1.04 | 0.3-3.56 | 0.955 | 1.03 | 0.32-3.39 | 0.957 |
|  | 30-64 |  |  |  | 0.57 | 0.15-2.09 | 0.395 | 0.56 | 0.15-2.06 | 0.379 | 0.52 | 0.15-1.87 | 0.319 |
|  | 65-74 |  |  |  | 0.87 | 0.34-2.24 | 0.778 | 0.86 | 0.33-2.21 | 0.752 | 0.89 | 0.35-2.23 | 0.796 |
| Gender | Female |  |  |  | 1.44 | 0.64-3.2 | 0.376 | 1.46 | 0.65-3.25 | 0.358 | 1.39 | 0.64-3.02 | 0.400 |
| Ethnicity | Non-White |  |  |  | 0.20 | 0-12.73 | 0.447 | 0.20 | 0-12.45 | 0.444 | 0.24 | 0.01-11.18 | 0.465 |
|  | Unknown |  |  |  | 0.83 | 0.03-24.89 | 0.915 | 0.88 | 0.03-26.39 | 0.939 | 0.86 | 0.03-23.28 | 0.929 |
| IMD | 2 |  |  |  | 1.15 | 0.3-4.36 | 0.836 | 1.16 | 0.31-4.4 | 0.827 | 1.11 | 0.31-4.02 | 0.875 |
|  | 3 |  |  |  | 0.88 | 0.22-3.52 | 0.851 | 0.89 | 0.22-3.56 | 0.866 | 0.87 | 0.23-3.29 | 0.832 |
|  | 4 |  |  |  | 1.55 | 0.44-5.48 | 0.493 | 1.58 | 0.45-5.55 | 0.479 | 1.65 | 0.49-5.6 | 0.419 |
|  | 5 - Most deprived |  |  |  | 0.96 | 0.24-3.77 | 0.953 | 0.99 | 0.25-3.87 | 0.983 | 0.96 | 0.26-3.6 | 0.953 |
| Smoker status | Non-smoker |  |  |  | 1.20 | 0.29-5.06 | 0.799 | 1.17 | 0.28-4.89 | 0.834 | 1.26 | 0.32-5.03 | 0.744 |
| COPD status | Pre-existing COPD (at -24 months or earlier) |  |  |  | 0.81 | 0.26-2.48 | 0.712 | 0.82 | 0.27-2.5 | 0.725 | 0.75 | 0.25-2.23 | 0.605 |
|  | No COPD |  |  |  | 0.49 | 0.17-1.43 | 0.190 | 0.49 | 0.17-1.42 | 0.188 | 0.51 | 0.18-1.44 | 0.205 |
| Elixhauser comorbidity score | 1 |  |  |  | 2.34 | 0.38-14.5 | 0.362 | 2.26 | 0.37-13.86 | 0.379 | 1.97 | 0.35-11.17 | 0.446 |
|  | 2 |  |  |  | 2.10 | 0.34-13.13 | 0.426 | 2.05 | 0.33-12.67 | 0.441 | 1.69 | 0.29-9.76 | 0.557 |
|  | 3+ |  |  |  | 3.89 | 0.78-19.29 | 0.096 | 3.84 | 0.78-18.86 | 0.098 | 3.47 | 0.76-15.88 | 0.109 |
| Morphology | NSCLC |  |  |  |  |  |  | 2.02 | 0.33-12.34 | 0.448 | 1.43 | 0.25-8.27 | 0.690 |
|  | Unspecified |  |  |  |  |  |  | 1.68 | 0.24-11.5 | 0.599 | 1.37 | 0.21-8.73 | 0.740 |
| Stage at diagnosis | Unknown |  |  |  |  |  |  |  |  |  | 0.71 | 0.25-2.02 | 0.523 |
|  | Not advanced |  |  |  |  |  |  |  |  |  | 4.11 | 1.68-10.08 | 0.002 |

**Supplementary Table S14.** Modelled incidence rate ratios (IRRs) for pre-diagnostic healthcare use, by diagnostic route, with and without adjustment for socio-demographic and tumour factors (-12 to -6 months). (NOTE: data on patients with haemoptysis was insufficient for analysis in this segmentation.)

# Primary care consultations (-12 to -6)

| **Variable** |  | **IRR** | **95% CI** | **p-value** | **IRR** | **95% CI** | **p-value** | **IRR** | **95% CI** | **p-value** | **IRR** | **95% CI** | **p-value** |
| --- | --- | --- | --- | --- | --- | --- | --- | --- | --- | --- | --- | --- | --- |
| Diagnostic route | GP referral | 1.13 | 1.05-1.22 | 0.002 | 1.34 | 1.24-1.44 | 0.000 | 1.34 | 1.24-1.44 | 0.000 | 1.32 | 1.22-1.42 | 0.000 |
|  | TWW | 0.81 | 0.76-0.88 | 0.000 | 1.06 | 0.98-1.14 | 0.148 | 1.05 | 0.98-1.14 | 0.167 | 1.05 | 0.98-1.14 | 0.177 |
|  | Other | 0.94 | 0.86-1.03 | 0.188 | 1.11 | 1.02-1.21 | 0.012 | 1.11 | 1.02-1.21 | 0.013 | 1.10 | 1.01-1.19 | 0.037 |
| Age at diagnosis | 85-99 |  |  |  | 1.07 | 0.99-1.17 | 0.103 | 1.08 | 0.99-1.17 | 0.102 | 1.07 | 0.98-1.17 | 0.120 |
|  | 30-64 |  |  |  | 0.72 | 0.67-0.78 | 0.000 | 0.72 | 0.66-0.78 | 0.000 | 0.72 | 0.67-0.78 | 0.000 |
|  | 65-74 |  |  |  | 0.86 | 0.81-0.92 | 0.000 | 0.86 | 0.81-0.92 | 0.000 | 0.87 | 0.81-0.93 | 0.000 |
| Gender | Female |  |  |  | 1.15 | 1.09-1.21 | 0.000 | 1.15 | 1.09-1.21 | 0.000 | 1.15 | 1.09-1.22 | 0.000 |
| Ethnicity | Non-White |  |  |  | 0.85 | 0.75-0.97 | 0.015 | 0.85 | 0.75-0.97 | 0.015 | 0.85 | 0.75-0.97 | 0.018 |
|  | Unknown |  |  |  | 0.79 | 0.65-0.98 | 0.028 | 0.80 | 0.65-0.98 | 0.030 | 0.79 | 0.64-0.97 | 0.025 |
| IMD | 2 |  |  |  | 0.96 | 0.88-1.05 | 0.432 | 0.96 | 0.88-1.06 | 0.435 | 0.96 | 0.88-1.05 | 0.420 |
|  | 3 |  |  |  | 1.02 | 0.94-1.11 | 0.636 | 1.02 | 0.94-1.11 | 0.638 | 1.02 | 0.93-1.11 | 0.666 |
|  | 4 |  |  |  | 0.91 | 0.84-1 | 0.043 | 0.91 | 0.84-1 | 0.044 | 0.92 | 0.84-1 | 0.047 |
|  | 5 - Most deprived |  |  |  | 0.93 | 0.85-1.02 | 0.127 | 0.93 | 0.85-1.02 | 0.128 | 0.93 | 0.85-1.01 | 0.102 |
| Smoker status | Non-smoker |  |  |  | 1.04 | 0.94-1.14 | 0.430 | 1.04 | 0.95-1.14 | 0.422 | 1.04 | 0.95-1.14 | 0.426 |
| COPD status | Pre-existing COPD (at -24 months or earlier) |  |  |  | 1.14 | 1.04-1.25 | 0.004 | 1.14 | 1.04-1.25 | 0.004 | 1.14 | 1.05-1.25 | 0.003 |
|  | No COPD |  |  |  | 0.81 | 0.75-0.88 | 0.000 | 0.81 | 0.75-0.88 | 0.000 | 0.82 | 0.75-0.89 | 0.000 |
| Elixhauser comorbidity score | 1 |  |  |  | 1.21 | 1.11-1.32 | 0.000 | 1.21 | 1.11-1.32 | 0.000 | 1.20 | 1.1-1.31 | 0.000 |
|  | 2 |  |  |  | 1.34 | 1.22-1.46 | 0.000 | 1.34 | 1.22-1.46 | 0.000 | 1.33 | 1.22-1.46 | 0.000 |
|  | 3+ |  |  |  | 1.77 | 1.64-1.92 | 0.000 | 1.77 | 1.64-1.92 | 0.000 | 1.77 | 1.64-1.92 | 0.000 |
| Morphology | NSCLC |  |  |  |  |  |  | 0.99 | 0.9-1.08 | 0.788 | 0.97 | 0.89-1.07 | 0.566 |
|  | Unspecified |  |  |  |  |  |  | 0.98 | 0.89-1.09 | 0.761 | 0.96 | 0.86-1.06 | 0.418 |
| Stage at diagnosis | Unknown |  |  |  |  |  |  |  |  |  | 1.13 | 1.06-1.2 | 0.000 |
|  | Not advanced |  |  |  |  |  |  |  |  |  | 1.13 | 1.04-1.22 | 0.004 |

# 6 selected relevant symptoms (-12 to -6)

| **Variable** |  | **IRR** | **95% CI** | **p-value** | **IRR** | **95% CI** | **p-value** | **IRR** | **95% CI** | **p-value** | **IRR** | **95% CI** | **p-value** |
| --- | --- | --- | --- | --- | --- | --- | --- | --- | --- | --- | --- | --- | --- |
| Diagnostic route | GP referral | 1.50 | 1.26-1.78 | 0.000 | 1.71 | 1.43-2.04 | 0.000 | 1.67 | 1.4-2 | 0.000 | 1.63 | 1.37-1.96 | 0.000 |
|  | TWW | 1.07 | 0.9-1.27 | 0.472 | 1.32 | 1.1-1.58 | 0.003 | 1.28 | 1.06-1.54 | 0.009 | 1.26 | 1.05-1.52 | 0.015 |
|  | Other | 1.22 | 0.99-1.49 | 0.060 | 1.39 | 1.14-1.71 | 0.001 | 1.37 | 1.12-1.68 | 0.002 | 1.33 | 1.09-1.64 | 0.006 |
| Age at diagnosis | 85-99 |  |  |  | 0.87 | 0.7-1.07 | 0.189 | 0.89 | 0.72-1.1 | 0.291 | 0.89 | 0.72-1.1 | 0.279 |
|  | 30-64 |  |  |  | 0.76 | 0.63-0.91 | 0.004 | 0.74 | 0.61-0.9 | 0.002 | 0.75 | 0.62-0.9 | 0.002 |
|  | 65-74 |  |  |  | 0.80 | 0.68-0.94 | 0.005 | 0.79 | 0.68-0.93 | 0.004 | 0.79 | 0.68-0.93 | 0.004 |
| Gender | Female |  |  |  | 1.18 | 1.03-1.34 | 0.014 | 1.18 | 1.04-1.34 | 0.012 | 1.18 | 1.03-1.34 | 0.013 |
| Ethnicity | Non-White |  |  |  | 0.91 | 0.65-1.28 | 0.595 | 0.92 | 0.66-1.29 | 0.644 | 0.94 | 0.67-1.31 | 0.696 |
|  | Unknown |  |  |  | 0.67 | 0.38-1.18 | 0.165 | 0.69 | 0.39-1.23 | 0.211 | 0.69 | 0.39-1.23 | 0.207 |
| IMD | 2 |  |  |  | 0.85 | 0.69-1.06 | 0.146 | 0.86 | 0.69-1.06 | 0.162 | 0.86 | 0.69-1.06 | 0.161 |
|  | 3 |  |  |  | 0.88 | 0.71-1.08 | 0.212 | 0.88 | 0.71-1.08 | 0.219 | 0.88 | 0.71-1.08 | 0.226 |
|  | 4 |  |  |  | 0.79 | 0.64-0.98 | 0.029 | 0.80 | 0.65-0.98 | 0.035 | 0.80 | 0.65-0.99 | 0.036 |
|  | 5 - Most deprived |  |  |  | 0.86 | 0.7-1.06 | 0.155 | 0.87 | 0.7-1.07 | 0.182 | 0.87 | 0.7-1.07 | 0.180 |
| Smoker status | Non-smoker |  |  |  | 0.85 | 0.65-1.11 | 0.227 | 0.85 | 0.65-1.1 | 0.221 | 0.85 | 0.65-1.11 | 0.234 |
| COPD status | Pre-existing COPD (at -24 months or earlier) |  |  |  | 1.74 | 1.44-2.1 | 0.000 | 1.75 | 1.45-2.12 | 0.000 | 1.75 | 1.44-2.12 | 0.000 |
|  | No COPD |  |  |  | 0.48 | 0.4-0.58 | 0.000 | 0.48 | 0.39-0.57 | 0.000 | 0.48 | 0.4-0.58 | 0.000 |
| Elixhauser comorbidity score | 1 |  |  |  | 1.15 | 0.92-1.43 | 0.225 | 1.15 | 0.92-1.43 | 0.226 | 1.13 | 0.91-1.42 | 0.270 |
|  | 2 |  |  |  | 1.06 | 0.84-1.33 | 0.621 | 1.06 | 0.84-1.33 | 0.628 | 1.05 | 0.84-1.32 | 0.685 |
|  | 3+ |  |  |  | 1.34 | 1.1-1.62 | 0.003 | 1.34 | 1.1-1.62 | 0.003 | 1.33 | 1.1-1.61 | 0.004 |
| Morphology | NSCLC |  |  |  |  |  |  | 1.01 | 0.81-1.26 | 0.920 | 0.98 | 0.78-1.23 | 0.861 |
|  | Unspecified |  |  |  |  |  |  | 0.91 | 0.71-1.16 | 0.439 | 0.88 | 0.68-1.13 | 0.309 |
| Stage at diagnosis | Unknown |  |  |  |  |  |  |  |  |  | 1.07 | 0.92-1.24 | 0.369 |
|  | Not advanced |  |  |  |  |  |  |  |  |  | 1.20 | 1.01-1.44 | 0.044 |

# 3 selected main respiratory symptoms (-12 to -6)

| **Variable** |  | **IRR** | **95% CI** | **p-value** | **IRR** | **95% CI** | **p-value** | **IRR** | **95% CI** | **p-value** | **IRR** | **95% CI** | **p-value** |
| --- | --- | --- | --- | --- | --- | --- | --- | --- | --- | --- | --- | --- | --- |
| Diagnostic route | GP referral | 1.50 | 1.24-1.8 | 0.000 | 1.72 | 1.42-2.07 | 0.000 | 1.68 | 1.39-2.03 | 0.000 | 1.65 | 1.36-1.99 | 0.000 |
|  | TWW | 1.09 | 0.91-1.31 | 0.367 | 1.35 | 1.12-1.63 | 0.002 | 1.31 | 1.08-1.59 | 0.007 | 1.30 | 1.07-1.58 | 0.009 |
|  | Other | 1.16 | 0.94-1.45 | 0.175 | 1.35 | 1.09-1.67 | 0.007 | 1.32 | 1.06-1.64 | 0.012 | 1.29 | 1.04-1.61 | 0.022 |
| Age at diagnosis | 85-99 |  |  |  | 0.85 | 0.68-1.06 | 0.149 | 0.87 | 0.69-1.09 | 0.230 | 0.87 | 0.69-1.09 | 0.213 |
|  | 30-64 |  |  |  | 0.71 | 0.58-0.87 | 0.001 | 0.70 | 0.57-0.85 | 0.001 | 0.70 | 0.57-0.86 | 0.001 |
|  | 65-74 |  |  |  | 0.82 | 0.7-0.97 | 0.018 | 0.81 | 0.69-0.96 | 0.013 | 0.81 | 0.69-0.96 | 0.014 |
| Gender | Female |  |  |  | 1.19 | 1.04-1.37 | 0.012 | 1.19 | 1.04-1.37 | 0.011 | 1.19 | 1.04-1.37 | 0.011 |
| Ethnicity | Non-White |  |  |  | 0.94 | 0.66-1.34 | 0.741 | 0.95 | 0.67-1.36 | 0.792 | 0.96 | 0.68-1.37 | 0.828 |
|  | Unknown |  |  |  | 0.65 | 0.35-1.2 | 0.169 | 0.67 | 0.36-1.25 | 0.213 | 0.67 | 0.36-1.24 | 0.203 |
| IMD | 2 |  |  |  | 0.88 | 0.7-1.1 | 0.255 | 0.88 | 0.7-1.11 | 0.279 | 0.88 | 0.7-1.11 | 0.277 |
|  | 3 |  |  |  | 0.85 | 0.68-1.06 | 0.147 | 0.85 | 0.68-1.06 | 0.153 | 0.85 | 0.68-1.06 | 0.156 |
|  | 4 |  |  |  | 0.83 | 0.67-1.04 | 0.103 | 0.84 | 0.67-1.05 | 0.117 | 0.84 | 0.67-1.05 | 0.119 |
|  | 5 - Most deprived |  |  |  | 0.83 | 0.67-1.04 | 0.103 | 0.84 | 0.67-1.05 | 0.121 | 0.84 | 0.67-1.05 | 0.117 |
| Smoker status | Non-smoker |  |  |  | 0.79 | 0.59-1.05 | 0.109 | 0.79 | 0.59-1.05 | 0.106 | 0.79 | 0.59-1.06 | 0.113 |
| COPD status | Pre-existing COPD (at -24 months or earlier) |  |  |  | 1.77 | 1.45-2.16 | 0.000 | 1.79 | 1.46-2.18 | 0.000 | 1.79 | 1.46-2.18 | 0.000 |
|  | No COPD |  |  |  | 0.45 | 0.37-0.55 | 0.000 | 0.45 | 0.37-0.55 | 0.000 | 0.45 | 0.37-0.55 | 0.000 |
| Elixhauser comorbidity score | 1 |  |  |  | 1.13 | 0.89-1.43 | 0.313 | 1.13 | 0.89-1.43 | 0.312 | 1.12 | 0.88-1.42 | 0.359 |
|  | 2 |  |  |  | 1.00 | 0.79-1.28 | 0.981 | 1.00 | 0.79-1.28 | 0.984 | 1.00 | 0.78-1.27 | 0.970 |
|  | 3+ |  |  |  | 1.32 | 1.07-1.61 | 0.008 | 1.31 | 1.07-1.61 | 0.009 | 1.31 | 1.07-1.61 | 0.010 |
| Morphology | NSCLC |  |  |  |  |  |  | 1.01 | 0.8-1.27 | 0.957 | 0.98 | 0.77-1.24 | 0.862 |
|  | Unspecified |  |  |  |  |  |  | 0.90 | 0.7-1.17 | 0.447 | 0.87 | 0.67-1.14 | 0.312 |
| Stage at diagnosis | Unknown |  |  |  |  |  |  |  |  |  | 1.11 | 0.95-1.3 | 0.171 |
|  | Not advanced |  |  |  |  |  |  |  |  |  | 1.19 | 0.98-1.44 | 0.080 |

# Cough consultations (-12 to -6)

| **Variable** |  | **IRR** | **95% CI** | **p-value** | **IRR** | **95% CI** | **p-value** | **IRR** | **95% CI** | **p-value** | **IRR** | **95% CI** | **p-value** |
| --- | --- | --- | --- | --- | --- | --- | --- | --- | --- | --- | --- | --- | --- |
| Diagnostic route | GP referral | 1.63 | 1.21-2.19 | 0.001 | 1.77 | 1.31-2.39 | 0.000 | 1.68 | 1.24-2.27 | 0.001 | 1.63 | 1.2-2.22 | 0.002 |
|  | TWW | 1.41 | 1.06-1.88 | 0.017 | 1.61 | 1.2-2.17 | 0.002 | 1.51 | 1.11-2.04 | 0.009 | 1.49 | 1.09-2.02 | 0.012 |
|  | Other | 1.26 | 0.89-1.78 | 0.193 | 1.40 | 0.99-1.97 | 0.056 | 1.33 | 0.94-1.89 | 0.106 | 1.29 | 0.91-1.83 | 0.158 |
| Age at diagnosis | 85-99 |  |  |  | 0.82 | 0.57-1.17 | 0.267 | 0.87 | 0.6-1.25 | 0.441 | 0.86 | 0.6-1.24 | 0.415 |
|  | 30-64 |  |  |  | 0.78 | 0.58-1.06 | 0.115 | 0.75 | 0.55-1.02 | 0.065 | 0.75 | 0.55-1.03 | 0.072 |
|  | 65-74 |  |  |  | 0.79 | 0.6-1.03 | 0.079 | 0.76 | 0.59-1 | 0.048 | 0.77 | 0.59-1 | 0.051 |
| Gender | Female |  |  |  | 1.35 | 1.09-1.68 | 0.006 | 1.37 | 1.1-1.7 | 0.005 | 1.37 | 1.1-1.7 | 0.005 |
| Ethnicity | Non-White |  |  |  | 0.91 | 0.53-1.56 | 0.737 | 0.94 | 0.55-1.61 | 0.814 | 0.95 | 0.56-1.63 | 0.860 |
|  | Unknown |  |  |  | 0.57 | 0.21-1.5 | 0.253 | 0.62 | 0.23-1.65 | 0.342 | 0.62 | 0.23-1.64 | 0.332 |
| IMD | 2 |  |  |  | 0.84 | 0.59-1.2 | 0.351 | 0.86 | 0.6-1.22 | 0.387 | 0.86 | 0.6-1.22 | 0.391 |
|  | 3 |  |  |  | 0.87 | 0.61-1.23 | 0.422 | 0.87 | 0.62-1.23 | 0.442 | 0.88 | 0.62-1.24 | 0.454 |
|  | 4 |  |  |  | 0.85 | 0.6-1.19 | 0.340 | 0.86 | 0.61-1.21 | 0.389 | 0.86 | 0.61-1.22 | 0.406 |
|  | 5 - Most deprived |  |  |  | 0.83 | 0.58-1.17 | 0.286 | 0.84 | 0.6-1.2 | 0.345 | 0.84 | 0.6-1.2 | 0.344 |
| Smoker status | Non-smoker |  |  |  | 0.80 | 0.52-1.21 | 0.283 | 0.79 | 0.52-1.19 | 0.259 | 0.79 | 0.52-1.2 | 0.271 |
| COPD status | Pre-existing COPD (at -24 months or earlier) |  |  |  | 1.10 | 0.78-1.53 | 0.589 | 1.12 | 0.8-1.56 | 0.514 | 1.12 | 0.8-1.56 | 0.513 |
|  | No COPD |  |  |  | 0.58 | 0.42-0.79 | 0.001 | 0.58 | 0.42-0.78 | 0.000 | 0.58 | 0.43-0.79 | 0.001 |
| Elixhauser comorbidity score | 1 |  |  |  | 1.15 | 0.81-1.65 | 0.425 | 1.15 | 0.81-1.64 | 0.438 | 1.13 | 0.79-1.61 | 0.490 |
|  | 2 |  |  |  | 1.08 | 0.75-1.56 | 0.670 | 1.08 | 0.75-1.55 | 0.686 | 1.07 | 0.74-1.54 | 0.718 |
|  | 3+ |  |  |  | 1.24 | 0.91-1.7 | 0.175 | 1.24 | 0.9-1.69 | 0.182 | 1.23 | 0.9-1.69 | 0.189 |
| Morphology | NSCLC |  |  |  |  |  |  | 1.12 | 0.77-1.61 | 0.555 | 1.08 | 0.74-1.56 | 0.696 |
|  | Unspecified |  |  |  |  |  |  | 0.85 | 0.56-1.3 | 0.454 | 0.81 | 0.53-1.24 | 0.337 |
| Stage at diagnosis | Unknown |  |  |  |  |  |  |  |  |  | 1.17 | 0.92-1.5 | 0.198 |
|  | Not advanced |  |  |  |  |  |  |  |  |  | 1.28 | 0.94-1.73 | 0.118 |

# Dyspnoea consultations (-12 to -6)

| **Variable** |  | **IRR** | **95% CI** | **p-value** | **IRR** | **95% CI** | **p-value** | **IRR** | **95% CI** | **p-value** | **IRR** | **95% CI** | **p-value** |
| --- | --- | --- | --- | --- | --- | --- | --- | --- | --- | --- | --- | --- | --- |
| Diagnostic route | GP referral | 1.26 | 0.96-1.66 | 0.096 | 1.53 | 1.19-1.97 | 0.001 | 1.56 | 1.21-2.01 | 0.001 | 1.54 | 1.19-1.99 | 0.001 |
|  | TWW | 0.84 | 0.64-1.11 | 0.213 | 1.09 | 0.84-1.42 | 0.516 | 1.10 | 0.84-1.44 | 0.473 | 1.10 | 0.84-1.44 | 0.504 |
|  | Other | 0.95 | 0.69-1.32 | 0.762 | 1.11 | 0.82-1.49 | 0.509 | 1.12 | 0.83-1.5 | 0.475 | 1.10 | 0.81-1.48 | 0.544 |
| Age at diagnosis | 85-99 |  |  |  | 0.91 | 0.67-1.23 | 0.533 | 0.90 | 0.66-1.23 | 0.528 | 0.90 | 0.66-1.23 | 0.519 |
|  | 30-64 |  |  |  | 0.67 | 0.49-0.9 | 0.009 | 0.67 | 0.5-0.91 | 0.011 | 0.67 | 0.5-0.91 | 0.011 |
|  | 65-74 |  |  |  | 0.90 | 0.72-1.13 | 0.373 | 0.91 | 0.73-1.14 | 0.414 | 0.91 | 0.73-1.14 | 0.426 |
| Gender | Female |  |  |  | 0.99 | 0.82-1.2 | 0.936 | 0.99 | 0.82-1.19 | 0.885 | 0.99 | 0.82-1.19 | 0.896 |
| Ethnicity | Non-White |  |  |  | 0.87 | 0.5-1.51 | 0.622 | 0.86 | 0.5-1.5 | 0.601 | 0.86 | 0.5-1.5 | 0.605 |
|  | Unknown |  |  |  | 0.98 | 0.43-2.22 | 0.957 | 0.97 | 0.43-2.21 | 0.950 | 0.97 | 0.42-2.2 | 0.935 |
| IMD | 2 |  |  |  | 0.92 | 0.67-1.26 | 0.600 | 0.92 | 0.67-1.26 | 0.588 | 0.91 | 0.67-1.26 | 0.578 |
|  | 3 |  |  |  | 0.85 | 0.62-1.17 | 0.329 | 0.85 | 0.62-1.16 | 0.310 | 0.85 | 0.62-1.16 | 0.309 |
|  | 4 |  |  |  | 0.78 | 0.57-1.08 | 0.133 | 0.78 | 0.57-1.07 | 0.127 | 0.78 | 0.57-1.07 | 0.127 |
|  | 5 - Most deprived |  |  |  | 0.84 | 0.62-1.15 | 0.274 | 0.84 | 0.61-1.14 | 0.257 | 0.83 | 0.61-1.14 | 0.251 |
| Smoker status | Non-smoker |  |  |  | 0.68 | 0.4-1.16 | 0.156 | 0.69 | 0.41-1.18 | 0.173 | 0.69 | 0.41-1.18 | 0.174 |
| COPD status | Pre-existing COPD (at -24 months or earlier) |  |  |  | 2.50 | 1.93-3.23 | 0.000 | 2.50 | 1.93-3.23 | 0.000 | 2.50 | 1.93-3.23 | 0.000 |
|  | No COPD |  |  |  | 0.27 | 0.2-0.36 | 0.000 | 0.27 | 0.2-0.36 | 0.000 | 0.27 | 0.2-0.36 | 0.000 |
| Elixhauser comorbidity score | 1 |  |  |  | 1.19 | 0.82-1.72 | 0.353 | 1.20 | 0.83-1.73 | 0.340 | 1.18 | 0.82-1.71 | 0.366 |
|  | 2 |  |  |  | 1.03 | 0.71-1.5 | 0.861 | 1.04 | 0.72-1.51 | 0.841 | 1.03 | 0.71-1.5 | 0.871 |
|  | 3+ |  |  |  | 1.51 | 1.11-2.05 | 0.009 | 1.51 | 1.11-2.06 | 0.008 | 1.51 | 1.11-2.05 | 0.008 |
| Morphology | NSCLC |  |  |  |  |  |  | 0.83 | 0.6-1.15 | 0.259 | 0.81 | 0.59-1.13 | 0.216 |
|  | Unspecified |  |  |  |  |  |  | 0.88 | 0.62-1.25 | 0.472 | 0.86 | 0.6-1.22 | 0.397 |
| Stage at diagnosis | Unknown |  |  |  |  |  |  |  |  |  | 1.07 | 0.86-1.33 | 0.537 |
|  | Not advanced |  |  |  |  |  |  |  |  |  | 1.12 | 0.86-1.46 | 0.398 |

# Any chest imaging event (-12 to -6)

| **Variable** |  | **IRR** | **95% CI** | **p-value** | **IRR** | **95% CI** | **p-value** | **IRR** | **95% CI** | **p-value** | **IRR** | **95% CI** | **p-value** |
| --- | --- | --- | --- | --- | --- | --- | --- | --- | --- | --- | --- | --- | --- |
| Diagnostic route | GP referral | 1.29 | 0.74-2.25 | 0.369 | 1.77 | 1.01-3.09 | 0.045 | 1.78 | 1.01-3.13 | 0.046 | 1.53 | 0.88-2.65 | 0.132 |
|  | TWW | 0.56 | 0.3-1.06 | 0.074 | 0.84 | 0.44-1.58 | 0.578 | 0.84 | 0.44-1.61 | 0.607 | 0.68 | 0.36-1.28 | 0.236 |
|  | Other | 1.08 | 0.56-2.07 | 0.818 | 1.40 | 0.74-2.67 | 0.302 | 1.41 | 0.74-2.7 | 0.298 | 1.19 | 0.63-2.25 | 0.586 |
| Age at diagnosis | 85-99 |  |  |  | 1.17 | 0.62-2.21 | 0.625 | 1.16 | 0.61-2.21 | 0.650 | 1.18 | 0.64-2.2 | 0.596 |
|  | 30-64 |  |  |  | 0.75 | 0.4-1.41 | 0.376 | 0.76 | 0.4-1.43 | 0.395 | 0.74 | 0.4-1.37 | 0.339 |
|  | 65-74 |  |  |  | 0.67 | 0.39-1.14 | 0.141 | 0.67 | 0.39-1.15 | 0.148 | 0.67 | 0.4-1.14 | 0.141 |
| Gender | Female |  |  |  | 1.20 | 0.78-1.85 | 0.396 | 1.20 | 0.78-1.85 | 0.399 | 1.16 | 0.77-1.76 | 0.476 |
| Ethnicity | Non-White |  |  |  | 0.43 | 0.1-1.83 | 0.252 | 0.43 | 0.1-1.82 | 0.249 | 0.46 | 0.12-1.84 | 0.273 |
|  | Unknown |  |  |  | 1.38 | 0.31-6.18 | 0.677 | 1.36 | 0.3-6.16 | 0.693 | 1.48 | 0.34-6.43 | 0.602 |
| IMD | 2 |  |  |  | 0.98 | 0.48-2.02 | 0.964 | 0.98 | 0.48-2.02 | 0.958 | 0.95 | 0.48-1.9 | 0.887 |
|  | 3 |  |  |  | 0.96 | 0.47-1.96 | 0.906 | 0.96 | 0.47-1.96 | 0.905 | 0.95 | 0.48-1.89 | 0.892 |
|  | 4 |  |  |  | 1.16 | 0.58-2.32 | 0.683 | 1.15 | 0.58-2.31 | 0.687 | 1.17 | 0.6-2.27 | 0.651 |
|  | 5 - Most deprived |  |  |  | 1.05 | 0.52-2.13 | 0.895 | 1.05 | 0.51-2.13 | 0.901 | 1.05 | 0.53-2.08 | 0.879 |
| Smoker status | Non-smoker |  |  |  | 1.02 | 0.46-2.24 | 0.967 | 1.02 | 0.46-2.24 | 0.969 | 1.07 | 0.5-2.28 | 0.860 |
| COPD status | Pre-existing COPD (at -24 months or earlier) |  |  |  | 1.20 | 0.63-2.29 | 0.579 | 1.20 | 0.63-2.28 | 0.586 | 1.13 | 0.6-2.1 | 0.710 |
|  | No COPD |  |  |  | 0.62 | 0.33-1.14 | 0.123 | 0.62 | 0.33-1.14 | 0.124 | 0.62 | 0.34-1.12 | 0.115 |
| Elixhauser comorbidity score | 1 |  |  |  | 1.06 | 0.45-2.48 | 0.892 | 1.06 | 0.45-2.48 | 0.893 | 0.97 | 0.43-2.2 | 0.945 |
|  | 2 |  |  |  | 1.18 | 0.52-2.7 | 0.688 | 1.18 | 0.52-2.71 | 0.687 | 1.08 | 0.49-2.39 | 0.845 |
|  | 3+ |  |  |  | 2.37 | 1.2-4.69 | 0.013 | 2.37 | 1.2-4.69 | 0.013 | 2.27 | 1.18-4.36 | 0.014 |
| Morphology | NSCLC |  |  |  |  |  |  | 1.02 | 0.47-2.19 | 0.965 | 0.83 | 0.39-1.75 | 0.625 |
|  | Unspecified |  |  |  |  |  |  | 1.05 | 0.46-2.43 | 0.901 | 1.01 | 0.45-2.27 | 0.975 |
| Stage at diagnosis | Unknown |  |  |  |  |  |  |  |  |  | 0.50 | 0.3-0.85 | 0.010 |
|  | Not advanced |  |  |  |  |  |  |  |  |  | 2.42 | 1.45-4.06 | 0.001 |

# Chest X-rays (-12 to -6)

| **Variable** |  | **IRR** | **95% CI** | **p-value** | **IRR** | **95% CI** | **p-value** | **IRR** | **95% CI** | **p-value** | **IRR** | **95% CI** | **p-value** |
| --- | --- | --- | --- | --- | --- | --- | --- | --- | --- | --- | --- | --- | --- |
| Diagnostic route | GP referral | 1.14 | 0.62-2.08 | 0.678 | 1.56 | 0.85-2.84 | 0.150 | 1.58 | 0.86-2.91 | 0.142 | 1.38 | 0.75-2.52 | 0.297 |
|  | TWW | 0.60 | 0.31-1.16 | 0.129 | 0.91 | 0.47-1.76 | 0.775 | 0.92 | 0.47-1.82 | 0.819 | 0.77 | 0.4-1.51 | 0.450 |
|  | Other | 0.97 | 0.48-1.97 | 0.935 | 1.26 | 0.63-2.52 | 0.523 | 1.27 | 0.63-2.57 | 0.502 | 1.11 | 0.55-2.22 | 0.770 |
| Age at diagnosis | 85-99 |  |  |  | 1.21 | 0.62-2.36 | 0.576 | 1.19 | 0.6-2.36 | 0.610 | 1.22 | 0.63-2.36 | 0.563 |
|  | 30-64 |  |  |  | 0.74 | 0.38-1.46 | 0.387 | 0.75 | 0.38-1.49 | 0.413 | 0.74 | 0.38-1.44 | 0.373 |
|  | 65-74 |  |  |  | 0.64 | 0.36-1.15 | 0.137 | 0.65 | 0.36-1.16 | 0.146 | 0.65 | 0.37-1.15 | 0.140 |
| Gender | Female |  |  |  | 1.17 | 0.74-1.86 | 0.496 | 1.17 | 0.74-1.86 | 0.506 | 1.14 | 0.72-1.78 | 0.580 |
| Ethnicity | Non-White |  |  |  | 0.50 | 0.11-2.21 | 0.358 | 0.49 | 0.11-2.19 | 0.352 | 0.53 | 0.12-2.26 | 0.390 |
|  | Unknown |  |  |  | 1.61 | 0.35-7.52 | 0.542 | 1.58 | 0.33-7.44 | 0.565 | 1.73 | 0.38-7.94 | 0.479 |
| IMD | 2 |  |  |  | 1.01 | 0.46-2.2 | 0.977 | 1.01 | 0.46-2.2 | 0.985 | 0.98 | 0.46-2.09 | 0.963 |
|  | 3 |  |  |  | 0.99 | 0.46-2.14 | 0.974 | 0.98 | 0.45-2.13 | 0.969 | 0.98 | 0.47-2.08 | 0.966 |
|  | 4 |  |  |  | 1.13 | 0.53-2.4 | 0.753 | 1.12 | 0.53-2.39 | 0.761 | 1.13 | 0.54-2.35 | 0.743 |
|  | 5 - Most deprived |  |  |  | 1.13 | 0.53-2.41 | 0.754 | 1.12 | 0.52-2.4 | 0.768 | 1.13 | 0.54-2.37 | 0.745 |
| Smoker status | Non-smoker |  |  |  | 0.94 | 0.39-2.24 | 0.884 | 0.94 | 0.39-2.26 | 0.895 | 0.98 | 0.42-2.29 | 0.970 |
| COPD status | Pre-existing COPD (at -24 months or earlier) |  |  |  | 1.29 | 0.64-2.58 | 0.472 | 1.28 | 0.64-2.58 | 0.481 | 1.21 | 0.62-2.39 | 0.578 |
|  | No COPD |  |  |  | 0.66 | 0.34-1.28 | 0.215 | 0.66 | 0.34-1.28 | 0.217 | 0.66 | 0.34-1.26 | 0.205 |
| Elixhauser comorbidity score | 1 |  |  |  | 0.93 | 0.36-2.37 | 0.878 | 0.93 | 0.37-2.38 | 0.883 | 0.87 | 0.35-2.16 | 0.759 |
|  | 2 |  |  |  | 1.16 | 0.48-2.82 | 0.740 | 1.16 | 0.48-2.83 | 0.736 | 1.08 | 0.46-2.55 | 0.861 |
|  | 3+ |  |  |  | 2.29 | 1.1-4.75 | 0.026 | 2.29 | 1.11-4.76 | 0.026 | 2.19 | 1.08-4.43 | 0.030 |
| Morphology | NSCLC |  |  |  |  |  |  | 0.91 | 0.41-2.03 | 0.817 | 0.77 | 0.35-1.7 | 0.523 |
|  | Unspecified |  |  |  |  |  |  | 0.98 | 0.41-2.36 | 0.971 | 0.98 | 0.42-2.3 | 0.956 |
| Stage at diagnosis | Unknown |  |  |  |  |  |  |  |  |  | 0.48 | 0.27-0.85 | 0.012 |
|  | Not advanced |  |  |  |  |  |  |  |  |  | 2.01 | 1.15-3.53 | 0.015 |

# Chest CT scans (-12 to -6)

| **Variable** |  | **IRR** | **95% CI** | **p-value** | **IRR** | **95% CI** | **p-value** | **IRR** | **95% CI** | **p-value** | **IRR** | **95% CI** | **p-value** |
| --- | --- | --- | --- | --- | --- | --- | --- | --- | --- | --- | --- | --- | --- |
| Diagnostic route | GP referral | 1.86 | 0.81-4.27 | 0.146 | 2.41 | 0.7-8.34 | 0.164 | 2.32 | 0.66-8.18 | 0.191 | 1.69 | 0.49-5.86 | 0.411 |
|  | TWW | 0.21 | 0.21-0.21 | 0.000 | 0.29 | 0.03-2.94 | 0.298 | 0.28 | 0.03-2.86 | 0.285 | 0.20 | 0.02-1.88 | 0.158 |
|  | Other | 1.58 | 0.53-4.7 | 0.408 | 2.10 | 0.52-8.48 | 0.298 | 2.05 | 0.5-8.35 | 0.319 | 1.43 | 0.36-5.73 | 0.614 |
| Age at diagnosis | 85-99 |  |  |  | 1.15 | 0.25-5.19 | 0.856 | 1.16 | 0.25-5.32 | 0.850 | 1.17 | 0.27-5.05 | 0.829 |
|  | 30-64 |  |  |  | 0.66 | 0.13-3.26 | 0.614 | 0.65 | 0.13-3.23 | 0.601 | 0.63 | 0.13-3.03 | 0.567 |
|  | 65-74 |  |  |  | 0.85 | 0.25-2.89 | 0.797 | 0.84 | 0.25-2.86 | 0.783 | 0.90 | 0.27-2.94 | 0.855 |
| Gender | Female |  |  |  | 1.37 | 0.5-3.77 | 0.547 | 1.39 | 0.5-3.8 | 0.527 | 1.30 | 0.49-3.46 | 0.596 |
| Ethnicity | Non-White |  |  |  | 0.00 | 0-7.97542578435703e+145 | 0.917 | 0.00 | 0-2.09109179509845e+86 | 0.888 | 0.00 | 0-4.52923566137415e+212 | 0.956 |
|  | Unknown |  |  |  | 0.00 | 0-3.51658004755288e+209 | 0.940 | 0.00 | 0-1.943119750568e+147 | 0.930 | 0.00 | 0-2.852729131705e+74 | 0.894 |
| IMD | 2 |  |  |  | 1.01 | 0.18-5.57 | 0.993 | 1.02 | 0.18-5.6 | 0.985 | 0.96 | 0.18-4.97 | 0.958 |
|  | 3 |  |  |  | 0.94 | 0.17-5.23 | 0.944 | 0.95 | 0.17-5.26 | 0.956 | 0.94 | 0.18-4.86 | 0.943 |
|  | 4 |  |  |  | 1.38 | 0.28-6.83 | 0.690 | 1.40 | 0.29-6.89 | 0.676 | 1.42 | 0.3-6.68 | 0.654 |
|  | 5 - Most deprived |  |  |  | 1.00 | 0.18-5.47 | 1.000 | 1.02 | 0.19-5.56 | 0.979 | 1.00 | 0.19-5.16 | 0.998 |
| Smoker status | Non-smoker |  |  |  | 1.23 | 0.21-7.09 | 0.815 | 1.19 | 0.21-6.81 | 0.841 | 1.31 | 0.25-7 | 0.751 |
| COPD status | Pre-existing COPD (at -24 months or earlier) |  |  |  | 0.82 | 0.2-3.37 | 0.782 | 0.83 | 0.2-3.39 | 0.793 | 0.78 | 0.2-3.1 | 0.725 |
|  | No COPD |  |  |  | 0.50 | 0.13-1.94 | 0.319 | 0.50 | 0.13-1.93 | 0.318 | 0.56 | 0.15-2.07 | 0.381 |
| Elixhauser comorbidity score | 1 |  |  |  | 2.04 | 0.22-19.26 | 0.534 | 1.98 | 0.21-18.49 | 0.550 | 1.68 | 0.19-14.58 | 0.639 |
|  | 2 |  |  |  | 1.70 | 0.18-16.48 | 0.646 | 1.67 | 0.17-15.99 | 0.656 | 1.38 | 0.15-12.3 | 0.773 |
|  | 3+ |  |  |  | 3.05 | 0.43-21.56 | 0.264 | 3.02 | 0.43-21.21 | 0.265 | 2.71 | 0.41-17.67 | 0.299 |
| Morphology | NSCLC |  |  |  |  |  |  | 2.01 | 0.19-20.7 | 0.559 | 1.33 | 0.14-12.88 | 0.805 |
|  | Unspecified |  |  |  |  |  |  | 1.73 | 0.15-20.26 | 0.664 | 1.32 | 0.12-14.13 | 0.818 |
| Stage at diagnosis | Unknown |  |  |  |  |  |  |  |  |  | 0.80 | 0.21-3.04 | 0.740 |
|  | Not advanced |  |  |  |  |  |  |  |  |  | 4.54 | 1.44-14.29 | 0.010 |

**Supplementary Table S15.** Modelled incidence rate ratios (IRRs) for pre-diagnostic healthcare use, by diagnostic route, with and without adjustment for socio-demographic and tumour factors **(-6 to -2 months)**.

# Primary care consultations (-6 to -2)

| **Variable** |  | **IRR** | **95% CI** | **p-value** | **IRR** | **95% CI** | **p-value** | **IRR** | **95% CI** | **p-value** | **IRR** | **95% CI** | **p-value** |
| --- | --- | --- | --- | --- | --- | --- | --- | --- | --- | --- | --- | --- | --- |
| Diagnostic route | GP referral | 1.16 | 1.08-1.24 | 0.000 | 1.31 | 1.23-1.4 | 0.000 | 1.31 | 1.23-1.4 | 0.000 | 1.31 | 1.22-1.4 | 0.000 |
|  | TWW | 0.94 | 0.88-1 | 0.067 | 1.13 | 1.06-1.21 | 0.000 | 1.13 | 1.06-1.21 | 0.000 | 1.13 | 1.06-1.21 | 0.000 |
|  | Other | 0.91 | 0.84-0.98 | 0.013 | 1.02 | 0.95-1.1 | 0.577 | 1.02 | 0.95-1.1 | 0.583 | 1.01 | 0.94-1.1 | 0.727 |
| Age at diagnosis | 85-99 |  |  |  | 1.08 | 1-1.17 | 0.049 | 1.08 | 1-1.17 | 0.044 | 1.08 | 1-1.17 | 0.051 |
|  | 30-64 |  |  |  | 0.85 | 0.79-0.91 | 0.000 | 0.84 | 0.79-0.91 | 0.000 | 0.85 | 0.79-0.91 | 0.000 |
|  | 65-74 |  |  |  | 0.95 | 0.89-1.01 | 0.080 | 0.95 | 0.89-1.01 | 0.076 | 0.95 | 0.89-1.01 | 0.079 |
| Gender | Female |  |  |  | 1.10 | 1.05-1.16 | 0.000 | 1.10 | 1.05-1.16 | 0.000 | 1.10 | 1.05-1.16 | 0.000 |
| Ethnicity | Non-White |  |  |  | 0.89 | 0.79-1 | 0.045 | 0.89 | 0.79-1 | 0.048 | 0.89 | 0.79-1 | 0.050 |
|  | Unknown |  |  |  | 0.82 | 0.68-0.99 | 0.036 | 0.82 | 0.68-0.99 | 0.041 | 0.82 | 0.68-0.99 | 0.035 |
| IMD | 2 |  |  |  | 0.95 | 0.88-1.03 | 0.218 | 0.95 | 0.88-1.03 | 0.220 | 0.95 | 0.88-1.03 | 0.217 |
|  | 3 |  |  |  | 1.05 | 0.97-1.13 | 0.271 | 1.04 | 0.97-1.13 | 0.276 | 1.04 | 0.96-1.13 | 0.287 |
|  | 4 |  |  |  | 0.95 | 0.87-1.02 | 0.162 | 0.95 | 0.87-1.02 | 0.160 | 0.95 | 0.87-1.02 | 0.170 |
|  | 5 - Most deprived |  |  |  | 0.88 | 0.81-0.95 | 0.001 | 0.88 | 0.81-0.95 | 0.001 | 0.88 | 0.81-0.95 | 0.001 |
| Smoker status | Non-smoker |  |  |  | 0.95 | 0.87-1.03 | 0.207 | 0.95 | 0.87-1.03 | 0.235 | 0.95 | 0.87-1.03 | 0.236 |
| COPD status | Pre-existing COPD (at -24 months or earlier) |  |  |  | 1.05 | 0.97-1.14 | 0.204 | 1.06 | 0.97-1.15 | 0.197 | 1.06 | 0.97-1.15 | 0.182 |
|  | No COPD |  |  |  | 0.86 | 0.8-0.93 | 0.000 | 0.86 | 0.8-0.93 | 0.000 | 0.86 | 0.8-0.93 | 0.000 |
| Elixhauser comorbidity score | 1 |  |  |  | 1.18 | 1.09-1.28 | 0.000 | 1.19 | 1.09-1.28 | 0.000 | 1.18 | 1.09-1.28 | 0.000 |
|  | 2 |  |  |  | 1.27 | 1.17-1.38 | 0.000 | 1.27 | 1.17-1.38 | 0.000 | 1.27 | 1.17-1.38 | 0.000 |
|  | 3+ |  |  |  | 1.57 | 1.47-1.69 | 0.000 | 1.57 | 1.47-1.69 | 0.000 | 1.58 | 1.47-1.69 | 0.000 |
| Morphology | NSCLC |  |  |  |  |  |  | 0.95 | 0.88-1.04 | 0.266 | 0.95 | 0.87-1.03 | 0.201 |
|  | Unspecified |  |  |  |  |  |  | 0.95 | 0.87-1.04 | 0.283 | 0.93 | 0.85-1.03 | 0.159 |
| Stage at diagnosis | Unknown |  |  |  |  |  |  |  |  |  | 1.08 | 1.03-1.14 | 0.004 |
|  | Not advanced |  |  |  |  |  |  |  |  |  | 1.06 | 0.99-1.14 | 0.112 |

# 6 selected relevant symptoms (-6 to -2)

| **Variable** |  | **IRR** | **95% CI** | **p-value** | **IRR** | **95% CI** | **p-value** | **IRR** | **95% CI** | **p-value** | **IRR** | **95% CI** | **p-value** |
| --- | --- | --- | --- | --- | --- | --- | --- | --- | --- | --- | --- | --- | --- |
| Diagnostic route | GP referral | 1.71 | 1.47-1.97 | 0.000 | 1.75 | 1.51-2.03 | 0.000 | 1.71 | 1.47-1.98 | 0.000 | 1.70 | 1.46-1.98 | 0.000 |
|  | TWW | 1.62 | 1.41-1.87 | 0.000 | 1.72 | 1.48-1.99 | 0.000 | 1.64 | 1.41-1.9 | 0.000 | 1.64 | 1.4-1.9 | 0.000 |
|  | Other | 1.19 | 1-1.42 | 0.044 | 1.23 | 1.04-1.47 | 0.019 | 1.20 | 1.01-1.44 | 0.039 | 1.20 | 1-1.43 | 0.046 |
| Age at diagnosis | 85-99 |  |  |  | 0.93 | 0.78-1.11 | 0.439 | 0.98 | 0.82-1.18 | 0.844 | 0.98 | 0.82-1.18 | 0.834 |
|  | 30-64 |  |  |  | 0.94 | 0.81-1.09 | 0.398 | 0.90 | 0.78-1.05 | 0.186 | 0.90 | 0.78-1.05 | 0.192 |
|  | 65-74 |  |  |  | 0.93 | 0.82-1.07 | 0.308 | 0.91 | 0.8-1.04 | 0.188 | 0.92 | 0.8-1.04 | 0.189 |
| Gender | Female |  |  |  | 1.05 | 0.94-1.17 | 0.387 | 1.05 | 0.94-1.17 | 0.381 | 1.05 | 0.94-1.17 | 0.380 |
| Ethnicity | Non-White |  |  |  | 1.02 | 0.79-1.32 | 0.896 | 1.04 | 0.8-1.34 | 0.786 | 1.04 | 0.8-1.34 | 0.782 |
|  | Unknown |  |  |  | 0.90 | 0.59-1.37 | 0.625 | 0.98 | 0.64-1.49 | 0.924 | 0.98 | 0.64-1.49 | 0.915 |
| IMD | 2 |  |  |  | 0.83 | 0.69-0.99 | 0.035 | 0.83 | 0.7-0.99 | 0.042 | 0.83 | 0.7-0.99 | 0.042 |
|  | 3 |  |  |  | 0.93 | 0.78-1.1 | 0.383 | 0.93 | 0.78-1.1 | 0.388 | 0.93 | 0.78-1.1 | 0.385 |
|  | 4 |  |  |  | 0.82 | 0.69-0.98 | 0.025 | 0.83 | 0.7-0.98 | 0.030 | 0.83 | 0.7-0.98 | 0.031 |
|  | 5 - Most deprived |  |  |  | 0.83 | 0.69-0.98 | 0.030 | 0.83 | 0.7-0.99 | 0.039 | 0.83 | 0.7-0.99 | 0.038 |
| Smoker status | Non-smoker |  |  |  | 1.00 | 0.82-1.21 | 0.980 | 1.01 | 0.83-1.23 | 0.918 | 1.01 | 0.83-1.23 | 0.909 |
| COPD status | Pre-existing COPD (at -24 months or earlier) |  |  |  | 1.11 | 0.94-1.31 | 0.236 | 1.13 | 0.95-1.33 | 0.157 | 1.13 | 0.96-1.33 | 0.154 |
|  | No COPD |  |  |  | 0.57 | 0.49-0.66 | 0.000 | 0.57 | 0.49-0.66 | 0.000 | 0.57 | 0.49-0.66 | 0.000 |
| Elixhauser comorbidity score | 1 |  |  |  | 1.18 | 1-1.4 | 0.051 | 1.19 | 1.01-1.41 | 0.041 | 1.19 | 1-1.41 | 0.044 |
|  | 2 |  |  |  | 1.03 | 0.86-1.23 | 0.732 | 1.04 | 0.87-1.24 | 0.693 | 1.03 | 0.87-1.23 | 0.706 |
|  | 3+ |  |  |  | 1.02 | 0.88-1.2 | 0.753 | 1.03 | 0.88-1.2 | 0.730 | 1.03 | 0.88-1.2 | 0.731 |
| Morphology | NSCLC |  |  |  |  |  |  | 0.81 | 0.69-0.97 | 0.018 | 0.81 | 0.68-0.96 | 0.016 |
|  | Unspecified |  |  |  |  |  |  | 0.69 | 0.56-0.84 | 0.000 | 0.68 | 0.56-0.83 | 0.000 |
| Stage at diagnosis | Unknown |  |  |  |  |  |  |  |  |  | 1.04 | 0.92-1.17 | 0.519 |
|  | Not advanced |  |  |  |  |  |  |  |  |  | 1.04 | 0.89-1.22 | 0.610 |

# 3 selected main respiratory symptoms (-6 to -2)

| **Variable** |  | **IRR** | **95% CI** | **p-value** | **IRR** | **95% CI** | **p-value** | **IRR** | **95% CI** | **p-value** | **IRR** | **95% CI** | **p-value** |
| --- | --- | --- | --- | --- | --- | --- | --- | --- | --- | --- | --- | --- | --- |
| Diagnostic route | GP referral | 1.75 | 1.5-2.05 | 0.000 | 1.79 | 1.52-2.1 | 0.000 | 1.75 | 1.48-2.05 | 0.000 | 1.73 | 1.47-2.04 | 0.000 |
|  | TWW | 1.67 | 1.44-1.94 | 0.000 | 1.76 | 1.5-2.06 | 0.000 | 1.68 | 1.43-1.97 | 0.000 | 1.67 | 1.42-1.96 | 0.000 |
|  | Other | 1.20 | 0.99-1.44 | 0.059 | 1.23 | 1.02-1.49 | 0.031 | 1.20 | 1-1.46 | 0.056 | 1.19 | 0.98-1.44 | 0.074 |
| Age at diagnosis | 85-99 |  |  |  | 0.92 | 0.76-1.12 | 0.408 | 0.97 | 0.8-1.18 | 0.756 | 0.97 | 0.8-1.18 | 0.749 |
|  | 30-64 |  |  |  | 1.00 | 0.85-1.18 | 0.983 | 0.97 | 0.82-1.14 | 0.670 | 0.97 | 0.82-1.14 | 0.688 |
|  | 65-74 |  |  |  | 0.99 | 0.86-1.14 | 0.904 | 0.97 | 0.84-1.12 | 0.700 | 0.97 | 0.84-1.12 | 0.706 |
| Gender | Female |  |  |  | 1.04 | 0.93-1.16 | 0.528 | 1.04 | 0.93-1.16 | 0.526 | 1.04 | 0.92-1.16 | 0.536 |
| Ethnicity | Non-White |  |  |  | 1.06 | 0.81-1.4 | 0.656 | 1.08 | 0.82-1.42 | 0.569 | 1.09 | 0.83-1.43 | 0.557 |
|  | Unknown |  |  |  | 0.83 | 0.52-1.31 | 0.421 | 0.90 | 0.56-1.43 | 0.646 | 0.90 | 0.56-1.42 | 0.641 |
| IMD | 2 |  |  |  | 0.82 | 0.67-0.98 | 0.034 | 0.82 | 0.68-0.99 | 0.040 | 0.82 | 0.68-0.99 | 0.040 |
|  | 3 |  |  |  | 0.91 | 0.76-1.09 | 0.316 | 0.91 | 0.76-1.09 | 0.316 | 0.91 | 0.76-1.09 | 0.315 |
|  | 4 |  |  |  | 0.82 | 0.68-0.99 | 0.035 | 0.83 | 0.69-0.99 | 0.040 | 0.83 | 0.69-0.99 | 0.041 |
|  | 5 - Most deprived |  |  |  | 0.80 | 0.67-0.97 | 0.022 | 0.81 | 0.67-0.98 | 0.028 | 0.81 | 0.67-0.98 | 0.027 |
| Smoker status | Non-smoker |  |  |  | 0.99 | 0.8-1.22 | 0.938 | 1.01 | 0.82-1.24 | 0.951 | 1.01 | 0.82-1.24 | 0.934 |
| COPD status | Pre-existing COPD (at -24 months or earlier) |  |  |  | 1.10 | 0.92-1.31 | 0.283 | 1.12 | 0.94-1.34 | 0.201 | 1.12 | 0.94-1.34 | 0.204 |
|  | No COPD |  |  |  | 0.52 | 0.44-0.61 | 0.000 | 0.52 | 0.44-0.61 | 0.000 | 0.52 | 0.44-0.61 | 0.000 |
| Elixhauser comorbidity score | 1 |  |  |  | 1.15 | 0.96-1.38 | 0.134 | 1.16 | 0.97-1.39 | 0.111 | 1.15 | 0.96-1.38 | 0.122 |
|  | 2 |  |  |  | 1.01 | 0.84-1.22 | 0.921 | 1.02 | 0.84-1.23 | 0.873 | 1.01 | 0.84-1.22 | 0.905 |
|  | 3+ |  |  |  | 0.99 | 0.84-1.17 | 0.922 | 0.99 | 0.84-1.17 | 0.946 | 0.99 | 0.84-1.17 | 0.923 |
| Morphology | NSCLC |  |  |  |  |  |  | 0.80 | 0.66-0.96 | 0.014 | 0.79 | 0.65-0.95 | 0.011 |
|  | Unspecified |  |  |  |  |  |  | 0.68 | 0.55-0.84 | 0.000 | 0.67 | 0.54-0.83 | 0.000 |
| Stage at diagnosis | Unknown |  |  |  |  |  |  |  |  |  | 1.04 | 0.91-1.18 | 0.589 |
|  | Not advanced |  |  |  |  |  |  |  |  |  | 1.08 | 0.92-1.28 | 0.350 |

# Cough consultations (-6 to -2)

| **Variable** |  | **IRR** | **95% CI** | **p-value** | **IRR** | **95% CI** | **p-value** | **IRR** | **95% CI** | **p-value** | **IRR** | **95% CI** | **p-value** |
| --- | --- | --- | --- | --- | --- | --- | --- | --- | --- | --- | --- | --- | --- |
| Diagnostic route | GP referral | 2.02 | 1.62-2.51 | 0.000 | 1.96 | 1.56-2.45 | 0.000 | 1.88 | 1.5-2.36 | 0.000 | 1.86 | 1.48-2.34 | 0.000 |
|  | TWW | 2.19 | 1.78-2.69 | 0.000 | 2.11 | 1.7-2.63 | 0.000 | 1.96 | 1.57-2.45 | 0.000 | 1.95 | 1.55-2.44 | 0.000 |
|  | Other | 1.24 | 0.96-1.62 | 0.103 | 1.21 | 0.93-1.58 | 0.163 | 1.16 | 0.89-1.52 | 0.274 | 1.15 | 0.88-1.51 | 0.315 |
| Age at diagnosis | 85-99 |  |  |  | 0.98 | 0.75-1.29 | 0.892 | 1.06 | 0.81-1.4 | 0.658 | 1.06 | 0.81-1.4 | 0.664 |
|  | 30-64 |  |  |  | 1.19 | 0.96-1.48 | 0.112 | 1.12 | 0.9-1.4 | 0.306 | 1.12 | 0.9-1.4 | 0.297 |
|  | 65-74 |  |  |  | 1.07 | 0.88-1.31 | 0.495 | 1.04 | 0.85-1.26 | 0.728 | 1.04 | 0.85-1.26 | 0.722 |
| Gender | Female |  |  |  | 1.09 | 0.93-1.28 | 0.277 | 1.09 | 0.93-1.28 | 0.268 | 1.09 | 0.93-1.28 | 0.276 |
| Ethnicity | Non-White |  |  |  | 1.15 | 0.8-1.65 | 0.437 | 1.19 | 0.83-1.7 | 0.348 | 1.19 | 0.83-1.71 | 0.339 |
|  | Unknown |  |  |  | 0.55 | 0.27-1.12 | 0.098 | 0.62 | 0.3-1.26 | 0.186 | 0.62 | 0.3-1.26 | 0.184 |
| IMD | 2 |  |  |  | 0.82 | 0.63-1.06 | 0.127 | 0.83 | 0.64-1.07 | 0.145 | 0.83 | 0.64-1.07 | 0.146 |
|  | 3 |  |  |  | 0.93 | 0.73-1.19 | 0.564 | 0.93 | 0.73-1.19 | 0.577 | 0.93 | 0.73-1.19 | 0.576 |
|  | 4 |  |  |  | 0.84 | 0.65-1.07 | 0.164 | 0.84 | 0.66-1.08 | 0.183 | 0.85 | 0.66-1.08 | 0.186 |
|  | 5 - Most deprived |  |  |  | 0.75 | 0.58-0.97 | 0.030 | 0.76 | 0.59-0.98 | 0.038 | 0.76 | 0.59-0.98 | 0.037 |
| Smoker status | Non-smoker |  |  |  | 1.15 | 0.88-1.5 | 0.319 | 1.17 | 0.89-1.52 | 0.259 | 1.17 | 0.9-1.53 | 0.250 |
| COPD status | Pre-existing COPD (at -24 months or earlier) |  |  |  | 0.78 | 0.6-1.01 | 0.063 | 0.81 | 0.62-1.04 | 0.101 | 0.81 | 0.62-1.04 | 0.100 |
|  | No COPD |  |  |  | 0.65 | 0.52-0.82 | 0.000 | 0.65 | 0.52-0.81 | 0.000 | 0.65 | 0.52-0.81 | 0.000 |
| Elixhauser comorbidity score | 1 |  |  |  | 1.09 | 0.85-1.38 | 0.496 | 1.10 | 0.86-1.39 | 0.448 | 1.09 | 0.86-1.39 | 0.465 |
|  | 2 |  |  |  | 0.97 | 0.75-1.24 | 0.795 | 0.97 | 0.76-1.25 | 0.815 | 0.97 | 0.75-1.24 | 0.796 |
|  | 3+ |  |  |  | 0.91 | 0.73-1.13 | 0.385 | 0.91 | 0.73-1.13 | 0.384 | 0.90 | 0.72-1.13 | 0.378 |
| Morphology | NSCLC |  |  |  |  |  |  | 0.75 | 0.59-0.96 | 0.020 | 0.74 | 0.58-0.95 | 0.017 |
|  | Unspecified |  |  |  |  |  |  | 0.57 | 0.42-0.76 | 0.000 | 0.56 | 0.42-0.75 | 0.000 |
| Stage at diagnosis | Unknown |  |  |  |  |  |  |  |  |  | 1.04 | 0.87-1.24 | 0.676 |
|  | Not advanced |  |  |  |  |  |  |  |  |  | 1.08 | 0.86-1.36 | 0.496 |

# Dyspnoea consultations (-6 to -2)

| **Variable** |  | **IRR** | **95% CI** | **p-value** | **IRR** | **95% CI** | **p-value** | **IRR** | **95% CI** | **p-value** | **IRR** | **95% CI** | **p-value** |
| --- | --- | --- | --- | --- | --- | --- | --- | --- | --- | --- | --- | --- | --- |
| Diagnostic route | GP referral | 1.25 | 0.95-1.65 | 0.113 | 1.31 | 1-1.72 | 0.054 | 1.33 | 1.01-1.76 | 0.043 | 1.33 | 1.01-1.77 | 0.043 |
|  | TWW | 0.92 | 0.7-1.21 | 0.545 | 1.04 | 0.79-1.37 | 0.798 | 1.05 | 0.79-1.39 | 0.742 | 1.05 | 0.79-1.4 | 0.727 |
|  | Other | 1.06 | 0.77-1.47 | 0.703 | 1.15 | 0.84-1.57 | 0.376 | 1.16 | 0.85-1.59 | 0.342 | 1.17 | 0.85-1.6 | 0.340 |
| Age at diagnosis | 85-99 |  |  |  | 0.85 | 0.61-1.2 | 0.358 | 0.85 | 0.61-1.2 | 0.353 | 0.85 | 0.6-1.2 | 0.349 |
|  | 30-64 |  |  |  | 0.78 | 0.58-1.06 | 0.110 | 0.79 | 0.58-1.07 | 0.125 | 0.79 | 0.58-1.07 | 0.126 |
|  | 65-74 |  |  |  | 0.96 | 0.75-1.22 | 0.715 | 0.96 | 0.75-1.23 | 0.761 | 0.96 | 0.75-1.23 | 0.757 |
| Gender | Female |  |  |  | 1.07 | 0.88-1.31 | 0.492 | 1.07 | 0.87-1.31 | 0.524 | 1.07 | 0.87-1.31 | 0.519 |
| Ethnicity | Non-White |  |  |  | 0.79 | 0.46-1.37 | 0.403 | 0.79 | 0.46-1.37 | 0.401 | 0.79 | 0.46-1.37 | 0.396 |
|  | Unknown |  |  |  | 1.50 | 0.74-3.04 | 0.256 | 1.49 | 0.73-3.03 | 0.274 | 1.48 | 0.73-3.02 | 0.277 |
| IMD | 2 |  |  |  | 0.86 | 0.62-1.21 | 0.398 | 0.86 | 0.62-1.21 | 0.392 | 0.86 | 0.61-1.21 | 0.391 |
|  | 3 |  |  |  | 0.89 | 0.64-1.24 | 0.490 | 0.89 | 0.64-1.23 | 0.475 | 0.89 | 0.64-1.23 | 0.471 |
|  | 4 |  |  |  | 0.85 | 0.61-1.19 | 0.351 | 0.85 | 0.61-1.18 | 0.336 | 0.85 | 0.61-1.18 | 0.336 |
|  | 5 - Most deprived |  |  |  | 0.90 | 0.65-1.25 | 0.523 | 0.89 | 0.65-1.24 | 0.501 | 0.89 | 0.64-1.24 | 0.496 |
| Smoker status | Non-smoker |  |  |  | 0.70 | 0.45-1.1 | 0.124 | 0.71 | 0.45-1.12 | 0.141 | 0.71 | 0.45-1.12 | 0.141 |
| COPD status | Pre-existing COPD (at -24 months or earlier) |  |  |  | 1.48 | 1.12-1.95 | 0.005 | 1.48 | 1.12-1.95 | 0.006 | 1.48 | 1.12-1.96 | 0.005 |
|  | No COPD |  |  |  | 0.29 | 0.22-0.39 | 0.000 | 0.29 | 0.22-0.39 | 0.000 | 0.29 | 0.22-0.39 | 0.000 |
| Elixhauser comorbidity score | 1 |  |  |  | 1.14 | 0.81-1.61 | 0.456 | 1.15 | 0.81-1.62 | 0.434 | 1.15 | 0.81-1.62 | 0.433 |
|  | 2 |  |  |  | 1.08 | 0.76-1.54 | 0.649 | 1.09 | 0.77-1.55 | 0.628 | 1.09 | 0.77-1.55 | 0.624 |
|  | 3+ |  |  |  | 1.08 | 0.8-1.46 | 0.616 | 1.09 | 0.8-1.47 | 0.591 | 1.09 | 0.8-1.47 | 0.585 |
| Morphology | NSCLC |  |  |  |  |  |  | 0.83 | 0.6-1.17 | 0.288 | 0.83 | 0.6-1.17 | 0.296 |
|  | Unspecified |  |  |  |  |  |  | 0.89 | 0.61-1.29 | 0.529 | 0.89 | 0.61-1.29 | 0.526 |
| Stage at diagnosis | Unknown |  |  |  |  |  |  |  |  |  | 1.02 | 0.81-1.28 | 0.850 |
|  | Not advanced |  |  |  |  |  |  |  |  |  | 0.99 | 0.74-1.32 | 0.944 |

# Haemoptysis consultations (-6 to -2)

| **Variable** |  | **IRR** | **95% CI** | **p-value** | **IRR** | **95% CI** | **p-value** | **IRR** | **95% CI** | **p-value** | **IRR** | **95% CI** | **p-value** |
| --- | --- | --- | --- | --- | --- | --- | --- | --- | --- | --- | --- | --- | --- |
| Diagnostic route | GP referral | 3.67 | 1.76-7.65 | 0.001 | 3.95 | 0.08-194.25 | 0.489 | 4.25 | 1.02-17.65 | 0.047 | 4.19 | 1-17.58 | 0.051 |
|  | TWW | 3.28 | 1.64-6.59 | 0.001 | 3.63 | 0.07-179.94 | 0.517 | 3.85 | 0.91-16.32 | 0.067 | 3.85 | 0.9-16.52 | 0.069 |
|  | Other | 1.27 | 0.3-5.5 | 0.745 | 1.50 | 0.01-243.37 | 0.875 | 1.67 | 0.27-10.39 | 0.582 | 1.63 | 0.26-10.25 | 0.603 |
| Age at diagnosis | 85-99 |  |  |  | 1.08 | 0.03-45.23 | 0.970 | 1.22 | 0.29-5.2 | 0.785 | 1.21 | 0.28-5.14 | 0.796 |
|  | 30-64 |  |  |  | 0.76 | 0.03-20.78 | 0.872 | 0.72 | 0.21-2.48 | 0.604 | 0.72 | 0.21-2.5 | 0.610 |
|  | 65-74 |  |  |  | 0.68 | 0.04-11.45 | 0.792 | 0.67 | 0.23-1.97 | 0.464 | 0.66 | 0.22-1.96 | 0.457 |
| Gender | Female |  |  |  | 0.66 | 0.06-7.45 | 0.740 | 0.67 | 0.27-1.66 | 0.386 | 0.67 | 0.27-1.66 | 0.386 |
| Ethnicity | Non-White |  |  |  | 1.03 | 0-275.39 | 0.991 | 1.19 | 0.14-9.88 | 0.874 | 1.20 | 0.14-9.96 | 0.869 |
|  | Unknown |  |  |  | 0.49 | 0-92320.32 | 0.909 | 0.67 | 0.01-67.36 | 0.863 | 0.68 | 0.01-67.12 | 0.869 |
| IMD | 2 |  |  |  | 0.67 | 0.01-35.94 | 0.842 | 0.76 | 0.17-3.42 | 0.716 | 0.76 | 0.17-3.43 | 0.718 |
|  | 3 |  |  |  | 1.09 | 0.03-34.29 | 0.962 | 1.24 | 0.32-4.78 | 0.752 | 1.25 | 0.32-4.81 | 0.749 |
|  | 4 |  |  |  | 0.65 | 0.01-30.49 | 0.824 | 0.75 | 0.17-3.27 | 0.701 | 0.75 | 0.17-3.3 | 0.707 |
|  | 5 - Most deprived |  |  |  | 0.97 | 0.03-35.64 | 0.987 | 1.11 | 0.27-4.48 | 0.887 | 1.10 | 0.27-4.48 | 0.889 |
| Smoker status | Non-smoker |  |  |  | 0.69 | 0-132.87 | 0.892 | 0.73 | 0.12-4.52 | 0.731 | 0.72 | 0.11-4.54 | 0.729 |
| COPD status | Pre-existing COPD (at -24 months or earlier) |  |  |  | 0.99 | 0.03-32.93 | 0.997 | 1.15 | 0.29-4.55 | 0.842 | 1.16 | 0.29-4.61 | 0.831 |
|  | No COPD |  |  |  | 0.67 | 0.03-17.71 | 0.809 | 0.75 | 0.21-2.68 | 0.664 | 0.77 | 0.22-2.75 | 0.685 |
| Elixhauser comorbidity score | 1 |  |  |  | 1.48 | 0.04-55.71 | 0.833 | 1.60 | 0.41-6.27 | 0.502 | 1.57 | 0.4-6.18 | 0.522 |
|  | 2 |  |  |  | 1.13 | 0.02-54.47 | 0.949 | 1.25 | 0.28-5.57 | 0.771 | 1.24 | 0.28-5.55 | 0.778 |
|  | 3+ |  |  |  | 1.28 | 0.04-38.44 | 0.886 | 1.48 | 0.41-5.38 | 0.553 | 1.48 | 0.4-5.42 | 0.553 |
| Morphology | NSCLC |  |  |  |  |  |  | 1.25 | 0.27-5.68 | 0.775 | 1.21 | 0.26-5.61 | 0.810 |
|  | Unspecified |  |  |  |  |  |  | 0.69 | 0.11-4.38 | 0.693 | 0.65 | 0.1-4.22 | 0.654 |
| Stage at diagnosis | Unknown |  |  |  |  |  |  |  |  |  | 1.28 | 0.48-3.44 | 0.626 |
|  | Not advanced |  |  |  |  |  |  |  |  |  | 1.25 | 0.38-4.1 | 0.716 |

# Any chest imaging event (-6 to -2)

| **Variable** |  | **IRR** | **95% CI** | **p-value** | **IRR** | **95% CI** | **p-value** | **IRR** | **95% CI** | **p-value** | **IRR** | **95% CI** | **p-value** |
| --- | --- | --- | --- | --- | --- | --- | --- | --- | --- | --- | --- | --- | --- |
| Diagnostic route | GP referral | 2.19 | 1.75-2.73 | 0 | 2.58 | 2.06-3.23 | 0.000 | 2.34 | 1.87-2.94 | 0.000 | 1.94 | 1.55-2.41 | 0.000 |
|  | TWW | 1.73 | 1.39-2.14 | 0 | 2.22 | 1.77-2.78 | 0.000 | 1.95 | 1.55-2.45 | 0.000 | 1.52 | 1.22-1.9 | 0.000 |
|  | Other | 1.61 | 1.25-2.07 | 0 | 1.85 | 1.43-2.39 | 0.000 | 1.68 | 1.3-2.18 | 0.000 | 1.43 | 1.11-1.84 | 0.005 |
| Age at diagnosis | 85-99 |  |  |  | 1.10 | 0.85-1.43 | 0.466 | 1.25 | 0.96-1.63 | 0.095 | 1.29 | 1-1.66 | 0.050 |
|  | 30-64 |  |  |  | 0.98 | 0.78-1.23 | 0.841 | 0.90 | 0.71-1.13 | 0.350 | 0.89 | 0.71-1.1 | 0.278 |
|  | 65-74 |  |  |  | 0.94 | 0.77-1.15 | 0.575 | 0.89 | 0.73-1.09 | 0.253 | 0.91 | 0.75-1.1 | 0.314 |
| Gender | Female |  |  |  | 1.06 | 0.9-1.25 | 0.462 | 1.08 | 0.92-1.26 | 0.369 | 1.02 | 0.88-1.19 | 0.774 |
| Ethnicity | Non-White |  |  |  | 0.72 | 0.48-1.1 | 0.127 | 0.76 | 0.5-1.16 | 0.204 | 0.80 | 0.53-1.2 | 0.284 |
|  | Unknown |  |  |  | 0.77 | 0.41-1.46 | 0.431 | 0.95 | 0.5-1.81 | 0.888 | 1.10 | 0.59-2.04 | 0.764 |
| IMD | 2 |  |  |  | 0.92 | 0.71-1.2 | 0.541 | 0.95 | 0.73-1.23 | 0.680 | 0.94 | 0.73-1.2 | 0.602 |
|  | 3 |  |  |  | 0.90 | 0.7-1.17 | 0.441 | 0.92 | 0.71-1.18 | 0.501 | 0.92 | 0.72-1.17 | 0.490 |
|  | 4 |  |  |  | 0.88 | 0.68-1.14 | 0.333 | 0.90 | 0.7-1.17 | 0.439 | 0.89 | 0.69-1.14 | 0.349 |
|  | 5 - Most deprived |  |  |  | 0.84 | 0.65-1.09 | 0.193 | 0.88 | 0.68-1.14 | 0.340 | 0.90 | 0.7-1.15 | 0.389 |
| Smoker status | Non-smoker |  |  |  | 1.24 | 0.95-1.63 | 0.118 | 1.23 | 0.93-1.61 | 0.143 | 1.33 | 1.02-1.73 | 0.033 |
| COPD status | Pre-existing COPD (at -24 months or earlier) |  |  |  | 1.02 | 0.79-1.32 | 0.881 | 1.06 | 0.82-1.38 | 0.632 | 0.97 | 0.75-1.24 | 0.786 |
|  | No COPD |  |  |  | 0.68 | 0.54-0.85 | 0.001 | 0.67 | 0.53-0.84 | 0.001 | 0.68 | 0.54-0.85 | 0.001 |
| Elixhauser comorbidity score | 1 |  |  |  | 1.35 | 1.04-1.76 | 0.025 | 1.33 | 1.02-1.73 | 0.033 | 1.25 | 0.97-1.62 | 0.081 |
|  | 2 |  |  |  | 1.19 | 0.91-1.57 | 0.203 | 1.18 | 0.9-1.55 | 0.229 | 1.09 | 0.84-1.42 | 0.520 |
|  | 3+ |  |  |  | 1.85 | 1.46-2.34 | 0.000 | 1.83 | 1.45-2.31 | 0.000 | 1.68 | 1.34-2.1 | 0.000 |
| Morphology | NSCLC |  |  |  |  |  |  | 1.19 | 0.91-1.57 | 0.202 | 0.98 | 0.75-1.28 | 0.873 |
|  | Unspecified |  |  |  |  |  |  | 0.68 | 0.5-0.94 | 0.018 | 0.74 | 0.54-1 | 0.050 |
| Stage at diagnosis | Unknown |  |  |  |  |  |  |  |  |  | 0.23 | 0.18-0.29 | 0.000 |
|  | Not advanced |  |  |  |  |  |  |  |  |  | 2.12 | 1.75-2.56 | 0.000 |

# Chest X-rays (-6 to -2)

| **Variable** |  | **IRR** | **95% CI** | **p-value** | **IRR** | **95% CI** | **p-value** | **IRR** | **95% CI** | **p-value** | **IRR** | **95% CI** | **p-value** |
| --- | --- | --- | --- | --- | --- | --- | --- | --- | --- | --- | --- | --- | --- |
| Diagnostic route | GP referral | 1.71 | 1.36-2.15 | 0.000 | 2.01 | 1.59-2.55 | 0.000 | 1.88 | 1.48-2.38 | 0.000 | 1.65 | 1.31-2.09 | 0.000 |
|  | TWW | 1.58 | 1.27-1.97 | 0.000 | 2.01 | 1.59-2.53 | 0.000 | 1.81 | 1.43-2.3 | 0.000 | 1.49 | 1.18-1.88 | 0.001 |
|  | Other | 1.30 | 1-1.7 | 0.051 | 1.50 | 1.15-1.96 | 0.003 | 1.40 | 1.07-1.84 | 0.015 | 1.26 | 0.96-1.65 | 0.089 |
| Age at diagnosis | 85-99 |  |  |  | 1.21 | 0.93-1.57 | 0.166 | 1.34 | 1.02-1.76 | 0.033 | 1.39 | 1.07-1.82 | 0.014 |
|  | 30-64 |  |  |  | 1.01 | 0.8-1.28 | 0.943 | 0.94 | 0.74-1.19 | 0.610 | 0.93 | 0.73-1.17 | 0.513 |
|  | 65-74 |  |  |  | 0.92 | 0.74-1.13 | 0.417 | 0.88 | 0.71-1.08 | 0.218 | 0.88 | 0.72-1.09 | 0.241 |
| Gender | Female |  |  |  | 1.02 | 0.86-1.2 | 0.846 | 1.03 | 0.87-1.22 | 0.745 | 0.99 | 0.84-1.16 | 0.877 |
| Ethnicity | Non-White |  |  |  | 0.70 | 0.45-1.09 | 0.118 | 0.73 | 0.47-1.14 | 0.170 | 0.77 | 0.5-1.19 | 0.238 |
|  | Unknown |  |  |  | 0.84 | 0.44-1.62 | 0.605 | 0.99 | 0.51-1.92 | 0.981 | 1.17 | 0.62-2.23 | 0.626 |
| IMD | 2 |  |  |  | 0.93 | 0.71-1.22 | 0.601 | 0.95 | 0.72-1.24 | 0.695 | 0.94 | 0.72-1.23 | 0.655 |
|  | 3 |  |  |  | 0.88 | 0.67-1.15 | 0.337 | 0.88 | 0.67-1.15 | 0.360 | 0.88 | 0.68-1.14 | 0.329 |
|  | 4 |  |  |  | 0.84 | 0.64-1.1 | 0.207 | 0.85 | 0.65-1.12 | 0.252 | 0.84 | 0.65-1.09 | 0.196 |
|  | 5 - Most deprived |  |  |  | 0.86 | 0.66-1.13 | 0.293 | 0.89 | 0.68-1.17 | 0.412 | 0.91 | 0.7-1.18 | 0.479 |
| Smoker status | Non-smoker |  |  |  | 1.25 | 0.95-1.67 | 0.116 | 1.25 | 0.94-1.66 | 0.123 | 1.34 | 1.02-1.76 | 0.038 |
| COPD status | Pre-existing COPD (at -24 months or earlier) |  |  |  | 1.01 | 0.77-1.31 | 0.968 | 1.04 | 0.8-1.36 | 0.767 | 0.95 | 0.73-1.23 | 0.688 |
|  | No COPD |  |  |  | 0.66 | 0.52-0.85 | 0.001 | 0.66 | 0.52-0.84 | 0.001 | 0.65 | 0.52-0.83 | 0.000 |
| Elixhauser comorbidity score | 1 |  |  |  | 1.17 | 0.89-1.54 | 0.260 | 1.16 | 0.88-1.53 | 0.289 | 1.12 | 0.86-1.47 | 0.403 |
|  | 2 |  |  |  | 1.02 | 0.76-1.36 | 0.893 | 1.01 | 0.76-1.35 | 0.930 | 0.95 | 0.72-1.27 | 0.745 |
|  | 3+ |  |  |  | 1.64 | 1.29-2.09 | 0.000 | 1.62 | 1.27-2.07 | 0.000 | 1.53 | 1.2-1.93 | 0.000 |
| Morphology | NSCLC |  |  |  |  |  |  | 1.02 | 0.77-1.35 | 0.895 | 0.90 | 0.68-1.19 | 0.459 |
|  | Unspecified |  |  |  |  |  |  | 0.66 | 0.48-0.92 | 0.013 | 0.74 | 0.54-1.02 | 0.070 |
| Stage at diagnosis | Unknown |  |  |  |  |  |  |  |  |  | 0.23 | 0.18-0.29 | 0.000 |
|  | Not advanced |  |  |  |  |  |  |  |  |  | 1.59 | 1.3-1.94 | 0.000 |

# Chest CT scans (-6 to -2)

| **Variable** |  | **IRR** | **95% CI** | **p-value** | **IRR** | **95% CI** | **p-value** | **IRR** | **95% CI** | **p-value** | **IRR** | **95% CI** | **p-value** |
| --- | --- | --- | --- | --- | --- | --- | --- | --- | --- | --- | --- | --- | --- |
| Diagnostic route | GP referral | 3.14 | 2.27-4.35 | 0 | 3.59 | 2.58-4.99 | 0.000 | 3.11 | 2.24-4.32 | 0.000 | 2.32 | 1.75-3.07 | 0.000 |
|  | TWW | 1.97 | 1.41-2.74 | 0 | 2.46 | 1.75-3.48 | 0.000 | 2.07 | 1.46-2.92 | 0.000 | 1.54 | 1.14-2.07 | 0.005 |
|  | Other | 2.47 | 1.71-3.56 | 0 | 2.75 | 1.9-3.97 | 0.000 | 2.41 | 1.67-3.48 | 0.000 | 1.85 | 1.35-2.52 | 0.000 |
| Age at diagnosis | 85-99 |  |  |  | 0.81 | 0.55-1.21 | 0.305 | 0.95 | 0.64-1.41 | 0.784 | 1.00 | 0.72-1.38 | 0.986 |
|  | 30-64 |  |  |  | 0.91 | 0.66-1.25 | 0.555 | 0.82 | 0.6-1.13 | 0.227 | 0.82 | 0.63-1.06 | 0.135 |
|  | 65-74 |  |  |  | 1.02 | 0.78-1.35 | 0.869 | 0.95 | 0.72-1.24 | 0.690 | 0.97 | 0.78-1.21 | 0.804 |
| Gender | Female |  |  |  | 1.14 | 0.9-1.43 | 0.276 | 1.16 | 0.92-1.45 | 0.210 | 1.07 | 0.89-1.28 | 0.491 |
| Ethnicity | Non-White |  |  |  | 0.80 | 0.44-1.47 | 0.474 | 0.86 | 0.47-1.57 | 0.624 | 0.94 | 0.56-1.58 | 0.803 |
|  | Unknown |  |  |  | 0.84 | 0.33-2.12 | 0.711 | 1.17 | 0.47-2.91 | 0.737 | 1.28 | 0.6-2.74 | 0.520 |
| IMD | 2 |  |  |  | 0.96 | 0.66-1.39 | 0.826 | 1.00 | 0.69-1.44 | 0.989 | 0.99 | 0.74-1.33 | 0.939 |
|  | 3 |  |  |  | 0.98 | 0.68-1.41 | 0.912 | 1.01 | 0.71-1.44 | 0.961 | 1.01 | 0.76-1.35 | 0.939 |
|  | 4 |  |  |  | 1.01 | 0.7-1.45 | 0.959 | 1.05 | 0.74-1.5 | 0.774 | 1.09 | 0.82-1.45 | 0.549 |
|  | 5 - Most deprived |  |  |  | 0.77 | 0.53-1.13 | 0.180 | 0.82 | 0.57-1.2 | 0.315 | 0.85 | 0.62-1.15 | 0.293 |
| Smoker status | Non-smoker |  |  |  | 1.17 | 0.79-1.73 | 0.441 | 1.13 | 0.76-1.66 | 0.550 | 1.16 | 0.84-1.59 | 0.361 |
| COPD status | Pre-existing COPD (at -24 months or earlier) |  |  |  | 0.92 | 0.64-1.32 | 0.634 | 0.97 | 0.68-1.39 | 0.861 | 0.95 | 0.72-1.26 | 0.734 |
|  | No COPD |  |  |  | 0.75 | 0.54-1.04 | 0.086 | 0.74 | 0.54-1.02 | 0.068 | 0.84 | 0.65-1.09 | 0.181 |
| Elixhauser comorbidity score | 1 |  |  |  | 1.70 | 1.15-2.49 | 0.007 | 1.65 | 1.13-2.41 | 0.010 | 1.47 | 1.07-2.03 | 0.018 |
|  | 2 |  |  |  | 1.68 | 1.13-2.49 | 0.010 | 1.65 | 1.12-2.44 | 0.012 | 1.43 | 1.03-1.98 | 0.035 |
|  | 3+ |  |  |  | 2.25 | 1.59-3.19 | 0.000 | 2.21 | 1.57-3.12 | 0.000 | 1.91 | 1.43-2.54 | 0.000 |
| Morphology | NSCLC |  |  |  |  |  |  | 1.97 | 1.26-3.08 | 0.003 | 1.41 | 0.95-2.09 | 0.087 |
|  | Unspecified |  |  |  |  |  |  | 0.86 | 0.51-1.44 | 0.567 | 0.74 | 0.47-1.17 | 0.192 |
| Stage at diagnosis | Unknown |  |  |  |  |  |  |  |  |  | 0.33 | 0.24-0.45 | 0.000 |
|  | Not advanced |  |  |  |  |  |  |  |  |  | 2.96 | 2.43-3.61 | 0.000 |

**Supplementary Table S16.** Modelled incidence rate ratios (IRRs) for pre-diagnostic symptomatic consultations **excluding patients with haemoptysis**, by diagnostic route, with and without adjustment for socio-demographic and tumour factors **(-12 to -2 months)**.

# 6 selected relevant symptoms (-12 to -2)

| **Variable** |  | **IRR** | **95% CI** | **p-value** | **IRR** | **95% CI** | **p-value** | **IRR** | **95% CI** | **p-value** | **IRR** | **95% CI** | **p-value** |
| --- | --- | --- | --- | --- | --- | --- | --- | --- | --- | --- | --- | --- | --- |
| Diagnostic route | GP referral | 1.55 | 1.36-1.76 | 0.000 | 1.65 | 1.45-1.88 | 0.000 | 1.61 | 1.41-1.84 | 0.000 | 1.60 | 1.4-1.83 | 0.000 |
|  | TWW | 1.43 | 1.26-1.62 | 0.000 | 1.60 | 1.41-1.82 | 0.000 | 1.54 | 1.35-1.76 | 0.000 | 1.53 | 1.34-1.75 | 0.000 |
|  | Other | 1.21 | 1.04-1.41 | 0.012 | 1.29 | 1.11-1.5 | 0.001 | 1.26 | 1.09-1.47 | 0.002 | 1.26 | 1.08-1.46 | 0.003 |
| Age at diagnosis | 85-99 |  |  |  | 0.92 | 0.79-1.07 | 0.287 | 0.96 | 0.82-1.13 | 0.621 | 0.96 | 0.82-1.12 | 0.610 |
|  | 30-64 |  |  |  | 0.89 | 0.78-1.02 | 0.088 | 0.86 | 0.75-0.98 | 0.029 | 0.86 | 0.75-0.99 | 0.031 |
|  | 65-74 |  |  |  | 0.93 | 0.83-1.05 | 0.251 | 0.92 | 0.82-1.03 | 0.144 | 0.92 | 0.82-1.03 | 0.146 |
| Gender | Female |  |  |  | 1.08 | 0.98-1.18 | 0.130 | 1.08 | 0.98-1.18 | 0.129 | 1.08 | 0.98-1.18 | 0.129 |
| Ethnicity | Non-White |  |  |  | 1.00 | 0.8-1.26 | 0.981 | 1.02 | 0.81-1.28 | 0.861 | 1.02 | 0.81-1.28 | 0.852 |
|  | Unknown |  |  |  | 0.82 | 0.56-1.18 | 0.285 | 0.88 | 0.6-1.27 | 0.491 | 0.87 | 0.6-1.27 | 0.483 |
| IMD | 2 |  |  |  | 0.86 | 0.74-1.01 | 0.059 | 0.87 | 0.74-1.01 | 0.069 | 0.87 | 0.74-1.01 | 0.068 |
|  | 3 |  |  |  | 0.91 | 0.78-1.05 | 0.205 | 0.91 | 0.78-1.06 | 0.208 | 0.91 | 0.78-1.05 | 0.205 |
|  | 4 |  |  |  | 0.82 | 0.7-0.95 | 0.008 | 0.82 | 0.71-0.96 | 0.011 | 0.82 | 0.71-0.96 | 0.011 |
|  | 5 - Most deprived |  |  |  | 0.85 | 0.73-0.99 | 0.036 | 0.86 | 0.74-1 | 0.048 | 0.86 | 0.74-1 | 0.047 |
| Smoker status | Non-smoker |  |  |  | 0.97 | 0.82-1.16 | 0.752 | 0.98 | 0.83-1.17 | 0.843 | 0.98 | 0.83-1.17 | 0.855 |
| COPD status | Pre-existing COPD (at -24 months or earlier) |  |  |  | 1.37 | 1.19-1.59 | 0.000 | 1.40 | 1.21-1.61 | 0.000 | 1.40 | 1.21-1.61 | 0.000 |
|  | No COPD |  |  |  | 0.53 | 0.47-0.61 | 0.000 | 0.53 | 0.46-0.61 | 0.000 | 0.53 | 0.46-0.61 | 0.000 |
| Elixhauser comorbidity score | 1 |  |  |  | 1.13 | 0.97-1.32 | 0.115 | 1.14 | 0.98-1.33 | 0.098 | 1.13 | 0.97-1.32 | 0.106 |
|  | 2 |  |  |  | 1.01 | 0.86-1.18 | 0.916 | 1.01 | 0.87-1.19 | 0.873 | 1.01 | 0.86-1.18 | 0.897 |
|  | 3+ |  |  |  | 1.12 | 0.98-1.29 | 0.099 | 1.12 | 0.98-1.28 | 0.100 | 1.12 | 0.98-1.28 | 0.101 |
| Morphology | NSCLC |  |  |  |  |  |  | 0.85 | 0.73-0.99 | 0.032 | 0.84 | 0.72-0.98 | 0.026 |
|  | Unspecified |  |  |  |  |  |  | 0.73 | 0.61-0.87 | 0.000 | 0.72 | 0.6-0.86 | 0.000 |
| Stage at diagnosis | Unknown |  |  |  |  |  |  |  |  |  | 1.04 | 0.93-1.15 | 0.521 |
|  | Not advanced |  |  |  |  |  |  |  |  |  | 1.05 | 0.92-1.2 | 0.489 |

# 3 selected main respiratory symptoms (excluding patients with haemoptysis) (-12 to -2)

| **Variable** |  | **IRR** | **95% CI** | **p-value** | **IRR** | **95% CI** | **p-value** | **IRR** | **95% CI** | **p-value** | **IRR** | **95% CI** | **p-value** |
| --- | --- | --- | --- | --- | --- | --- | --- | --- | --- | --- | --- | --- | --- |
| Diagnostic route | GP referral | 1.55 | 1.35-1.78 | 0.000 | 1.64 | 1.43-1.88 | 0.000 | 1.60 | 1.39-1.85 | 0.000 | 1.59 | 1.38-1.84 | 0.000 |
|  | TWW | 1.46 | 1.27-1.66 | 0.000 | 1.63 | 1.42-1.87 | 0.000 | 1.56 | 1.35-1.8 | 0.000 | 1.56 | 1.35-1.79 | 0.000 |
|  | Other | 1.18 | 1-1.38 | 0.044 | 1.26 | 1.07-1.48 | 0.005 | 1.23 | 1.05-1.44 | 0.012 | 1.22 | 1.03-1.43 | 0.018 |
| Age at diagnosis | 85-99 |  |  |  | 0.91 | 0.77-1.07 | 0.254 | 0.95 | 0.8-1.12 | 0.546 | 0.95 | 0.8-1.12 | 0.526 |
|  | 30-64 |  |  |  | 0.92 | 0.79-1.06 | 0.234 | 0.88 | 0.77-1.02 | 0.098 | 0.89 | 0.77-1.02 | 0.104 |
|  | 65-74 |  |  |  | 0.99 | 0.87-1.12 | 0.859 | 0.97 | 0.86-1.1 | 0.631 | 0.97 | 0.86-1.1 | 0.637 |
| Gender | Female |  |  |  | 1.08 | 0.98-1.19 | 0.140 | 1.08 | 0.98-1.19 | 0.138 | 1.08 | 0.98-1.19 | 0.135 |
| Ethnicity | Non-White |  |  |  | 1.05 | 0.82-1.33 | 0.709 | 1.07 | 0.84-1.36 | 0.607 | 1.07 | 0.84-1.36 | 0.603 |
|  | Unknown |  |  |  | 0.76 | 0.51-1.15 | 0.194 | 0.82 | 0.55-1.24 | 0.344 | 0.82 | 0.54-1.23 | 0.332 |
| IMD | 2 |  |  |  | 0.86 | 0.73-1.02 | 0.077 | 0.87 | 0.73-1.02 | 0.089 | 0.87 | 0.73-1.02 | 0.087 |
|  | 3 |  |  |  | 0.88 | 0.75-1.04 | 0.132 | 0.88 | 0.75-1.04 | 0.133 | 0.88 | 0.75-1.04 | 0.128 |
|  | 4 |  |  |  | 0.82 | 0.7-0.97 | 0.019 | 0.83 | 0.71-0.98 | 0.024 | 0.83 | 0.71-0.98 | 0.024 |
|  | 5 - Most deprived |  |  |  | 0.81 | 0.69-0.95 | 0.012 | 0.82 | 0.7-0.96 | 0.015 | 0.82 | 0.69-0.96 | 0.014 |
| Smoker status | Non-smoker |  |  |  | 0.96 | 0.8-1.16 | 0.704 | 0.98 | 0.81-1.18 | 0.798 | 0.98 | 0.81-1.18 | 0.814 |
| COPD status | Pre-existing COPD (at -24 months or earlier) |  |  |  | 1.40 | 1.2-1.63 | 0.000 | 1.42 | 1.22-1.66 | 0.000 | 1.42 | 1.22-1.66 | 0.000 |
|  | No COPD |  |  |  | 0.49 | 0.42-0.56 | 0.000 | 0.48 | 0.42-0.56 | 0.000 | 0.48 | 0.42-0.56 | 0.000 |
| Elixhauser comorbidity score | 1 |  |  |  | 1.10 | 0.94-1.3 | 0.236 | 1.11 | 0.94-1.31 | 0.204 | 1.11 | 0.94-1.3 | 0.222 |
|  | 2 |  |  |  | 0.96 | 0.81-1.14 | 0.666 | 0.97 | 0.82-1.15 | 0.713 | 0.97 | 0.82-1.14 | 0.685 |
|  | 3+ |  |  |  | 1.09 | 0.94-1.26 | 0.240 | 1.09 | 0.94-1.26 | 0.243 | 1.09 | 0.94-1.26 | 0.244 |
| Morphology | NSCLC |  |  |  |  |  |  | 0.83 | 0.71-0.98 | 0.024 | 0.82 | 0.7-0.97 | 0.018 |
|  | Unspecified |  |  |  |  |  |  | 0.71 | 0.59-0.86 | 0.000 | 0.70 | 0.58-0.85 | 0.000 |
| Stage at diagnosis | Unknown |  |  |  |  |  |  |  |  |  | 1.06 | 0.95-1.19 | 0.288 |
|  | Not advanced |  |  |  |  |  |  |  |  |  | 1.07 | 0.92-1.23 | 0.374 |
